# Identification of aging-related genes in diagnosing osteoarthritis via integrating bioinformatics analysis and machine learning

**DOI:** 10.18632/aging.205357

**Published:** 2024-01-03

**Authors:** Jian Huang, Jiangfei Zhou, Xiang Xue, Tianming Dai, Weicong Zhu, Songsong Jiao, Hang Wu, Qingqi Meng

**Affiliations:** 1 Guangzhou Institute of Traumatic Surgery, Guangzhou Red Cross Hospital of Jinan University, Guangzhou 510220, China; 2 Department of Traumatic Orthopedics, The Central Hospital of Xiaogan, Hubei 432100, China; 3 Department of Orthopedics, Guangzhou Red Cross Hospital of Jinan University, Guangzhou 510220, China

**Keywords:** osteoarthritis, aging-related genes, bioinformatics analysis, machine learning algorithms

## Abstract

Background: Osteoarthritis (OA) is one of the main causes of pain and disability in the world, it may be caused by many factors. Aging plays a significant role in the onset and progression of OA. However, the mechanisms underlying it remain unknown. Our research aimed to uncover the role of aging-related genes in the progression of OA.

Methods: In Human OA datasets and aging-related genes were obtained from the GEO database and the HAGR website, respectively. Bioinformatics methods including Gene Ontology (GO), Kyoto Encyclopedia of Genes Genomes (KEGG) pathway enrichment, and Protein-protein interaction (PPI) network analysis were used to analyze differentially expressed aging-related genes (DEARGs) in the normal control group and the OA group. And then weighted gene coexpression network analysis (WGCNA), the least absolute shrinkage and selection operator (LASSO) regression, and the Random Forest (RF) machine learning algorithms were used to find the hub genes.

Results: Four overlapping hub genes: *HMGB2*, *CDKN1A*, *JUN*, and *DDIT3* were identified. According to the nomogram model and receiver operating characteristic (ROC) curve analysis, four hub DEARGs had good diagnostic value in distinguishing normal from OA. Furthermore, the qRT-PCR test demonstrated that *HMGB2*, *CDKN1A*, *JUN*, and *DDIT3* mRNA expression levels were lower in OA group than in normal group.

Conclusion: Finally, these four-hub aging-related genes may help us understand the underlying mechanism of aging in osteoarthritis and could be used as possible diagnostic and therapeutic targets.

## INTRODUCTION

Osteoarthritis (OA) is one of the main causes of pain and disability among the elderly in the world. With the population aging, this disease burdens increasing. The cause of OA is not completely clear. Risk factors include age, obesity, sex, injury, and heredity [1]. More evidence suggests that OA is a disease involving the whole joint, including structural changes in hyaline articular cartilage, subchondral bone, synovium, ligaments, articular capsule, subpatellar fat pad, and muscles around the joint [2]. The traditional diagnosis of OA depends on patients’ symptoms and radiography evaluation, however, there are limited value in the detection of early OA. In the past few years, more and more researchers have devoted themselves to finding biomarkers that can be used for early diagnosis and treatment of OA. Although the practice process is full of challenges, its clinical application is a relatively distant prospect [3].

The increase of senescent cells in diverse tissues is one of the markers of aging. The senescent cells still retain their activity and metabolic capabilities despite losing their ability to divide. It has an effect on the surrounding normal tissues and cells by secreting a large number of pro-inflammatory cytokines, chemokines, Matrix metalloproteinases (MMPs), and angiogenic factors, which form the senescence-associated secretory phenotype (SASP) [4, 5]. In some animal disease model studies, the application of senolytics, a class of drugs that target to induce senescence cell death (quercetin and dasatinib et al.), and senomorphics, a type of SASP inhibitor (apigenin and resveratrol et al.) had achieved satisfactory results and were progressing to the clinical trial stage [6, 7]. As a result, the elimination of senescent cells is considered to be a promising treatment for OA [8].

Bioinformatics and machine learning analyses are critical for understanding the molecular mechanisms of disease and screening key genes [9]. In this study, we screened the differentially expressed aging-related genes (DEARGs) in the normal control group and the OA group by bioinformatic analysis including differential gene analysis and WCGNA, and then LASSO and the Random Forest (RF) algorithm, the machine learning were employed to identify diagnostic biomarkers in OA progression, thereby providing new possibilities for OA therapy.

## RESULTS

### Identification of DEARGs in OA

**Figure 1:**
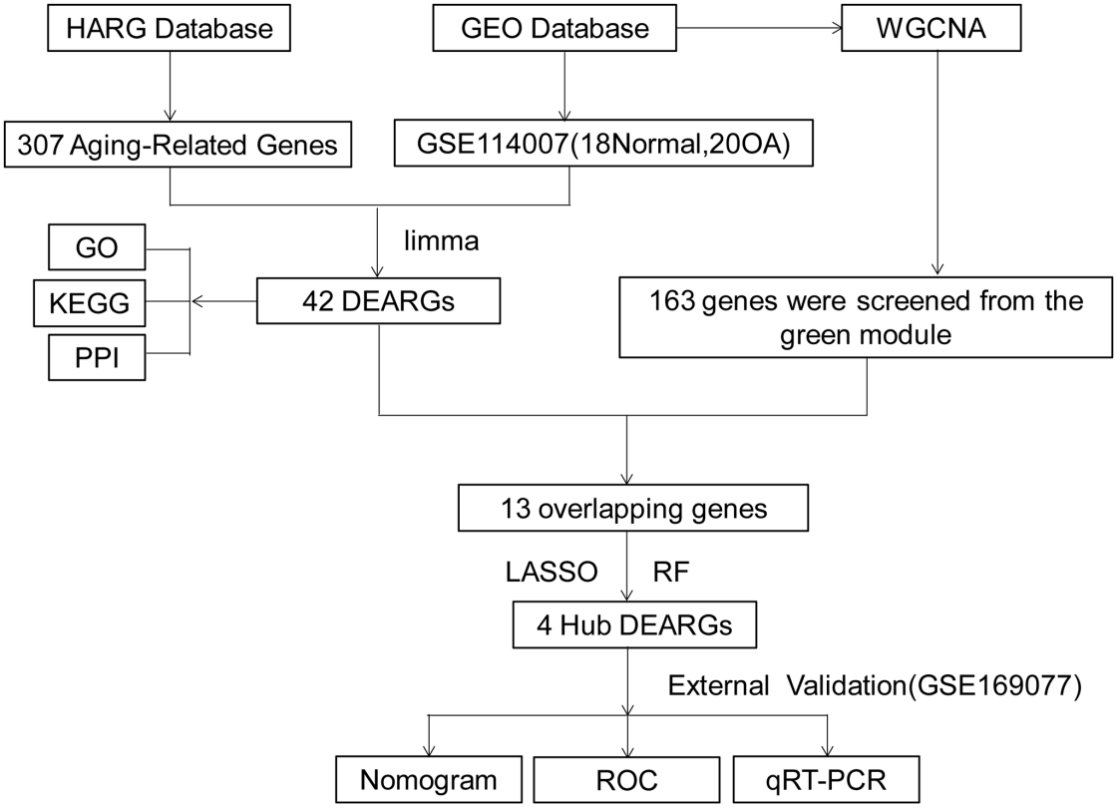
**Work flowchart.** Abbreviations: DEARGs: differentially expressed aging-related genes; LASSO: least absolute shrinkage and selection operator; WGCNA: weighted gene co-expression network analysis.

**Figure 2:**
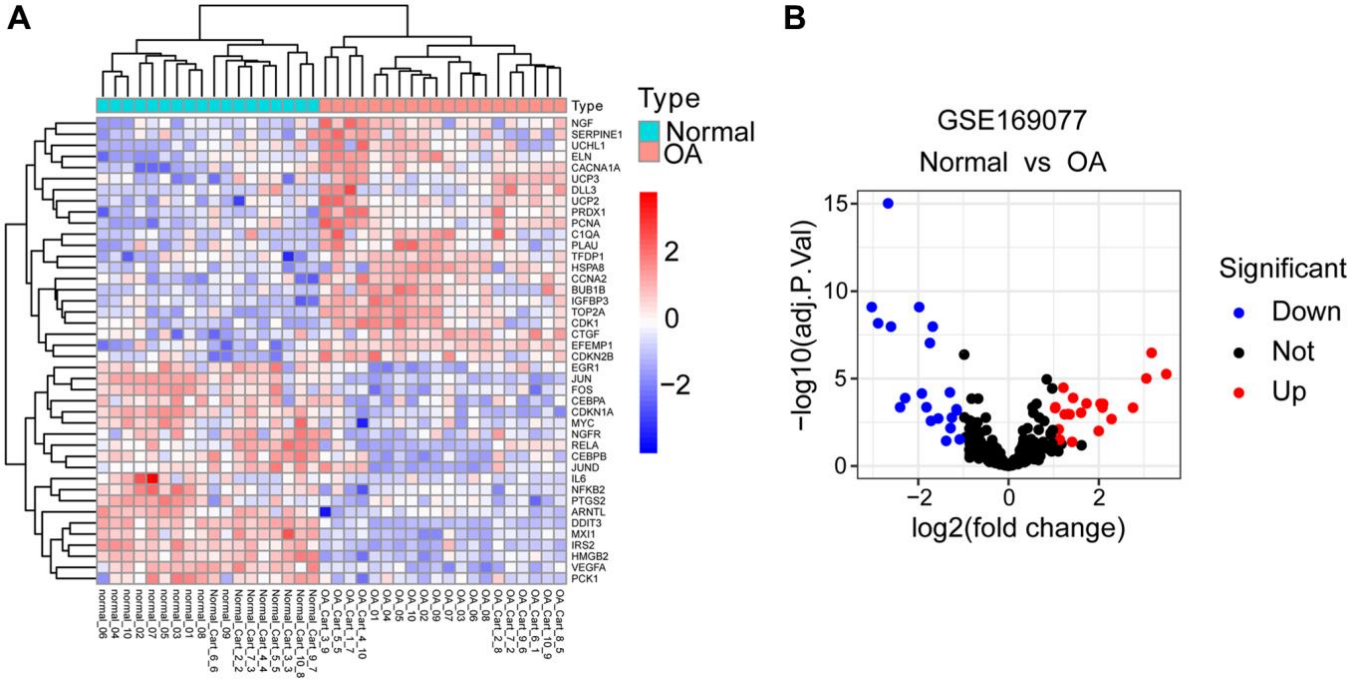
**Identification of DEARGs.** (**A**) Heatmap of DEARGs between normal and OA cartilage tissues. (**B**) Volcano plot for DEARGs between normal and OA cartilage tissues. Red square/plots represent up-regulated genes and blue square/plots represent down-regulated genes.

The study flowchart was depicted in Figure 1. Differential gene analysis was performed using 307 aging-related genes in 20 cases of OA cartilage tissues and 18 cases of normal cartilage tissues, with an adjusted *p*-value of 0.05 and an FC absolute value of >1 as the standard. There were 42 DEARGs in total, with 22 up-regulated genes and 20 down-regulated genes. These DEARGs between the OA group and control group are displayed in the heat map and volcano map (Figure 2A, 2B).

### Functional enrichment and protein-protein interaction analysis of DEARGs

**Figure 3:**
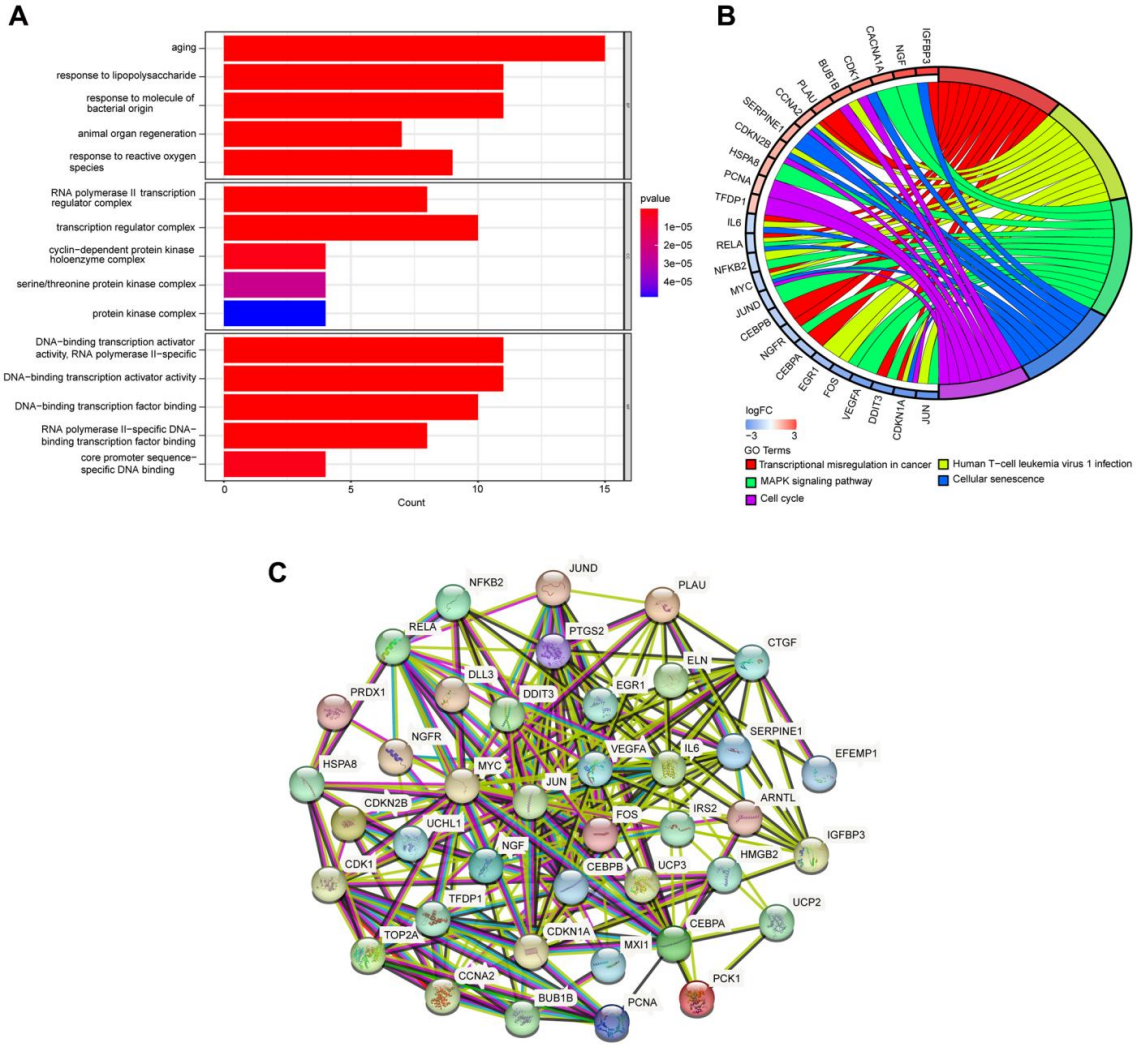
**GO and KEGG pathway enrichment analyses of DEARGs.** (**A**) GO enrichment analyses of DEARGs. (**B**) The connections between DEARGs and the top five enriched KEGG pathways. (**C**) PPI network constructed with the DEARGs.

GO and KEGG enrichment analyses were performed utilizing the R software to identify the potential biological functions of DEARGs. We obtained 1037 and 95 terms for the GO and KEGG enrichment analyses, respectively, based on the screening criterion of adjusted *P*-value 0.05 (Supplementary Tables 1, 2). DEARGs were primarily involved with aging, response to lipopolysaccharide, and response to molecules of bacterial origin (Biological Process, BP). RNA polymerase II transcription regulator complex, transcription regulator complex, cyclin−dependent protein kinase holoenzyme complex (Cellular Component, CC). DNA−binding transcription activator activity RNA polymerase II−specific, DNA−binding transcription activator activity, DNA−binding transcription factor binding (Molecular, Function, MF) (Figure 3A). The most significant KEGG pathways included Transcriptional misregulation in cancer, Human T−cell leukemia virus1 infection, MAPK signaling pathway, Cellular senescence, and Cell cycle, The top five KEGG pathways are shown in Figure 3B. The PPI network uncovered the close interactions between proteins encoded by the DEARGs (Figure 3C).

### Establishment of a co-expression network

**Figure 4:**
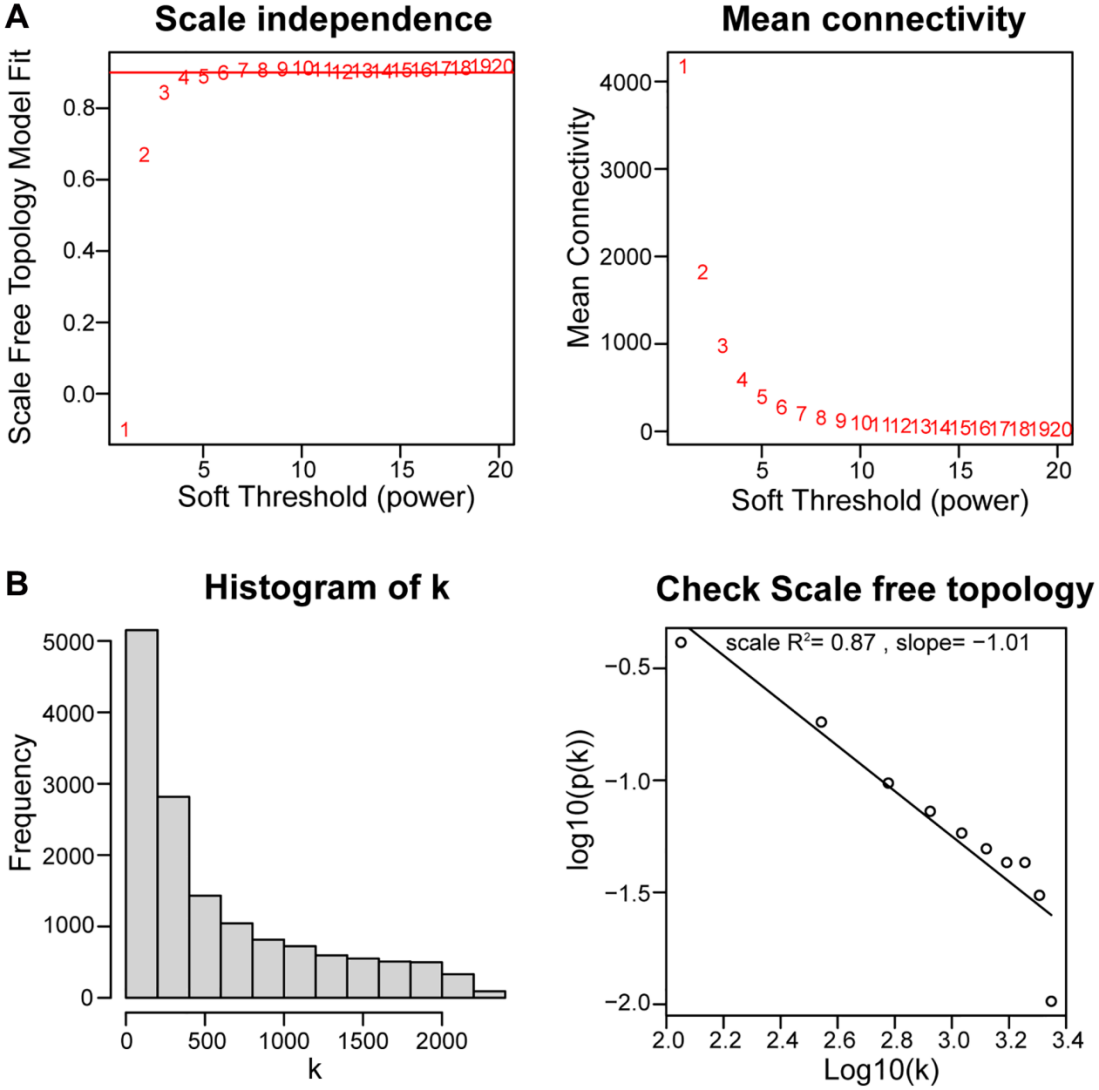
**Using weighted gene coexpression network analysis (WGCNA) to determine soft threshold capability.** (**A**) The soft thresholding power β in the WGCNA was determined based on a scale-free R^2^ (R^2^ = 0.87). (**B**) Histogram of connectivity distribution and checking the scale-free topology when β = 4.

**Figure 5:**
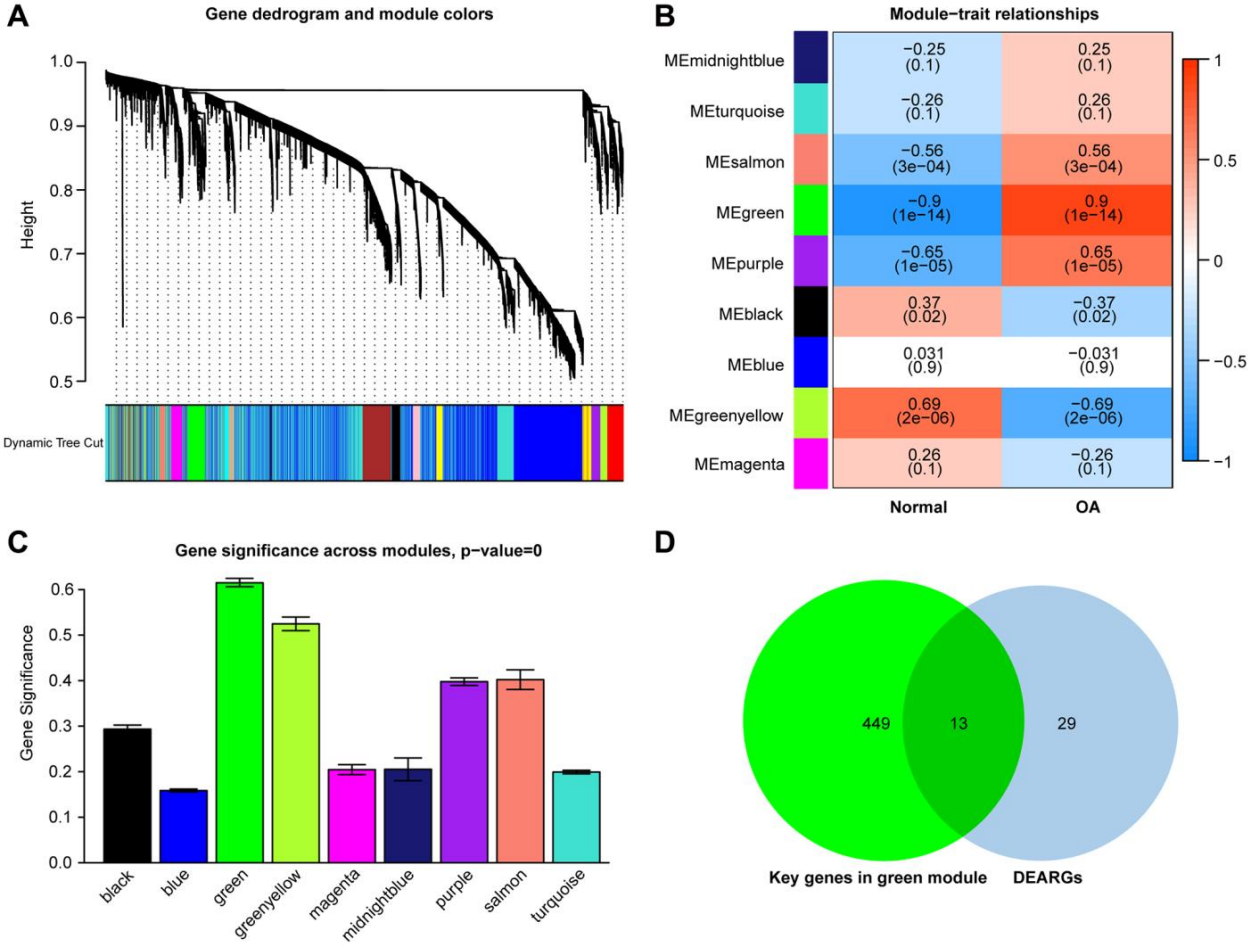
(**A**) Clustering dendrograms of genes with varying degrees of similarity, the different colors below represent various co-expression modules. (**B**) Module–trait relationship. The green module was significantly associated with OA. (**C**) Distribution of mean gene significance in modules associated with OA. (**D**) Venn diagram shows that thirteen common genes are identified from the intersection of genes between the green module and DEARGs.

The WGCNA algorithm was used to identify co-expressed genes and modules based on the expression profiles of all genes. The soft-threshold power of = 4 (R^2^ = 0.87; slope = 1.01) was adopted to ensure that the network was scale-free (Figure 4A, 4B). Then, the co-expression modules in the network were identified by the “cutree Dynamic” function, and 9 gene modules were obtained. The correlations of the above-mentioned modules with OA and healthy controls were presented with heat maps, with green (cor = 0.9; *P* = 1e-14) and greenyellow (cor = −0.69; *P* = 2e-06) modules showing the strongest positive and negative connection with OA, respectively (Figure 5A–5C). In the green module we could gain 462 key genes based on the screening criteria (|GS|> 0.60;|MM|> 0.70). As a result, the 462 key genes in the green module were studied further. Finally, 13 intersecting genes were discovered in the green module between 42 DEARGs and 462 key genes. (Figure 5D). The intersection genes were as follows: *TOP2A, TFDP1, ELN, IGFBP3, EFEMP1, and NGF. JUN, ARNTL, CDKN1, AMXI1, DDIT3, HMGB2*, and *IRS2*.

### Identification of candidate Hub aging-related genes via machine learning

**Figure 6:**
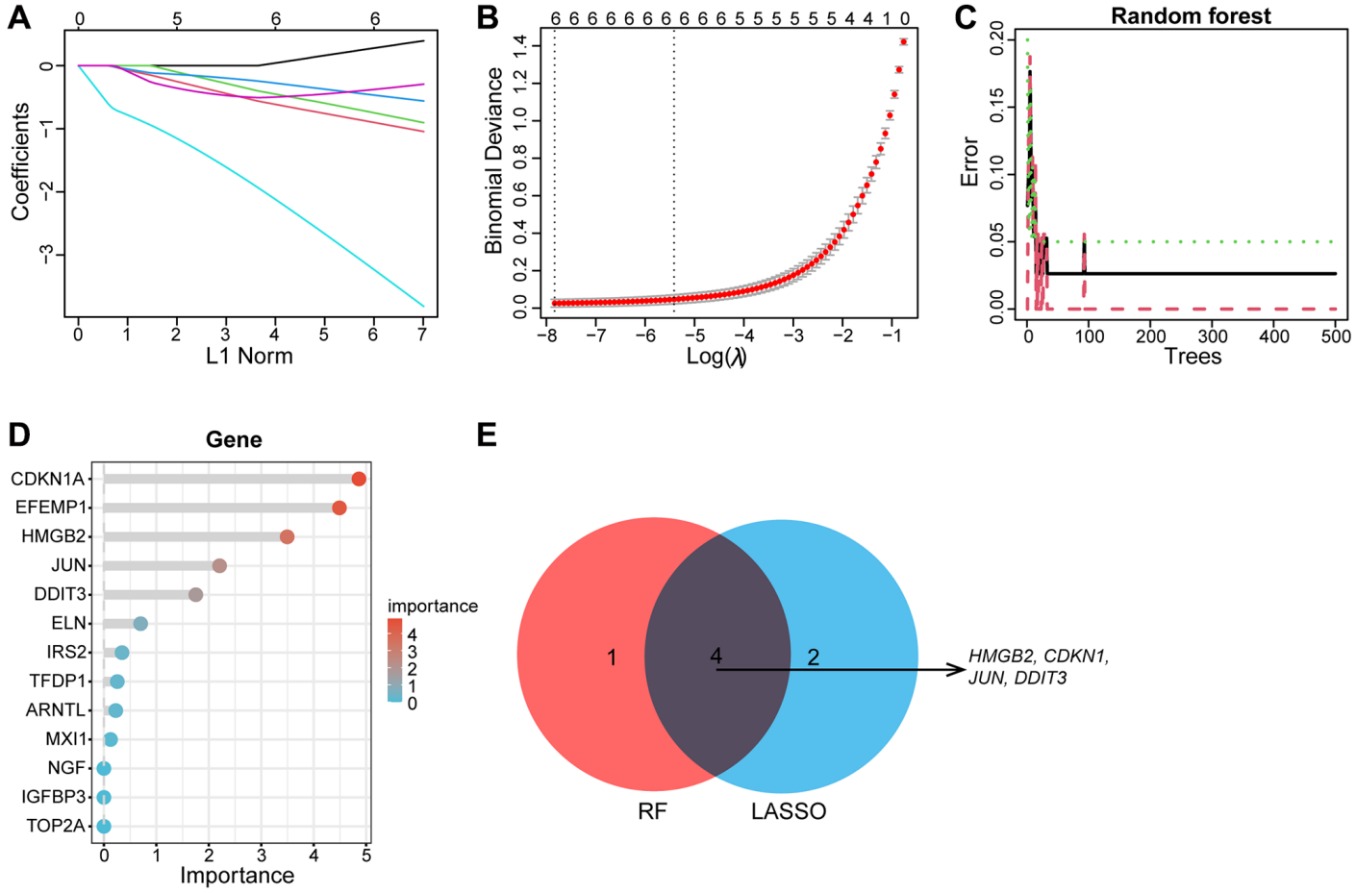
**Machine learning screens biomarkers for diagnosing OA.** (**A**, **B**) The LASSO regression revealed that the number of genes corresponding to the lowest point of the curve (*n* = 6) is best suited for the diagnosis of OA. (**C**, **D**) Random Forest algorithm showed errors in OA; each gene is ranked according to its importance score. (**E**) The Venn diagram depicts the intersection genes of LASSO and RF results.

Subsequently, the LASSO regression and RF machine learning algorithms were further applied to screen for candidate aging-related hub genes, the LASSO regression algorithm identified six candidate genes (Figure 6A, 6B), RF algorithm ranked each gene based on gene importance (Figure 6C, 6D). The intersection of six genes from LASSO and the top five most important genes from the RF was visualized by a Venn diagram. Finally, the four intersectional aging-related biomarker genes we obtained are as follows: *JUN, CDKN1A, DDIT3*, and *HMGB2* (Figure 6E).

### Hub aging-related genes expression levels

**Figure 7:**
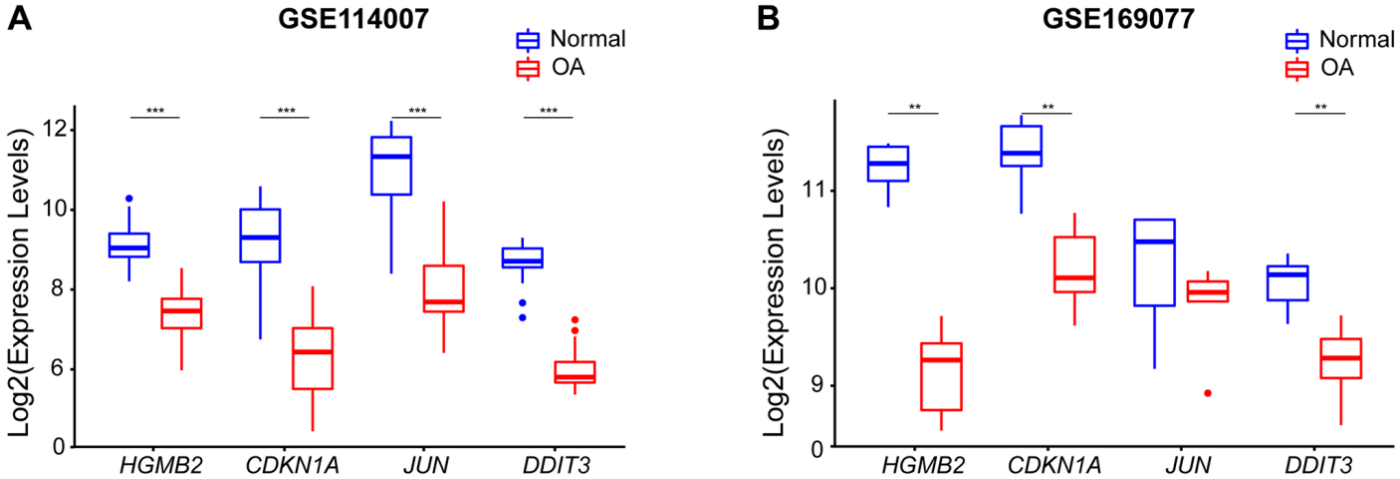
**The expression levels of four hub genes were shown by boxplots.** (**A**) Expression of the hub genes in the GSE114007 dataset, *HMGB2, CDKN1A, JUN*, and*DDIT3* expression were all down-regulated in OA samples. (**B**) The expression of the Hub gene in an external GSE169077 data set (^**^p < 0.01; ^***^p < 0.001).

The expression levels of the four hub genes were validated by using box plots. The results of the training set GSE114007 revealed that the expression levels of *HMGB2*, *CDKN1A*, *JUN*, and *DDIT3* were significantly lower in OA samples than in normal samples (*p* < 0.001) (Figure 7A). To verify the reliability of the result, we used an external data set GSE169077 to further validate the expression levels of the four hub genes (Figure 7B). Consistent with the training set results, the expression levels of *HMGB2, CDKN1A*, and *DDIT3* were significantly lower in OA samples than in normal samples (*p* < 0.01). However, compared with normal samples, although the expression levels of *JUN* were lower in OA, there was no statistical difference (*P* > 0.05) (Figure 7B).

### Hub aging-related genes diagnostic value in OA and normal samples

**Figure 8:**
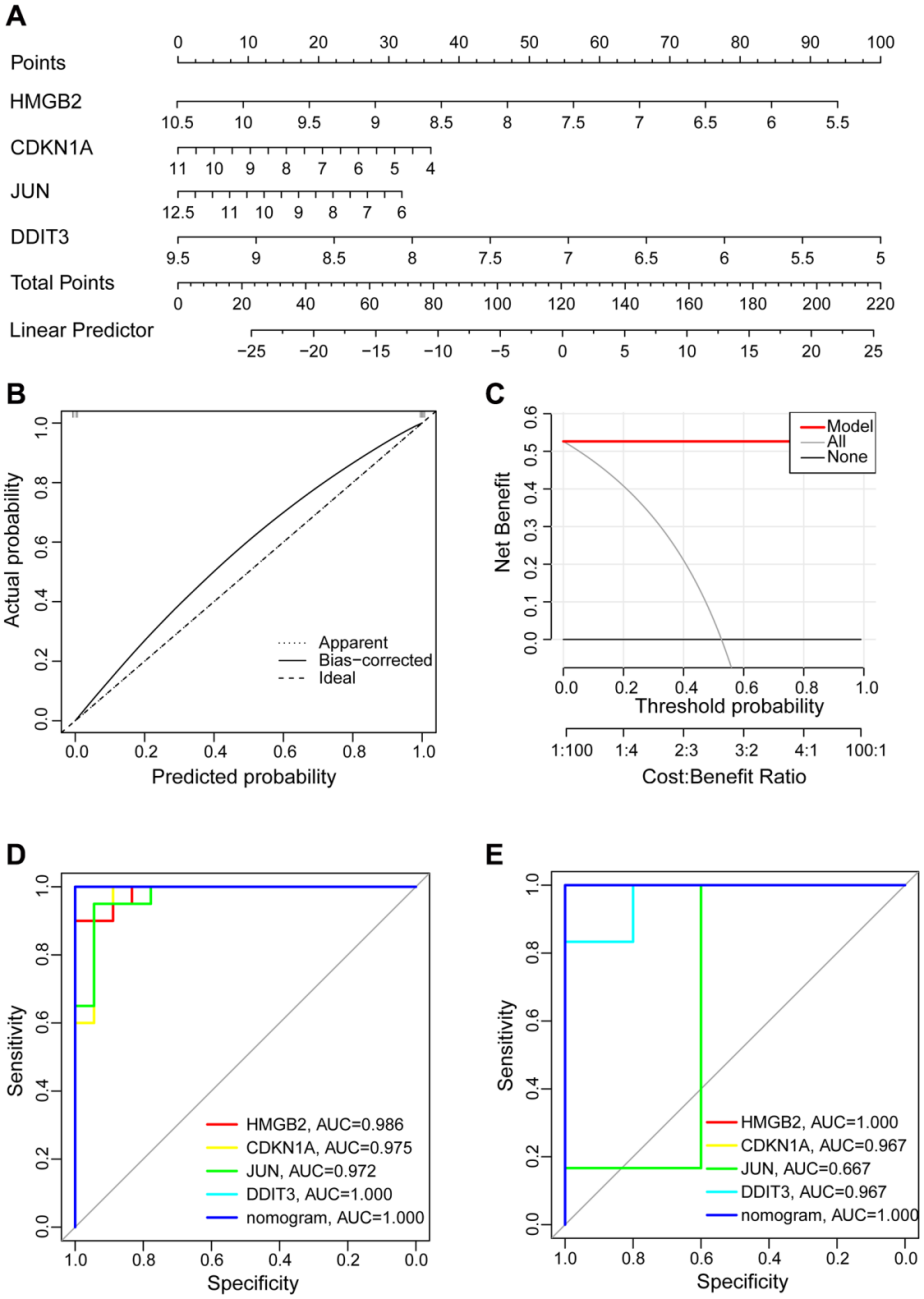
**Construction of the nomogram model and assessment diagnostic value.** (**A**) Construction of the nomogram model on the basis of the four hub aging-related genes. (**B**) The calibration curve evaluates the predictive accuracy of the nomogram model. (**C**) The DCA curve to assess the clinical application value of nomogram model. (**D**) All hub genes and nomogram ROC curve for the training set GSE114007. (**E**) All hub genes and nomogram ROC curve for the validation set GSE169077.

We chose four hub aging-related genes as the final risk prediction model for OA and built the corresponding nomogram to demonstrate the diagnostic value of these hub genes. The nomogram score was used to predict the possibility of suffering from OA (Figure 8A). The calibration curve indicated that nomogram model performed very well in predicting OA (Figure 8B). Also, the DCA indicated the nomogram model has a high clinical application value (Figure 8C). Additionally, ROC curve analysis aims to explore the sensitivity and specificity of nomogram and individual genes in the diagnosis of OA. In the training set GSE114007, the area under the curve (AUC) value of 1.000 for the nomogram was obtained. In the prediction of OA, the AUC values for *HMGB2*, *CDKN1A*, *JUN*, and *DDIT3* were 0.986, 0.975, 0.972, and 1.000, respectively (Figure 8D). In the validation set GSE169077, we obtained similar results indicating that all hub aging-related genes and nomogram have good diagnostic values (Figure 8E).

### RT-PCR validation of the 4 Hub genes

**Figure 9:**
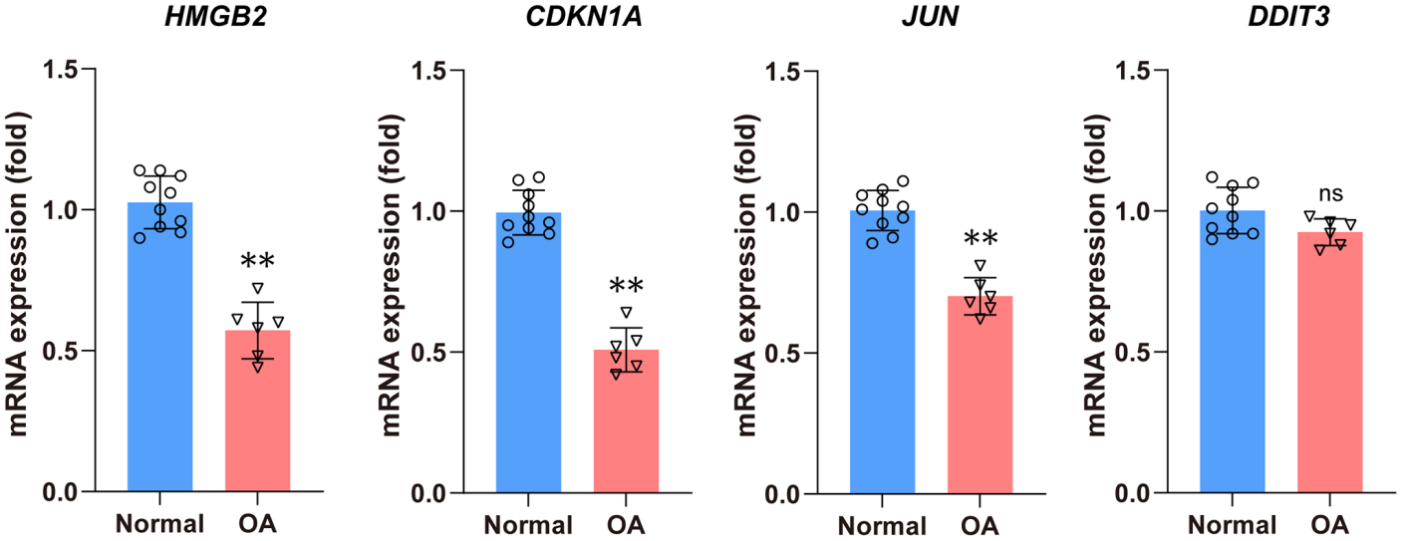
**The qRT-PCR method was used to detect the mRNA expression levels of four hub DEARGs.** Compared with the normal group, the mRNA expression levels of *HMGB2*, *CDKN1A*, and *JUN* were significantly lower in the OA group. There is no statistical difference in *DDIT3* mRNA expression levels between the normal and OA groups.

The results indicated that the relative mRNA expression levels of four hub aging-related genes including *HMGB2*, *CDKN1A*, and *JUN* were consistent with the results of the previous analysis. The *DDIT3* showed no statistically significant difference (Figure 9).

## DISCUSSION

OA may cause by many factors, including mechanical overload, low-grade chronic inflammation, oxidative stress, and cell senescence [5, 10–12]. Cell senescence is an important sign of aging that is characterized by permanent cell cycle arrest and SASP release, destroying the extracellular matrix and affecting cell metabolism, inducing senescence of normal cartilage and synovial cells, and aggravating OA [13–15].

A series of studies on the role of aging-related genes in tumors have been conducted in recent years, but there have been few studies on non-neoplastic aging-related diseases such as idiopathic pulmonary fibrosis, Alzheimer’s disease, atherosclerosis, and so on [16–19]. However, while OA is one of the most common aging-related diseases, it is unclear that the role of aging-related genes in OA progression.

More and more evidence supports that there is a close link between aging and OA. For example, the removal of local senescent cells alleviates the occurrence of traumatic OA and is beneficial to the repair of tissue injury [20]; YAP or FOXD1 reduces cellular senescence in local bone joints and contributes to creating a chondrogenic environment [21]; Up-regulation of MFG-E8 protects against OA by targeting chondrocyte senescence and inhibiting NF-κB pathway [22], and miR-29b-5p alleviates OA via inducing a decline in catabolic enzymes and senescence-related genes and a rising in cartilage ECM synthesis [23]. These findings suggest that senescence plays a vital role in the progression of OA. In this study, we use bioinformatics and machine learning methods to investigate the role of senescence-related genes in OA, as this may provide new ideas for regulating cell senescence and alleviating OA.

We downloaded and analyzed OA patients’ sequencing data from the GEO database. The first 307 aging-related genes were extracted. 42 DEARGs filtered in R using the limma package were crossed with key module genes in WGCNA to yield 13 important genes, and then four hub aging-related genes: *HMGB2, CDKN1A, JUN*, and *DDIT3* were screened using LASSO regression and RF machine learning algorithm. The diagnostic ability of four hub DEARGs to OA was validated using a nomogram and ROC curve based on an external data set. Finally, the qRT-PCR method was used to confirm the credibility of the results.

The high-mobility group box 2 (HMGB2) protein is a chromatin-binding protein that can increase protein binding to chromatin and regulate transcription, DNA damage, and repair [24]. Accumulating research has demonstrated that HMGB2 plays a significant role in controlling cell senescence and aging-related disorders, such as orchestrating the chromatin landscape of SASP gene loci. This suggests that part of *HMGB2*’s function in regulating aging may be through SASP secretion inhibition [25]. *HMGB2* Downregulation Promotes Cellular Senescence in Microvascular Endothelial Cells [26]. In articular cartilage, *HMGB2* is mainly distributed in the superficial zone, the expression of *HMGB2* decreases with aging. OA occurs earlier and more severely in *HMGB2* gene deficiency mice [27]. These reports are consistent with the results of our study.

The *CDKN1A* gene encodes the cyclin-dependent kinase inhibitor p21/WAF1/CIP1/CDKN1A, a mainly transcriptional target of p53. It can inhibit cell cycle progression and cause cell cycle arrest by inactivating cyclin-dependent kinase (CDK) [28, 29]. The p21 also shows a significant expression in normal non-proliferating adult chondrocytes, which indicated it plays an important role in the chondrocyte. Indeed, according to reports, the down-regulation of p21 decreased ACAN expression and increased MMP13 expression through STAT3 phosphorylation in the cartilage tissue. *CDKN1A*-deficient mice are susceptible to inflammation-related OA [30–32]. In this study, compared with the normal group, the expression of p21 was significantly down-regulation in OA. It might well be one reason or at least one mediator of the “de-blocking” of cell cycle progression in OA chondrocytes. In addition, p21 is mainly activated in the early stage of induced senescence, and p16 is necessary to maintain cellular senescence [33–35].

The *JUN* is the transcription factor subunit of activating protein-1 family (AP-1). The exact involvement of AP-1 in osteoarthritis is unknown [36]. Previous research has demonstrated that JUN plays an important function in regulating cell proliferation and apoptosis [37]. *JUN* was able to suppress *p53* gene transcription, and *Jun* knockout mouse embryonic fibroblasts (MEF) increased the expression of p53, resulting in severe proliferation deficiency and early senescence [38, 39]. The Jun NH2-terminal kinase (JNK) pathway may contribute to the regulation of TGF-β-mediated biological responses [40]. Interestingly, recent studies in the intervertebral disc have found that*-Jun* can up-regulate the expression of *TGF-β*, *TIMP-3*, and *COL2A1* mRNA and protein while inhibiting the expression of inflammatory factors, such as IL-1β and TNF-α. Therefore, delaying intervertebral disc degeneration [41]. TGF-β deficiency could be susceptible to osteoarthritis [42]. *JUN* may regulate the development of OA by regulating TGF- β signal transduction. The down-regulation of *JUN* may be an important factor in promoting the development of OA in this study. However, more studies are still needed to fully reveal the roles of *JUN* in OA.

DNA damage-inducible transcript 3(*DDIT3*), also known as C/EBP homologous protein (CHOP) is an endoplasmic reticulum (ER) stress marker [43]. DDIT3/CHOP is activated in response to cellular stressors such as DNA damage, ER stress, cell cycle arrest, and apoptosis [44]. Previous research in mice and ATDC5 chondrocytes has demonstrated that *DDIT3* plays a functional role in chondrocyte metabolism [45]. In the early stages of OA, decreasing ER stress protein (CHOP) production can ameliorate OA caused by ER stress [46]. A recent study, however, has shown that DDIT3/CHOP can promote autophagy in ATDC5 chondrocytes [47]. In the present study, compared to the normal group, *DDIT3* expression was down-regulated in advanced osteoarthritis, we hypothesized that the decreased autophagy end of osteoarthritis could be connected to the downregulation of *DDIT3* expression. This must be demonstrated by further animal experiments.

In general, this study obtained four hub DEARGs via combining bioinformatics analysis and two machine learning algorithms. These genes were shown by the nomogram model to have good diagnostic value for OA, and this was confirmed in another data sets. In addition, qRT-PCR analysis revealed that the expression of four genes was down-regulated in the OA group compared to the normal group, suggesting that these genes may be potential targets for therapeutic intervention.

However, the current study has certain limitations. To begin, our findings are based on public datasets containing a small number of patients. Second, we started with a small number of clinical samples to validate these ARGs identified by the model. More clinical samples, basic experiments, and molecular processes for this signature must be validated in future investigations.

## CONCLUSION

This study identified 4 hub DEARGs (*HMGB2, CDKN1A, JUN*, and *DDIT3*) associated with OA. These genes could be served as potential therapeutic targets for OA. However, more experimental studies are required to confirm its role in OA.

## MATERIALS AND METHODS

### Data download and processing

A total of 307 aging-related genes were downloaded from the Human Ageing Genomic Resources (HAGR) (https://genomics.senescence.info/genes/index.html) [48], the detailed information of genes is listed in Supplementary Table 3. The original sequencing data of GSE114007 were downloaded from the Gene Expression Omnibus (GEO) database, which served as the training dataset. Expression profile data consisted of 18 normal and 20 OA knee cartilage tissue samples. The GSE169077, which as the validation dataset contains 5 normal and 6 OA knee cartilage tissue samples, is based on the GPL96 platform (Affymetrix Human Genome U133A Array). First, the platform annotation information was downloaded to match gene probes to gene names. When numerous probes identified the same gene, the mean expression was determined, and when a gene was expressed in all samples at 0, the gene was eliminated. The data were then normalized using the “quantile normalization” algorithm in the R software “limma” package’s “normalizeBetweenArrays” function.

### Differential expression analysis of aging-related genes

Differentially expressed aging-related genes (DEARGs) were presented between normal and osteoarthritis samples by using the limma software package [49]. The DEARGs satisfied an adjusted *P* value < 0.05 and |log2-fold-change| > 1. The “heatmap” and “ggplot2” software packages of R were used to draw heat maps and volcano maps.

### Gene ontology, pathway enrichment and PPI network analysis of DEARGs

We performed GO functional enrichment and KEGG pathway enrichment analysis on DEARGs using the “Clusterprofiler” R package [50–52]. The STRING database (https://string-db.org/) was used to observe the PPI network between the DEARGs [53].

### Weighted gene co-expression network analysis (WGCNA)

Weighted gene co-expression network analysis (WGCNA) is an algorithm that may identify co-expressed gene modules of significant biological value and investigate the association between gene networks and diseases. The dataset was utilized for weighted co-expression network construction using the “WGCNA” package for R to choose the best soft threshold (β) using the “pickSoftThreshold” function. The matrix data were converted into an adjacency matrix, which was then transformed into a topological overlap matrix (TOM) and the corresponding dissimilarity (1-TOM), which were separated into different modules based on similarly expressed genes and represented by different colors. The expression profile of each module was represented by a module eigengene (ME), and the correlation between each ME and clinical characteristics was calculated [54].

### Identification of potential biomarkers in normal and OA

Two machine learning algorithms were used to further screen candidate genes for OA diagnosis. The DEARGs and genes from key modules were intersected, and then the least absolute shrinkage and selection operator (LASSO) and Random Forest (RF) were employed to screen the diagnostic genes. The “glmnet” package in R was used to perform LASSO, a regression analysis algorithm that applies regularization to variable selection [55, 56]. RF is a popular machine learning algorithm that is widely used in bioinformatics analysis to screen important genes, and it can be accomplished by using the “randomForest” package in R software [57]. Overlapping genes resulting from two algorithms as hub aging-related genes in OA diagnosis.

### Identification of Hub aging-related genes expression levels and diagnostic model construction

Hub gene expression levels in healthy and OA individuals were assessed with the help of box plots. Hub aging-related genes were incorporated to construct a nomogram as a diagnostic model via using the “rms” package in R software, nomogram construction is critical for clinical OA diagnosis [58]. The predictive ability and clinical practicability of the nomogram model were evaluated by calibration curve and decision curve analysis (DCA), respectively [59]. Receiver operating characteristic (ROC) curves were plotted using the “pROC” packages of R to assess the levels of hub aging-related genes distinguishing between healthy and OA individuals, Furthermore, the expression levels and diagnostic value of the hub aging-related genes were validated with a separate external data set GSE169077.

### Patients’ samples

The human cartilage samples were obtained from ten OA patients who underwent total knee arthroplasty and the normal cartilage samples were collected from six patients with anterior cruciate ligament rupture. All patients signed informed consent and samples were collected, processed, and analyzed under the guidance of the ethics committee of the Guangzhou Red Cross Hospital of Jinan University (Ethics number 2018–292).

### qRT-PCR

The hub DEARGs were validated using quantitative real-time PCR. Total RNA was reverse-transcribed to cDNA according to the manufacturer’s instructions using the PrimeScript RT reagent Kit (TaKaRa, Japan). *GAPDH* was used as the housekeeping gene and relative mRNA expression levels were determined using the 2^−ΔΔCt^ comparative method from the mean of triplicate treatments averaged from 3 replicate PCR reactions. The primer sequences are as follows. *GAPDH* Forward: 5′-ACACCCACTCCTCCACCTTT-3′; Reverse: 5′-TTACTACTTGGAGGCCATGT-3′; *HMGB2* Forward: 5′-CCGGACTCTTCCGTCAATTTC-3′; Reverse: 5′-GTCATAGCGAGCTTTGTCACT-3′; *CDKN1A* Forward: 5′-GGCATTCTGGGAGCTTCATCT-3′; Reverse: 5′-AGGGTGCCCTTCTTCTTGTG-3′; *JUN* Forward: 5′-TCCAAGTGCCGAAAAAGGAAG-3′; Reverse: 5′-CGAGTTCTGAGCTTTCAAGGT-3′; *DDIT3*, Forward strand: 5′-GGAAACAGAGTGGTCATTCCC-3′; Reverse: 5′-CTGCTTGAGCCGTTCATTCTC-3′.

### Statistical analysis

R project (version 4.1.0) was used for our data processing and analysis. The data of qRT-PCR were analyzed using the SPSS 22 (IBM SPSS Statistics, USA). Differences between experimental and control groups were calculated using an unpair *t*-test as the statistical method. *p* value < 0.05 was considered as statistically significant. Data were presented as mean ± standard deviation (SD).

### Data availability

All data, models, and code developed or utilized during the study are available from the corresponding author upon reasonable request.

## Supplementary Materials

Supplementary Table 1

Supplementary Table 2

Supplementary Table 3

## References

[1] GBD 2017 Disease and Injury Incidence and Prevalence Collaborators. Global, regional, and national incidence, prevalence, and years lived with disability for 354 diseases and injuries for 195 countries and territories, 1990-2017: a systematic analysis for the Global Burden of Disease Study 2017. Lancet. 2018; 392:1789–858. 10.1016/S0140-6736(18)32279-7 30496104 PMC6227754

[2] Hunter DJ, Bierma-Zeinstra S. Osteoarthritis. Lancet. 2019; 393:1745–59. 10.1016/S0140-6736(19)30417-9 31034380

[3] Glyn-Jones S, Palmer AJ, Agricola R, Price AJ, Vincent TL, Weinans H, Carr AJ. Osteoarthritis. Lancet. 2015; 386:376–87. 10.1016/S0140-6736(14)60802-3 25748615

[4] McCulloch K, Litherland GJ, Rai TS. Cellular senescence in osteoarthritis pathology. Aging Cell. 2017; 16:210–8. 10.1111/acel.12562 28124466 PMC5334539

[5] Xie J, Wang Y, Lu L, Liu L, Yu X, Pei F. Cellular senescence in knee osteoarthritis: molecular mechanisms and therapeutic implications. Ageing Res Rev. 2021; 70:101413. 10.1016/j.arr.2021.101413 34298194

[6] Xu M, Pirtskhalava T, Farr JN, Weigand BM, Palmer AK, Weivoda MM, Inman CL, Ogrodnik MB, Hachfeld CM, Fraser DG, Onken JL, Johnson KO, Verzosa GC, et al. Senolytics improve physical function and increase lifespan in old age. Nat Med. 2018; 24:1246–56. 10.1038/s41591-018-0092-9 29988130 PMC6082705

[7] Martel J, Ojcius DM, Wu CY, Peng HH, Voisin L, Perfettini JL, Ko YF, Young JD. Emerging use of senolytics and senomorphics against aging and chronic diseases. Med Res Rev. 2020; 40:2114–31. 10.1002/med.21702 32578904

[8] Coryell PR, Diekman BO, Loeser RF. Mechanisms and therapeutic implications of cellular senescence in osteoarthritis. Nat Rev Rheumatol. 2021; 17:47–57. 10.1038/s41584-020-00533-7 33208917 PMC8035495

[9] Orlov YL, Anashkina AA, Klimontov VV, Baranova AV. Medical Genetics, Genomics and Bioinformatics Aid in Understanding Molecular Mechanisms of Human Diseases. Int J Mol Sci. 2021; 22:9962. 10.3390/ijms22189962 34576125 PMC8467458

[10] Hodgkinson T, Kelly DC, Curtin CM, O'Brien FJ. Mechanosignalling in cartilage: an emerging target for the treatment of osteoarthritis. Nat Rev Rheumatol. 2022; 18:67–84. 10.1038/s41584-021-00724-w 34934171

[11] Robinson WH, Lepus CM, Wang Q, Raghu H, Mao R, Lindstrom TM, Sokolove J. Low-grade inflammation as a key mediator of the pathogenesis of osteoarthritis. Nat Rev Rheumatol. 2016; 12:580–92. 10.1038/nrrheum.2016.136 27539668 PMC5500215

[12] Xie J, Lin J, Wei M, Teng Y, He Q, Yang G, Yang X. Sustained Akt signaling in articular chondrocytes causes osteoarthritis via oxidative stress-induced senescence in mice. Bone Res. 2019; 7:23. 10.1038/s41413-019-0062-y 31646013 PMC6804644

[13] López-Otín C, Blasco MA, Partridge L, Serrano M, Kroemer G. The hallmarks of aging. Cell. 2013; 153:1194–217. 10.1016/j.cell.2013.05.039 23746838 PMC3836174

[14] Kang D, Shin J, Cho Y, Kim HS, Gu YR, Kim H, You KT, Chang MJ, Chang CB, Kang SB, Kim JS, Kim VN, Kim JH. Stress-activated miR-204 governs senescent phenotypes of chondrocytes to promote osteoarthritis development. Sci Transl Med. 2019; 11:eaar6659. 10.1126/scitranslmed.aar6659 30944169

[15] Chen X, Gong W, Shao X, Shi T, Zhang L, Dong J, Shi Y, Shen S, Qin J, Jiang Q, Guo B. METTL3-mediated m^6^A modification of ATG7 regulates autophagy-GATA4 axis to promote cellular senescence and osteoarthritis progression. Ann Rheum Dis. 2022; 81:87–99. 10.1136/annrheumdis-2021-221091 34706873

[16] Luo C, Nie H, Yu L. Identification of Aging-Related Genes Associated with Prognostic Value and Immune Microenvironment Characteristics in Diffuse Large B-Cell Lymphoma. Oxid Med Cell Longev. 2022; 2022:3334522. 10.1155/2022/3334522 35069971 PMC8777392

[17] He J, Li X. Identification and Validation of Aging-Related Genes in Idiopathic Pulmonary Fibrosis. Front Genet. 2022; 13:780010. 10.3389/fgene.2022.780010 35211155 PMC8863089

[18] Zhang Q, Li J, Weng L. Identification and Validation of Aging-Related Genes in Alzheimer's Disease. Front Neurosci. 2022; 16:905722. 10.3389/fnins.2022.905722 35615282 PMC9124812

[19] Zhao L, Lv F, Zheng Y, Yan L, Cao X. Characterization of an Aging-Based Diagnostic Gene Signature and Molecular Subtypes With Diverse Immune Infiltrations in Atherosclerosis. Front Mol Biosci. 2022; 8:792540. 10.3389/fmolb.2021.792540 35096968 PMC8792769

[20] Jeon OH, Kim C, Laberge RM, Demaria M, Rathod S, Vasserot AP, Chung JW, Kim DH, Poon Y, David N, Baker DJ, van Deursen JM, Campisi J, Elisseeff JH. Local clearance of senescent cells attenuates the development of post-traumatic osteoarthritis and creates a pro-regenerative environment. Nat Med. 2017; 23:775–81. 10.1038/nm.4324 28436958 PMC5785239

[21] Fu L, Hu Y, Song M, Liu Z, Zhang W, Yu FX, Wu J, Wang S, Izpisua Belmonte JC, Chan P, Qu J, Tang F, Liu GH. Up-regulation of FOXD1 by YAP alleviates senescence and osteoarthritis. PLoS Biol. 2019; 17:e3000201. 10.1371/journal.pbio.3000201 30933975 PMC6459557

[22] Lu Y, Liu L, Pan J, Luo B, Zeng H, Shao Y, Zhang H, Guan H, Guo D, Zeng C, Zhang R, Bai X, Zhang H, Cai D. MFG-E8 regulated by miR-99b-5p protects against osteoarthritis by targeting chondrocyte senescence and macrophage reprogramming via the NF-κB pathway. Cell Death Dis. 2021; 12:533. 10.1038/s41419-021-03800-x 34031369 PMC8144578

[23] Zhu J, Yang S, Qi Y, Gong Z, Zhang H, Liang K, Shen P, Huang YY, Zhang Z, Ye W, Yue L, Fan S, Shen S, et al. Stem cell-homing hydrogel-based miR-29b-5p delivery promotes cartilage regeneration by suppressing senescence in an osteoarthritis rat model. Sci Adv. 2022; 8:eabk0011. 10.1126/sciadv.abk0011 35353555 PMC8967232

[24] Voong CK, Goodrich JA, Kugel JF. Interactions of HMGB Proteins with the Genome and the Impact on Disease. Biomolecules. 2021; 11:1451. 10.3390/biom11101451 34680084 PMC8533419

[25] Aird KM, Iwasaki O, Kossenkov AV, Tanizawa H, Fatkhutdinov N, Bitler BG, Le L, Alicea G, Yang TL, Johnson FB, Noma KI, Zhang R. HMGB2 orchestrates the chromatin landscape of senescence-associated secretory phenotype gene loci. J Cell Biol. 2016; 215:325–34. 10.1083/jcb.201608026 27799366 PMC5100296

[26] Jo HR, Jeong JH. MicroRNA-Mediated Downregulation of HMGB2 Contributes to Cellular Senescence in Microvascular Endothelial Cells. Cells. 2022; 11:584. 10.3390/cells11030584 35159393 PMC8834370

[27] Taniguchi N, Caramés B, Ronfani L, Ulmer U, Komiya S, Bianchi ME, Lotz M. Aging-related loss of the chromatin protein HMGB2 in articular cartilage is linked to reduced cellularity and osteoarthritis. Proc Natl Acad Sci U S A. 2009; 106:1181–6. 10.1073/pnas.0806062106 19139395 PMC2633567

[28] Engeland K. Cell cycle regulation: p53-p21-RB signaling. Cell Death Differ. 2022; 29:946–60. 10.1038/s41418-022-00988-z 35361964 PMC9090780

[29] Harper JW, Adami GR, Wei N, Keyomarsi K, Elledge SJ. The p21 Cdk-interacting protein Cip1 is a potent inhibitor of G1 cyclin-dependent kinases. Cell. 1993; 75:805–16. 10.1016/0092-8674(93)90499-g 8242751

[30] Hayashi S, Fujishiro T, Hashimoto S, Kanzaki N, Chinzei N, Kihara S, Takayama K, Matsumoto T, Nishida K, Kurosaka M, Kuroda R. p21 deficiency is susceptible to osteoarthritis through STAT3 phosphorylation. Arthritis Res Ther. 2015; 17:314. 10.1186/s13075-015-0828-6 26546411 PMC4636813

[31] Kihara S, Hayashi S, Hashimoto S, Kanzaki N, Takayama K, Matsumoto T, Chinzei N, Iwasa K, Haneda M, Takeuchi K, Nishida K, Kuroda R. Cyclin-Dependent Kinase Inhibitor-1-Deficient Mice are Susceptible to Osteoarthritis Associated with Enhanced Inflammation. J Bone Miner Res. 2017; 32:991–1001. 10.1002/jbmr.3080 28128866

[32] Takashima Y, Hayashi S, Fukuda K, Maeda T, Tsubosaka M, Kamenaga T, Kikuchi K, Fujita M, Kuroda Y, Hashimoto S, Nakano N, Matsumoto T, Kuroda R. Susceptibility of cyclin-dependent kinase inhibitor 1-deficient mice to rheumatoid arthritis arising from interleukin-1β-induced inflammation. Sci Rep. 2021; 11:12516. 10.1038/s41598-021-92055-9 34131243 PMC8206139

[33] Sesselmann S, Söder S, Voigt R, Haag J, Grogan SP, Aigner T. DNA methylation is not responsible for p21WAF1/CIP1 down-regulation in osteoarthritic chondrocytes. Osteoarthritis Cartilage. 2009; 17:507–12. 10.1016/j.joca.2008.09.006 18954998

[34] Kumari R, Jat P. Mechanisms of Cellular Senescence: Cell Cycle Arrest and Senescence Associated Secretory Phenotype. Front Cell Dev Biol. 2021; 9:645593. 10.3389/fcell.2021.645593 33855023 PMC8039141

[35] Huang W, Hickson LJ, Eirin A, Kirkland JL, Lerman LO. Cellular senescence: the good, the bad and the unknown. Nat Rev Nephrol. 2022; 18:611–27. 10.1038/s41581-022-00601-z 35922662 PMC9362342

[36] Karin M, Liu ZG, Zandi E. AP-1 function and regulation. Curr Opin Cell Biol. 1997; 9:240–6. 10.1016/s0955-0674(97)80068-3 9069263

[37] Kappelmann M, Bosserhoff A, Kuphal S. AP-1/c-Jun transcription factors: regulation and function in malignant melanoma. Eur J Cell Biol. 2014; 93:76–81. 10.1016/j.ejcb.2013.10.003 24315690

[38] Schreiber M, Kolbus A, Piu F, Szabowski A, Möhle-Steinlein U, Tian J, Karin M, Angel P, Wagner EF. Control of cell cycle progression by c-Jun is p53 dependent. Genes Dev. 1999; 13:607–19. 10.1101/gad.13.5.607 10072388 PMC316508

[39] Meixner A, Karreth F, Kenner L, Penninger JM, Wagner EF. Jun and JunD-dependent functions in cell proliferation and stress response. Cell Death Differ. 2010; 17:1409–19. 10.1038/cdd.2010.22 20300111

[40] Ventura JJ, Kennedy NJ, Flavell RA, Davis RJ. JNK regulates autocrine expression of TGF-beta1. Mol Cell. 2004; 15:269–78. 10.1016/j.molcel.2004.06.007 15260977

[41] Lei M, Zhao K, Hua W, Wang K, Li S, Wu X, Yang C. An in vivo study of the effect of c-Jun on intervertebral disc degeneration in rats. Bioengineered. 2021; 12:4320–30. 10.1080/21655979.2021.1946459 34308759 PMC8806816

[42] Wang C, Shen J, Ying J, Xiao D, O'Keefe RJ. FoxO1 is a crucial mediator of TGF-β/TAK1 signaling and protects against osteoarthritis by maintaining articular cartilage homeostasis. Proc Natl Acad Sci U S A. 2020; 117:30488–97. 10.1073/pnas.2017056117 33199631 PMC7720227

[43] Woehlbier U, Hetz C. Modulating stress responses by the UPRosome: a matter of life and death. Trends Biochem Sci. 2011; 36:329–37. 10.1016/j.tibs.2011.03.001 21482118

[44] Hu H, Tian M, Ding C, Yu S. The C/EBP Homologous Protein (CHOP) Transcription Factor Functions in Endoplasmic Reticulum Stress-Induced Apoptosis and Microbial Infection. Front Immunol. 2019; 9:3083. 10.3389/fimmu.2018.03083 30662442 PMC6328441

[45] Yu M, Yi SQ, Wu YR, Sun HL, Song FF, Wang JW. Ddit3 suppresses the differentiation of mouse chondroprogenitor cells. Int J Biochem Cell Biol. 2016; 81:156–63. 10.1016/j.biocel.2016.11.009 27845261

[46] Hecht JT, Veerisetty AC, Wu J, Coustry F, Hossain MG, Chiu F, Gannon FH, Posey KL. Primary Osteoarthritis Early Joint Degeneration Induced by Endoplasmic Reticulum Stress Is Mitigated by Resveratrol. Am J Pathol. 2021; 191:1624–37. 10.1016/j.ajpath.2021.05.016 34116024 PMC8420863

[47] Yang C, Xu X, Dong X, Yang B, Dong W, Luo Y, Liu X, Wu Y, Wang J. DDIT3/CHOP promotes autophagy in chondrocytes via SIRT1-AKT pathway. Biochim Biophys Acta Mol Cell Res. 2021; 1868:119074. 10.1016/j.bbamcr.2021.119074 34087318

[48] Tacutu R, Thornton D, Johnson E, Budovsky A, Barardo D, Craig T, Diana E, Lehmann G, Toren D, Wang J, Fraifeld VE, de Magalhães JP. Human Ageing Genomic Resources: new and updated databases. Nucleic Acids Res. 2018; 46:D1083–90. 10.1093/nar/gkx1042 29121237 PMC5753192

[49] Ritchie ME, Phipson B, Wu D, Hu Y, Law CW, Shi W, Smyth GK. limma powers differential expression analyses for RNA-sequencing and microarray studies. Nucleic Acids Res. 2015; 43:e47. 10.1093/nar/gkv007 25605792 PMC4402510

[50] The Gene Ontology Consortium. The Gene Ontology Resource: 20 years and still GOing strong. Nucleic Acids Res. 2019; 47:D330–8. 10.1093/nar/gky1055 30395331 PMC6323945

[51] Kanehisa M, Goto S. KEGG: kyoto encyclopedia of genes and genomes. Nucleic Acids Res. 2000; 28:27–30. 10.1093/nar/28.1.27 10592173 PMC102409

[52] Yu G, Wang LG, Han Y, He QY. clusterProfiler: an R package for comparing biological themes among gene clusters. OMICS. 2012; 16:284–7. 10.1089/omi.2011.0118 22455463 PMC3339379

[53] Szklarczyk D, Gable AL, Nastou KC, Lyon D, Kirsch R, Pyysalo S, Doncheva NT, Legeay M, Fang T, Bork P, Jensen LJ, von Mering C. The STRING database in 2021: customizable protein-protein networks, and functional characterization of user-uploaded gene/measurement sets. Nucleic Acids Res. 2021; 49:D605–12. 10.1093/nar/gkaa1074 33237311 PMC7779004

[54] Langfelder P, Horvath S. WGCNA: an R package for weighted correlation network analysis. BMC Bioinformatics. 2008; 9:559. 10.1186/1471-2105-9-559 19114008 PMC2631488

[55] Cai W, van der Laan M. Nonparametric bootstrap inference for the targeted highly adaptive least absolute shrinkage and selection operator (LASSO) estimator. Int J Biostat. 2020. [Epub ahead of print]. 10.1515/ijb-2017-0070 32772002

[56] Tibshirani R. The lasso method for variable selection in the Cox model. Stat Med. 1997; 16:385–95. 10.1002/(sici)1097-0258(19970228)16:4<385::aid-sim380>3.0.co;2-3 9044528

[57] Sun D, Peng H, Wu Z. Establishment and Analysis of a Combined Diagnostic Model of Alzheimer's Disease With Random Forest and Artificial Neural Network. Front Aging Neurosci. 2022; 14:921906. 10.3389/fnagi.2022.921906 35847663 PMC9280980

[58] Deng M, Yin Y, Zhang Q, Zhou X, Hou G. Identification of Inflammation-Related Biomarker Lp-PLA2 for Patients With COPD by Comprehensive Analysis. Front Immunol. 2021; 12:670971. 10.3389/fimmu.2021.670971 34093570 PMC8176901

[59] Balachandran VP, Gonen M, Smith JJ, DeMatteo RP. Nomograms in oncology: more than meets the eye. Lancet Oncol. 2015; 16:e173–80. 10.1016/S1470-2045(14)71116-7 25846097 PMC4465353

