# Supplementary material for: Identification of aging-related genes in diagnosing osteoarthritis via integrating bioinformatics analysis and machine learning
Source: Aging (Albany NY). 2024 Jan 3;16(1):153–68. doi: 10.18632/aging.205357 (PMC10817387; doi:10.18632/aging.205357)
Supplement: Supplementary Table 1 [file aging-16-205357-s001.docx]

| **Supplementary Table 1. Results of GO function enrichment analysis.** | | | | | | | | | |
| --- | --- | --- | --- | --- | --- | --- | --- | --- | --- |
| ONTOLOGY | ID | Description | GeneRatio | BgRatio | pvalue | p.adjust | qvalue | geneID | Count |
| BP | GO:0007568 | aging | 15/41 | 339/18723 | 2.24E-16 | 4.49E-13 | 2.54E-13 | JUN/CDKN1A/ARNTL/FOS/PCK1/CDK1/NFKB2/RELA/UCP2/CDKN2B/C1QA/PTGS2/JUND/UCP3/SERPINE1 | 15 |
| BP | GO:0032496 | response to lipopolysaccharide | 11/41 | 343/18723 | 1.29E-10 | 1.29E-07 | 7.28E-08 | JUN/HMGB2/CEBPB/FOS/PCK1/NFKB2/RELA/PTGS2/JUND/IL6/SERPINE1 | 11 |
| BP | GO:0002237 | response to molecule of bacterial origin | 11/41 | 363/18723 | 2.35E-10 | 1.38E-07 | 7.80E-08 | JUN/HMGB2/CEBPB/FOS/PCK1/NFKB2/RELA/PTGS2/JUND/IL6/SERPINE1 | 11 |
| BP | GO:0031100 | animal organ regeneration | 7/41 | 76/18723 | 2.75E-10 | 1.38E-07 | 7.80E-08 | CDKN1A/CEBPB/CCNA2/PCNA/CDK1/UCP2/IL6 | 7 |
| BP | GO:0000302 | response to reactive oxygen species | 9/41 | 222/18723 | 9.93E-10 | 3.98E-07 | 2.25E-07 | JUN/PRDX1/FOS/PCNA/CDK1/RELA/UCP2/UCP3/IL6 | 9 |
| BP | GO:0043620 | regulation of DNA-templated transcription in response to stress | 6/41 | 53/18723 | 1.60E-09 | 5.03E-07 | 2.84E-07 | DDIT3/JUN/VEGFA/CEBPB/EGR1/RELA | 6 |
| BP | GO:0006979 | response to oxidative stress | 11/41 | 446/18723 | 2.06E-09 | 5.03E-07 | 2.84E-07 | JUN/PRDX1/ARNTL/FOS/PCNA/CDK1/RELA/UCP2/PTGS2/UCP3/IL6 | 11 |
| BP | GO:0062197 | cellular response to chemical stress | 10/41 | 337/18723 | 2.13E-09 | 5.03E-07 | 2.84E-07 | DDIT3/JUN/PRDX1/ARNTL/FOS/PCNA/CDK1/RELA/PTGS2/IL6 | 10 |
| BP | GO:0048545 | response to steroid hormone | 10/41 | 339/18723 | 2.26E-09 | 5.03E-07 | 2.84E-07 | CDKN1A/HMGB2/ARNTL/FOS/PCK1/PCNA/RELA/PTGS2/UCP3/IL6 | 10 |
| BP | GO:0070482 | response to oxygen levels | 10/41 | 347/18723 | 2.83E-09 | 5.66E-07 | 3.20E-07 | CDKN1A/VEGFA/CCNA2/PCK1/EGR1/UCP2/MYC/PTGS2/UCP3/PLAU | 10 |
| BP | GO:0048511 | rhythmic process | 9/41 | 298/18723 | 1.30E-08 | 2.37E-06 | 1.34E-06 | JUN/TOP2A/ARNTL/EGR1/PCNA/CDK1/NFKB2/JUND/NGFR | 9 |
| BP | GO:0001666 | response to hypoxia | 9/41 | 307/18723 | 1.68E-08 | 2.81E-06 | 1.59E-06 | VEGFA/CCNA2/PCK1/EGR1/UCP2/MYC/PTGS2/UCP3/PLAU | 9 |
| BP | GO:0036293 | response to decreased oxygen levels | 9/41 | 322/18723 | 2.54E-08 | 3.88E-06 | 2.19E-06 | VEGFA/CCNA2/PCK1/EGR1/UCP2/MYC/PTGS2/UCP3/PLAU | 9 |
| BP | GO:0001889 | liver development | 7/41 | 147/18723 | 2.86E-08 | 3.88E-06 | 2.19E-06 | CEBPB/PCK1/PCNA/RELA/UCP2/CEBPA/IL6 | 7 |
| BP | GO:0051384 | response to glucocorticoid | 7/41 | 148/18723 | 3.00E-08 | 3.88E-06 | 2.19E-06 | CDKN1A/FOS/PCK1/PCNA/PTGS2/UCP3/IL6 | 7 |
| BP | GO:1902895 | positive regulation of pri-miRNA transcription by RNA polymerase II | 5/41 | 42/18723 | 3.13E-08 | 3.88E-06 | 2.19E-06 | JUN/FOS/EGR1/RELA/NGFR | 5 |
| BP | GO:0061008 | hepaticobiliary system development | 7/41 | 150/18723 | 3.29E-08 | 3.88E-06 | 2.19E-06 | CEBPB/PCK1/PCNA/RELA/UCP2/CEBPA/IL6 | 7 |
| BP | GO:0034614 | cellular response to reactive oxygen species | 7/41 | 155/18723 | 4.13E-08 | 4.60E-06 | 2.60E-06 | JUN/PRDX1/FOS/PCNA/CDK1/RELA/IL6 | 7 |
| BP | GO:0031667 | response to nutrient levels | 10/41 | 474/18723 | 5.46E-08 | 5.62E-06 | 3.18E-06 | DDIT3/JUN/CDKN1A/PCK1/RELA/UCP2/HSPA8/CDKN2B/PTGS2/UCP3 | 10 |
| BP | GO:0043618 | regulation of transcription from RNA polymerase II promoter in response to stress | 5/41 | 47/18723 | 5.61E-08 | 5.62E-06 | 3.18E-06 | DDIT3/JUN/VEGFA/CEBPB/EGR1 | 5 |
| BP | GO:0031960 | response to corticosteroid | 7/41 | 167/18723 | 6.90E-08 | 6.58E-06 | 3.72E-06 | CDKN1A/FOS/PCK1/PCNA/PTGS2/UCP3/IL6 | 7 |
| BP | GO:0001659 | temperature homeostasis | 7/41 | 174/18723 | 9.14E-08 | 8.32E-06 | 4.70E-06 | DDIT3/VEGFA/CEBPB/ARNTL/EGR1/UCP2/PTGS2 | 7 |
| BP | GO:1902893 | regulation of pri-miRNA transcription by RNA polymerase II | 5/41 | 54/18723 | 1.14E-07 | 9.96E-06 | 5.63E-06 | JUN/FOS/EGR1/RELA/NGFR | 5 |
| BP | GO:0061614 | pri-miRNA transcription by RNA polymerase II | 5/41 | 55/18723 | 1.26E-07 | 1.05E-05 | 5.93E-06 | JUN/FOS/EGR1/RELA/NGFR | 5 |
| BP | GO:0034599 | cellular response to oxidative stress | 8/41 | 288/18723 | 1.75E-07 | 1.40E-05 | 7.93E-06 | JUN/PRDX1/ARNTL/FOS/PCNA/CDK1/RELA/IL6 | 8 |
| BP | GO:0043434 | response to peptide hormone | 9/41 | 414/18723 | 2.17E-07 | 1.64E-05 | 9.25E-06 | IRS2/CCNA2/PCK1/EGR1/RELA/UCP2/PTGS2/JUND/UCP3 | 9 |
| BP | GO:0031099 | regeneration | 7/41 | 198/18723 | 2.21E-07 | 1.64E-05 | 9.25E-06 | CDKN1A/CEBPB/CCNA2/PCNA/CDK1/UCP2/IL6 | 7 |
| BP | GO:0007623 | circadian rhythm | 7/41 | 210/18723 | 3.29E-07 | 2.32E-05 | 1.31E-05 | JUN/TOP2A/ARNTL/EGR1/CDK1/JUND/NGFR | 7 |
| BP | GO:0048732 | gland development | 9/41 | 436/18723 | 3.35E-07 | 2.32E-05 | 1.31E-05 | IRS2/VEGFA/CEBPB/PCK1/PCNA/RELA/UCP2/CEBPA/IL6 | 9 |
| BP | GO:0071496 | cellular response to external stimulus | 8/41 | 320/18723 | 3.90E-07 | 2.61E-05 | 1.47E-05 | JUN/CDKN1A/FOS/PCK1/UCP2/HSPA8/CDKN2B/PTGS2 | 8 |
| BP | GO:0009314 | response to radiation | 9/41 | 456/18723 | 4.89E-07 | 3.16E-05 | 1.79E-05 | JUN/CDKN1A/FOS/EGR1/PCNA/RELA/MYC/PTGS2/JUND | 9 |
| BP | GO:0009410 | response to xenobiotic stimulus | 9/41 | 462/18723 | 5.45E-07 | 3.31E-05 | 1.87E-05 | JUN/CDKN1A/FOS/PCNA/CDK1/RELA/MYC/PTGS2/UCHL1 | 9 |
| BP | GO:0045598 | regulation of fat cell differentiation | 6/41 | 139/18723 | 5.46E-07 | 3.31E-05 | 1.87E-05 | DDIT3/CEBPB/ARNTL/CEBPA/PTGS2/IL6 | 6 |
| BP | GO:0071456 | cellular response to hypoxia | 6/41 | 151/18723 | 8.87E-07 | 5.23E-05 | 2.96E-05 | VEGFA/CCNA2/PCK1/EGR1/MYC/PTGS2 | 6 |
| BP | GO:0031668 | cellular response to extracellular stimulus | 7/41 | 246/18723 | 9.53E-07 | 5.31E-05 | 3.00E-05 | JUN/CDKN1A/FOS/PCK1/UCP2/HSPA8/CDKN2B | 7 |
| BP | GO:0070997 | neuron death | 8/41 | 361/18723 | 9.69E-07 | 5.31E-05 | 3.00E-05 | DDIT3/JUN/CEBPB/FOS/EGR1/NGF/C1QA/NGFR | 8 |
| BP | GO:0097421 | liver regeneration | 4/41 | 35/18723 | 9.86E-07 | 5.31E-05 | 3.00E-05 | CEBPB/PCNA/UCP2/IL6 | 4 |
| BP | GO:0033002 | muscle cell proliferation | 7/41 | 248/18723 | 1.01E-06 | 5.31E-05 | 3.00E-05 | JUN/CDKN1A/IGFBP3/ELN/CDK1/PTGS2/IL6 | 7 |
| BP | GO:1990845 | adaptive thermogenesis | 6/41 | 157/18723 | 1.11E-06 | 5.72E-05 | 3.24E-05 | DDIT3/VEGFA/CEBPB/ARNTL/UCP2/UCP3 | 6 |
| BP | GO:0010038 | response to metal ion | 8/41 | 373/18723 | 1.24E-06 | 6.20E-05 | 3.51E-05 | JUN/FOS/PCNA/CDK1/CEBPA/C1QA/PTGS2/JUND | 8 |
| BP | GO:0036294 | cellular response to decreased oxygen levels | 6/41 | 161/18723 | 1.29E-06 | 6.31E-05 | 3.57E-05 | VEGFA/CCNA2/PCK1/EGR1/MYC/PTGS2 | 6 |
| BP | GO:0030099 | myeloid cell differentiation | 8/41 | 381/18723 | 1.45E-06 | 6.92E-05 | 3.91E-05 | JUN/VEGFA/HMGB2/CEBPB/FOS/CDKN2B/MYC/CEBPA | 8 |
| BP | GO:1901216 | positive regulation of neuron death | 5/41 | 97/18723 | 2.17E-06 | 0.00010129 | 5.73E-05 | DDIT3/JUN/FOS/EGR1/C1QA | 5 |
| BP | GO:0071453 | cellular response to oxygen levels | 6/41 | 177/18723 | 2.24E-06 | 0.000102 | 5.77E-05 | VEGFA/CCNA2/PCK1/EGR1/MYC/PTGS2 | 6 |
| BP | GO:0009266 | response to temperature stimulus | 6/41 | 178/18723 | 2.31E-06 | 0.00010304 | 5.83E-05 | CDKN1A/FOS/UCP2/PTGS2/UCP3/NGFR | 6 |
| BP | GO:0048660 | regulation of smooth muscle cell proliferation | 6/41 | 180/18723 | 2.47E-06 | 0.00010755 | 6.08E-05 | JUN/CDKN1A/IGFBP3/ELN/PTGS2/IL6 | 6 |
| BP | GO:0048659 | smooth muscle cell proliferation | 6/41 | 184/18723 | 2.81E-06 | 0.00011955 | 6.76E-05 | JUN/CDKN1A/IGFBP3/ELN/PTGS2/IL6 | 6 |
| BP | GO:0048146 | positive regulation of fibroblast proliferation | 4/41 | 48/18723 | 3.59E-06 | 0.00014985 | 8.47E-05 | CDKN1A/CCNA2/MYC/NGFR | 4 |
| BP | GO:0070741 | response to interleukin-6 | 4/41 | 49/18723 | 3.90E-06 | 0.00015958 | 9.02E-05 | PCK1/RELA/CEBPA/IL6 | 4 |
| BP | GO:0042594 | response to starvation | 6/41 | 197/18723 | 4.16E-06 | 0.00016668 | 9.42E-05 | DDIT3/JUN/CDKN1A/PCK1/UCP2/HSPA8 | 6 |
| BP | GO:0071347 | cellular response to interleukin-1 | 5/41 | 113/18723 | 4.62E-06 | 0.0001813 | 0.00010252 | CEBPB/PCK1/EGR1/RELA/IL6 | 5 |
| BP | GO:1901214 | regulation of neuron death | 7/41 | 319/18723 | 5.34E-06 | 0.00020552 | 0.00011621 | DDIT3/JUN/CEBPB/FOS/EGR1/NGF/C1QA | 7 |
| BP | GO:0009416 | response to light stimulus | 7/41 | 320/18723 | 5.45E-06 | 0.00020582 | 0.00011639 | CDKN1A/FOS/PCNA/RELA/MYC/PTGS2/JUND | 7 |
| BP | GO:0002573 | myeloid leukocyte differentiation | 6/41 | 208/18723 | 5.69E-06 | 0.00021093 | 0.00011927 | JUN/VEGFA/CEBPB/FOS/MYC/CEBPA | 6 |
| BP | GO:0001819 | positive regulation of cytokine production | 8/41 | 467/18723 | 6.54E-06 | 0.00023817 | 0.00013468 | DDIT3/HMGB2/CEBPB/EGR1/RELA/PTGS2/IL6/SERPINE1 | 8 |
| BP | GO:0031669 | cellular response to nutrient levels | 6/41 | 215/18723 | 6.87E-06 | 0.00024589 | 0.00013904 | JUN/CDKN1A/PCK1/UCP2/HSPA8/CDKN2B | 6 |
| BP | GO:0071241 | cellular response to inorganic substance | 6/41 | 226/18723 | 9.14E-06 | 0.00032128 | 0.00018167 | JUN/CCNA2/FOS/CEBPA/PTGS2/JUND | 6 |
| BP | GO:0046683 | response to organophosphorus | 5/41 | 131/18723 | 9.51E-06 | 0.00032838 | 0.00018569 | JUN/FOS/PCK1/RELA/PTGS2 | 5 |
| BP | GO:0045444 | fat cell differentiation | 6/41 | 229/18723 | 9.86E-06 | 0.0003294 | 0.00018626 | DDIT3/CEBPB/ARNTL/CEBPA/PTGS2/IL6 | 6 |
| BP | GO:0007569 | cell aging | 5/41 | 132/18723 | 9.87E-06 | 0.0003294 | 0.00018626 | CDKN1A/ARNTL/CDK1/CDKN2B/SERPINE1 | 5 |
| BP | GO:0032757 | positive regulation of interleukin-8 production | 4/41 | 62/18723 | 1.01E-05 | 0.00033064 | 0.00018697 | DDIT3/RELA/IL6/SERPINE1 | 4 |
| BP | GO:0001101 | response to acid chemical | 5/41 | 135/18723 | 1.10E-05 | 0.00035558 | 0.00020107 | VEGFA/CEBPB/PCK1/PCNA/RELA | 5 |
| BP | GO:1901653 | cellular response to peptide | 7/41 | 359/18723 | 1.15E-05 | 0.00036647 | 0.00020723 | IRS2/CCNA2/CACNA1A/PCK1/RELA/UCP2/NGFR | 7 |
| BP | GO:0050767 | regulation of neurogenesis | 7/41 | 364/18723 | 1.26E-05 | 0.00039457 | 0.00022312 | VEGFA/HMGB2/ARNTL/NGF/RELA/IL6/DLL3 | 7 |
| BP | GO:0050729 | positive regulation of inflammatory response | 5/41 | 142/18723 | 1.41E-05 | 0.00043069 | 0.00024354 | CEBPB/CEBPA/PTGS2/IL6/SERPINE1 | 5 |
| BP | GO:0070555 | response to interleukin-1 | 5/41 | 143/18723 | 1.46E-05 | 0.00043069 | 0.00024354 | CEBPB/PCK1/EGR1/RELA/IL6 | 5 |
| BP | GO:0046686 | response to cadmium ion | 4/41 | 68/18723 | 1.46E-05 | 0.00043069 | 0.00024354 | JUN/FOS/PCNA/CDK1 | 4 |
| BP | GO:0071216 | cellular response to biotic stimulus | 6/41 | 246/18723 | 1.48E-05 | 0.00043069 | 0.00024354 | DDIT3/HMGB2/CEBPB/RELA/IL6/SERPINE1 | 6 |
| BP | GO:0106106 | cold-induced thermogenesis | 5/41 | 144/18723 | 1.51E-05 | 0.00043069 | 0.00024354 | DDIT3/VEGFA/CEBPB/ARNTL/UCP2 | 5 |
| BP | GO:0120161 | regulation of cold-induced thermogenesis | 5/41 | 144/18723 | 1.51E-05 | 0.00043069 | 0.00024354 | DDIT3/VEGFA/CEBPB/ARNTL/UCP2 | 5 |
| BP | GO:0045669 | positive regulation of osteoblast differentiation | 4/41 | 69/18723 | 1.54E-05 | 0.00043539 | 0.0002462 | CEBPB/CEBPA/JUND/IL6 | 4 |
| BP | GO:0042542 | response to hydrogen peroxide | 5/41 | 146/18723 | 1.61E-05 | 0.00044762 | 0.00025311 | JUN/PCNA/CDK1/RELA/IL6 | 5 |
| BP | GO:0014074 | response to purine-containing compound | 5/41 | 148/18723 | 1.72E-05 | 0.0004715 | 0.00026662 | JUN/FOS/PCK1/RELA/PTGS2 | 5 |
| BP | GO:0009411 | response to UV | 5/41 | 149/18723 | 1.78E-05 | 0.00048051 | 0.00027171 | CDKN1A/PCNA/RELA/MYC/PTGS2 | 5 |
| BP | GO:0036003 | positive regulation of transcription from RNA polymerase II promoter in response to stress | 3/41 | 24/18723 | 1.91E-05 | 0.00051026 | 0.00028854 | DDIT3/VEGFA/CEBPB | 3 |
| BP | GO:0042593 | glucose homeostasis | 6/41 | 258/18723 | 1.94E-05 | 0.00051085 | 0.00028887 | IRS2/PCK1/UCP2/CEBPA/IL6/NGFR | 6 |
| BP | GO:0033500 | carbohydrate homeostasis | 6/41 | 259/18723 | 1.98E-05 | 0.00051534 | 0.00029141 | IRS2/PCK1/UCP2/CEBPA/IL6/NGFR | 6 |
| BP | GO:0032868 | response to insulin | 6/41 | 264/18723 | 2.21E-05 | 0.00056163 | 0.00031759 | IRS2/PCK1/EGR1/RELA/UCP2/UCP3 | 6 |
| BP | GO:0009267 | cellular response to starvation | 5/41 | 156/18723 | 2.22E-05 | 0.00056163 | 0.00031759 | JUN/CDKN1A/PCK1/UCP2/HSPA8 | 5 |
| BP | GO:0014823 | response to activity | 4/41 | 76/18723 | 2.27E-05 | 0.00056714 | 0.0003207 | PCK1/CDK1/UCP3/IL6 | 4 |
| BP | GO:0048145 | regulation of fibroblast proliferation | 4/41 | 80/18723 | 2.77E-05 | 0.00068616 | 0.000388 | CDKN1A/CCNA2/MYC/NGFR | 4 |
| BP | GO:0048144 | fibroblast proliferation | 4/41 | 81/18723 | 2.91E-05 | 0.00071188 | 0.00040254 | CDKN1A/CCNA2/MYC/NGFR | 4 |
| BP | GO:0031349 | positive regulation of defense response | 6/41 | 278/18723 | 2.95E-05 | 0.00071194 | 0.00040258 | HMGB2/CEBPB/CEBPA/PTGS2/IL6/SERPINE1 | 6 |
| BP | GO:0000303 | response to superoxide | 3/41 | 28/18723 | 3.07E-05 | 0.00073294 | 0.00041445 | PRDX1/UCP2/UCP3 | 3 |
| BP | GO:0000305 | response to oxygen radical | 3/41 | 29/18723 | 3.42E-05 | 0.00080627 | 0.00045592 | PRDX1/UCP2/UCP3 | 3 |
| BP | GO:1903131 | mononuclear cell differentiation | 7/41 | 426/18723 | 3.46E-05 | 0.00080627 | 0.00045592 | JUN/VEGFA/CEBPB/PCK1/EGR1/MYC/IL6 | 7 |
| BP | GO:0032103 | positive regulation of response to external stimulus | 7/41 | 427/18723 | 3.51E-05 | 0.00080899 | 0.00045746 | VEGFA/HMGB2/CEBPB/CEBPA/PTGS2/IL6/SERPINE1 | 7 |
| BP | GO:0070498 | interleukin-1-mediated signaling pathway | 3/41 | 30/18723 | 3.80E-05 | 0.00086442 | 0.0004888 | EGR1/RELA/IL6 | 3 |
| BP | GO:0050673 | epithelial cell proliferation | 7/41 | 437/18723 | 4.07E-05 | 0.00091616 | 0.00051806 | VEGFA/HMGB2/CEBPB/CDKN2B/MYC/IL6/NGFR | 7 |
| BP | GO:0034644 | cellular response to UV | 4/41 | 90/18723 | 4.41E-05 | 0.00097697 | 0.00055244 | CDKN1A/PCNA/MYC/PTGS2 | 4 |
| BP | GO:0051960 | regulation of nervous system development | 7/41 | 443/18723 | 4.44E-05 | 0.00097697 | 0.00055244 | VEGFA/HMGB2/ARNTL/NGF/RELA/IL6/DLL3 | 7 |
| BP | GO:0002065 | columnar/cuboidal epithelial cell differentiation | 4/41 | 91/18723 | 4.61E-05 | 0.00099623 | 0.00056333 | CDKN1A/CEBPB/ARNTL/SERPINE1 | 4 |
| BP | GO:0045736 | negative regulation of cyclin-dependent protein serine/threonine kinase activity | 3/41 | 32/18723 | 4.63E-05 | 0.00099623 | 0.00056333 | CDKN1A/CDKN2B/CEBPA | 3 |
| BP | GO:0051591 | response to cAMP | 4/41 | 93/18723 | 5.02E-05 | 0.00106935 | 0.00060468 | JUN/FOS/PCK1/RELA | 4 |
| BP | GO:1904030 | negative regulation of cyclin-dependent protein kinase activity | 3/41 | 33/18723 | 5.08E-05 | 0.00107115 | 0.0006057 | CDKN1A/CDKN2B/CEBPA | 3 |
| BP | GO:0071478 | cellular response to radiation | 5/41 | 186/18723 | 5.14E-05 | 0.00107258 | 0.00060651 | CDKN1A/EGR1/PCNA/MYC/PTGS2 | 5 |
| BP | GO:0000079 | regulation of cyclin-dependent protein serine/threonine kinase activity | 4/41 | 94/18723 | 5.23E-05 | 0.00108062 | 0.00061106 | CDKN1A/CCNA2/CDKN2B/CEBPA | 4 |
| BP | GO:0018105 | peptidyl-serine phosphorylation | 6/41 | 315/18723 | 5.92E-05 | 0.00121009 | 0.00068427 | VEGFA/PCK1/CDK1/NGF/PTGS2/IL6 | 6 |
| BP | GO:0035633 | maintenance of blood-brain barrier | 3/41 | 35/18723 | 6.08E-05 | 0.00122161 | 0.00069078 | VEGFA/PTGS2/IL6 | 3 |
| BP | GO:0070301 | cellular response to hydrogen peroxide | 4/41 | 98/18723 | 6.16E-05 | 0.00122161 | 0.00069078 | PCNA/CDK1/RELA/IL6 | 4 |
| BP | GO:1904029 | regulation of cyclin-dependent protein kinase activity | 4/41 | 98/18723 | 6.16E-05 | 0.00122161 | 0.00069078 | CDKN1A/CCNA2/CDKN2B/CEBPA | 4 |
| BP | GO:1901654 | response to ketone | 5/41 | 194/18723 | 6.28E-05 | 0.00123297 | 0.00069721 | CDKN1A/FOS/PCK1/PCNA/RELA | 5 |
| BP | GO:0030224 | monocyte differentiation | 3/41 | 36/18723 | 6.62E-05 | 0.001287 | 0.00072776 | JUN/VEGFA/MYC | 3 |
| BP | GO:0071248 | cellular response to metal ion | 5/41 | 197/18723 | 6.75E-05 | 0.00130047 | 0.00073537 | JUN/FOS/CEBPA/PTGS2/JUND | 5 |
| BP | GO:0032677 | regulation of interleukin-8 production | 4/41 | 102/18723 | 7.20E-05 | 0.00137368 | 0.00077677 | DDIT3/RELA/IL6/SERPINE1 | 4 |
| BP | GO:0032637 | interleukin-8 production | 4/41 | 103/18723 | 7.48E-05 | 0.00141347 | 0.00079927 | DDIT3/RELA/IL6/SERPINE1 | 4 |
| BP | GO:0071214 | cellular response to abiotic stimulus | 6/41 | 331/18723 | 7.79E-05 | 0.00144394 | 0.0008165 | CDKN1A/PCK1/EGR1/PCNA/MYC/PTGS2 | 6 |
| BP | GO:0104004 | cellular response to environmental stimulus | 6/41 | 331/18723 | 7.79E-05 | 0.00144394 | 0.0008165 | CDKN1A/PCK1/EGR1/PCNA/MYC/PTGS2 | 6 |
| BP | GO:0042116 | macrophage activation | 4/41 | 106/18723 | 8.36E-05 | 0.00152317 | 0.00086131 | CEBPA/C1QA/JUND/IL6 | 4 |
| BP | GO:2000278 | regulation of DNA biosynthetic process | 4/41 | 106/18723 | 8.36E-05 | 0.00152317 | 0.00086131 | CDKN1A/PCNA/TFDP1/MYC | 4 |
| BP | GO:0006275 | regulation of DNA replication | 4/41 | 107/18723 | 8.68E-05 | 0.00153504 | 0.00086802 | JUN/CCNA2/PCNA/CDK1 | 4 |
| BP | GO:0042303 | molting cycle | 4/41 | 107/18723 | 8.68E-05 | 0.00153504 | 0.00086802 | ARNTL/RELA/PTGS2/NGFR | 4 |
| BP | GO:0042633 | hair cycle | 4/41 | 107/18723 | 8.68E-05 | 0.00153504 | 0.00086802 | ARNTL/RELA/PTGS2/NGFR | 4 |
| BP | GO:0018209 | peptidyl-serine modification | 6/41 | 338/18723 | 8.74E-05 | 0.00153504 | 0.00086802 | VEGFA/PCK1/CDK1/NGF/PTGS2/IL6 | 6 |
| BP | GO:0071222 | cellular response to lipopolysaccharide | 5/41 | 209/18723 | 8.93E-05 | 0.00155006 | 0.00087651 | HMGB2/CEBPB/RELA/IL6/SERPINE1 | 5 |
| BP | GO:0033138 | positive regulation of peptidyl-serine phosphorylation | 4/41 | 108/18723 | 9.00E-05 | 0.00155006 | 0.00087651 | VEGFA/NGF/PTGS2/IL6 | 4 |
| BP | GO:0045740 | positive regulation of DNA replication | 3/41 | 40/18723 | 9.10E-05 | 0.00155006 | 0.00087651 | JUN/PCNA/CDK1 | 3 |
| BP | GO:0045637 | regulation of myeloid cell differentiation | 5/41 | 210/18723 | 9.13E-05 | 0.00155006 | 0.00087651 | JUN/HMGB2/CEBPB/FOS/MYC | 5 |
| BP | GO:0009612 | response to mechanical stimulus | 5/41 | 216/18723 | 0.00010426 | 0.00175494 | 0.00099236 | JUN/FOS/RELA/PTGS2/JUND | 5 |
| BP | GO:0009746 | response to hexose | 5/41 | 219/18723 | 0.00011124 | 0.00185682 | 0.00104997 | IRS2/PCK1/EGR1/UCP2/PTGS2 | 5 |
| BP | GO:0071219 | cellular response to molecule of bacterial origin | 5/41 | 221/18723 | 0.00011609 | 0.00190662 | 0.00107813 | HMGB2/CEBPB/RELA/IL6/SERPINE1 | 5 |
| BP | GO:2001233 | regulation of apoptotic signaling pathway | 6/41 | 356/18723 | 0.00011613 | 0.00190662 | 0.00107813 | DDIT3/HMGB2/RELA/PTGS2/NGFR/SERPINE1 | 6 |
| BP | GO:0043200 | response to amino acid | 4/41 | 116/18723 | 0.00011868 | 0.00193266 | 0.00109285 | CEBPB/PCK1/PCNA/RELA | 4 |
| BP | GO:0071900 | regulation of protein serine/threonine kinase activity | 6/41 | 359/18723 | 0.00012158 | 0.00196393 | 0.00111054 | CDKN1A/VEGFA/CCNA2/CDKN2B/CEBPA/UCHL1 | 6 |
| BP | GO:0034284 | response to monosaccharide | 5/41 | 225/18723 | 0.00012628 | 0.00201559 | 0.00113975 | IRS2/PCK1/EGR1/UCP2/PTGS2 | 5 |
| BP | GO:0051101 | regulation of DNA binding | 4/41 | 118/18723 | 0.00012679 | 0.00201559 | 0.00113975 | DDIT3/JUN/HMGB2/NGF | 4 |
| BP | GO:0071354 | cellular response to interleukin-6 | 3/41 | 45/18723 | 0.00012974 | 0.00204623 | 0.00115708 | RELA/CEBPA/IL6 | 3 |
| BP | GO:0002761 | regulation of myeloid leukocyte differentiation | 4/41 | 120/18723 | 0.0001353 | 0.00209678 | 0.00118566 | JUN/CEBPB/FOS/MYC | 4 |
| BP | GO:0071901 | negative regulation of protein serine/threonine kinase activity | 4/41 | 120/18723 | 0.0001353 | 0.00209678 | 0.00118566 | CDKN1A/CDKN2B/CEBPA/UCHL1 | 4 |
| BP | GO:1903706 | regulation of hemopoiesis | 6/41 | 367/18723 | 0.00013712 | 0.00209678 | 0.00118566 | JUN/HMGB2/CEBPB/FOS/PCK1/MYC | 6 |
| BP | GO:0071695 | anatomical structure maturation | 5/41 | 229/18723 | 0.00013713 | 0.00209678 | 0.00118566 | DDIT3/CDKN1A/VEGFA/CEBPA/C1QA | 5 |
| BP | GO:0071482 | cellular response to light stimulus | 4/41 | 123/18723 | 0.00014882 | 0.00225829 | 0.00127699 | CDKN1A/PCNA/MYC/PTGS2 | 4 |
| BP | GO:0033673 | negative regulation of kinase activity | 5/41 | 237/18723 | 0.00016099 | 0.00242446 | 0.00137095 | IRS2/CDKN1A/CDKN2B/CEBPA/UCHL1 | 5 |
| BP | GO:0006953 | acute-phase response | 3/41 | 49/18723 | 0.00016743 | 0.0024842 | 0.00140473 | CEBPB/PTGS2/IL6 | 3 |
| BP | GO:0009409 | response to cold | 3/41 | 49/18723 | 0.00016743 | 0.0024842 | 0.00140473 | FOS/UCP2/UCP3 | 3 |
| BP | GO:0014706 | striated muscle tissue development | 6/41 | 384/18723 | 0.00017539 | 0.00256321 | 0.00144941 | VEGFA/ARNTL/FOS/ELN/EGR1/CDK1 | 6 |
| BP | GO:0050873 | brown fat cell differentiation | 3/41 | 50/18723 | 0.00017785 | 0.00256321 | 0.00144941 | CEBPB/CEBPA/PTGS2 | 3 |
| BP | GO:0042326 | negative regulation of phosphorylation | 6/41 | 385/18723 | 0.00017788 | 0.00256321 | 0.00144941 | IRS2/CDKN1A/IGFBP3/CDKN2B/CEBPA/UCHL1 | 6 |
| BP | GO:0045786 | negative regulation of cell cycle | 6/41 | 385/18723 | 0.00017788 | 0.00256321 | 0.00144941 | CDKN1A/BUB1B/CDK1/CDKN2B/CEBPA/PTGS2 | 6 |
| BP | GO:0050727 | regulation of inflammatory response | 6/41 | 386/18723 | 0.00018039 | 0.00258092 | 0.00145943 | CEBPB/RELA/CEBPA/PTGS2/IL6/SERPINE1 | 6 |
| BP | GO:0051402 | neuron apoptotic process | 5/41 | 246/18723 | 0.00019148 | 0.00272016 | 0.00153816 | DDIT3/JUN/CEBPB/NGF/NGFR | 5 |
| BP | GO:0045667 | regulation of osteoblast differentiation | 4/41 | 132/18723 | 0.00019528 | 0.00275451 | 0.00155759 | CEBPB/CEBPA/JUND/IL6 | 4 |
| BP | GO:0009750 | response to fructose | 2/41 | 10/18723 | 0.00020821 | 0.00289615 | 0.00163768 | PCK1/PTGS2 | 2 |
| BP | GO:0072203 | cell proliferation involved in metanephros development | 2/41 | 10/18723 | 0.00020821 | 0.00289615 | 0.00163768 | EGR1/MYC | 2 |
| BP | GO:0009743 | response to carbohydrate | 5/41 | 253/18723 | 0.00021811 | 0.00301297 | 0.00170373 | IRS2/PCK1/EGR1/UCP2/PTGS2 | 5 |
| BP | GO:0000086 | G2/M transition of mitotic cell cycle | 4/41 | 137/18723 | 0.00022519 | 0.0030816 | 0.00174254 | CDKN1A/CCNA2/CDK1/CDKN2B | 4 |
| BP | GO:0007611 | learning or memory | 5/41 | 255/18723 | 0.00022622 | 0.0030816 | 0.00174254 | JUN/CEBPB/FOS/NGF/PTGS2 | 5 |
| BP | GO:0060537 | muscle tissue development | 6/41 | 403/18723 | 0.0002277 | 0.0030816 | 0.00174254 | VEGFA/ARNTL/FOS/ELN/EGR1/CDK1 | 6 |
| BP | GO:0002696 | positive regulation of leukocyte activation | 6/41 | 409/18723 | 0.00024655 | 0.00328449 | 0.00185727 | IRS2/CDKN1A/PCK1/CEBPA/JUND/IL6 | 6 |
| BP | GO:0051091 | positive regulation of DNA-binding transcription factor activity | 5/41 | 260/18723 | 0.00024748 | 0.00328449 | 0.00185727 | DDIT3/VEGFA/TFDP1/RELA/IL6 | 5 |
| BP | GO:0010332 | response to gamma radiation | 3/41 | 56/18723 | 0.00024925 | 0.00328449 | 0.00185727 | CDKN1A/EGR1/MYC | 3 |
| BP | GO:0045599 | negative regulation of fat cell differentiation | 3/41 | 56/18723 | 0.00024925 | 0.00328449 | 0.00185727 | DDIT3/ARNTL/IL6 | 3 |
| BP | GO:1990440 | positive regulation of transcription from RNA polymerase II promoter in response to endoplasmic reticulum stress | 2/41 | 11/18723 | 0.00025413 | 0.0033269 | 0.00188125 | DDIT3/CEBPB | 2 |
| BP | GO:0009636 | response to toxic substance | 5/41 | 262/18723 | 0.0002564 | 0.00333485 | 0.00188575 | CDKN1A/PRDX1/FOS/CDK1/PTGS2 | 5 |
| BP | GO:0033135 | regulation of peptidyl-serine phosphorylation | 4/41 | 144/18723 | 0.00027243 | 0.00352053 | 0.00199075 | VEGFA/NGF/PTGS2/IL6 | 4 |
| BP | GO:0050867 | positive regulation of cell activation | 6/41 | 420/18723 | 0.00028431 | 0.00363132 | 0.00205339 | IRS2/CDKN1A/PCK1/CEBPA/JUND/IL6 | 6 |
| BP | GO:0051348 | negative regulation of transferase activity | 5/41 | 268/18723 | 0.00028463 | 0.00363132 | 0.00205339 | IRS2/CDKN1A/CDKN2B/CEBPA/UCHL1 | 5 |
| BP | GO:0044772 | mitotic cell cycle phase transition | 6/41 | 424/18723 | 0.00029912 | 0.00379195 | 0.00214422 | CDKN1A/CCNA2/BUB1B/CDK1/TFDP1/CDKN2B | 6 |
| BP | GO:0044839 | cell cycle G2/M phase transition | 4/41 | 148/18723 | 0.00030242 | 0.00380975 | 0.00215429 | CDKN1A/CCNA2/CDK1/CDKN2B | 4 |
| BP | GO:0043030 | regulation of macrophage activation | 3/41 | 61/18723 | 0.00032116 | 0.00402052 | 0.00227347 | CEBPA/JUND/IL6 | 3 |
| BP | GO:0052548 | regulation of endopeptidase activity | 6/41 | 432/18723 | 0.00033055 | 0.00411236 | 0.00232541 | VEGFA/NGF/MYC/PTGS2/NGFR/SERPINE1 | 6 |
| BP | GO:1902105 | regulation of leukocyte differentiation | 5/41 | 279/18723 | 0.00034247 | 0.00423434 | 0.00239438 | JUN/CEBPB/FOS/PCK1/MYC | 5 |
| BP | GO:0021700 | developmental maturation | 5/41 | 280/18723 | 0.00034814 | 0.00427802 | 0.00241908 | DDIT3/CDKN1A/VEGFA/CEBPA/C1QA | 5 |
| BP | GO:1904589 | regulation of protein import | 3/41 | 63/18723 | 0.00035329 | 0.00431488 | 0.00243992 | CDK1/HSPA8/PTGS2 | 3 |
| BP | GO:0032494 | response to peptidoglycan | 2/41 | 13/18723 | 0.0003594 | 0.00432385 | 0.002445 | RELA/IL6 | 2 |
| BP | GO:0061418 | regulation of transcription from RNA polymerase II promoter in response to hypoxia | 2/41 | 13/18723 | 0.0003594 | 0.00432385 | 0.002445 | VEGFA/EGR1 | 2 |
| BP | GO:0007519 | skeletal muscle tissue development | 4/41 | 155/18723 | 0.0003605 | 0.00432385 | 0.002445 | ARNTL/FOS/ELN/EGR1 | 4 |
| BP | GO:0045936 | negative regulation of phosphate metabolic process | 6/41 | 441/18723 | 0.00036896 | 0.00439896 | 0.00248747 | IRS2/CDKN1A/IGFBP3/CDKN2B/CEBPA/UCHL1 | 6 |
| BP | GO:0010563 | negative regulation of phosphorus metabolic process | 6/41 | 442/18723 | 0.00037343 | 0.00442597 | 0.00250274 | IRS2/CDKN1A/IGFBP3/CDKN2B/CEBPA/UCHL1 | 6 |
| BP | GO:0022411 | cellular component disassembly | 6/41 | 443/18723 | 0.00037795 | 0.00443313 | 0.00250679 | TOP2A/CDK1/HSPA8/MYC/C1QA/IL6 | 6 |
| BP | GO:0002262 | myeloid cell homeostasis | 4/41 | 157/18723 | 0.00037847 | 0.00443313 | 0.00250679 | VEGFA/HMGB2/PRDX1/IL6 | 4 |
| BP | GO:0045600 | positive regulation of fat cell differentiation | 3/41 | 66/18723 | 0.00040526 | 0.00471934 | 0.00266864 | CEBPB/CEBPA/PTGS2 | 3 |
| BP | GO:0071375 | cellular response to peptide hormone stimulus | 5/41 | 290/18723 | 0.00040882 | 0.0047333 | 0.00267653 | IRS2/CCNA2/PCK1/RELA/UCP2 | 5 |
| BP | GO:1901722 | regulation of cell proliferation involved in kidney development | 2/41 | 14/18723 | 0.00041872 | 0.00482005 | 0.00272558 | EGR1/MYC | 2 |
| BP | GO:0035914 | skeletal muscle cell differentiation | 3/41 | 67/18723 | 0.00042361 | 0.0048485 | 0.00274167 | ARNTL/FOS/EGR1 | 3 |
| BP | GO:0032922 | circadian regulation of gene expression | 3/41 | 68/18723 | 0.00044249 | 0.00503581 | 0.00284759 | ARNTL/EGR1/NGFR | 3 |
| BP | GO:0050890 | cognition | 5/41 | 296/18723 | 0.00044888 | 0.00507969 | 0.0028724 | JUN/CEBPB/FOS/NGF/PTGS2 | 5 |
| BP | GO:0050796 | regulation of insulin secretion | 4/41 | 165/18723 | 0.00045678 | 0.00514 | 0.0029065 | IRS2/ARNTL/UCP2/IL6 | 4 |
| BP | GO:0052547 | regulation of peptidase activity | 6/41 | 461/18723 | 0.00046688 | 0.00520019 | 0.00294054 | VEGFA/NGF/MYC/PTGS2/NGFR/SERPINE1 | 6 |
| BP | GO:0060538 | skeletal muscle organ development | 4/41 | 166/18723 | 0.00046732 | 0.00520019 | 0.00294054 | ARNTL/FOS/ELN/EGR1 | 4 |
| BP | GO:1901990 | regulation of mitotic cell cycle phase transition | 5/41 | 299/18723 | 0.00046999 | 0.00520104 | 0.00294102 | CDKN1A/BUB1B/CDK1/TFDP1/CDKN2B | 5 |
| BP | GO:0048569 | post-embryonic animal organ development | 2/41 | 15/18723 | 0.00048246 | 0.00530976 | 0.0030025 | VEGFA/EFEMP1 | 2 |
| BP | GO:0048469 | cell maturation | 4/41 | 171/18723 | 0.00052264 | 0.00572052 | 0.00323477 | CDKN1A/VEGFA/CEBPA/C1QA | 4 |
| BP | GO:0001678 | cellular glucose homeostasis | 4/41 | 172/18723 | 0.00053425 | 0.00581572 | 0.0032886 | IRS2/PCK1/UCP2/NGFR | 4 |
| BP | GO:0044849 | estrous cycle | 2/41 | 16/18723 | 0.00055062 | 0.00596162 | 0.0033711 | EGR1/PCNA | 2 |
| BP | GO:0007584 | response to nutrient | 4/41 | 174/18723 | 0.000558 | 0.00600895 | 0.00339787 | RELA/CDKN2B/PTGS2/UCP3 | 4 |
| BP | GO:0045685 | regulation of glial cell differentiation | 3/41 | 76/18723 | 0.00061321 | 0.00656822 | 0.00371412 | CDK1/RELA/IL6 | 3 |
| BP | GO:1901991 | negative regulation of mitotic cell cycle phase transition | 4/41 | 179/18723 | 0.00062065 | 0.00656958 | 0.00371488 | CDKN1A/BUB1B/CDK1/CDKN2B | 4 |
| BP | GO:0010224 | response to UV-B | 2/41 | 17/18723 | 0.00062318 | 0.00656958 | 0.00371488 | CDKN1A/RELA | 2 |
| BP | GO:0090399 | replicative senescence | 2/41 | 17/18723 | 0.00062318 | 0.00656958 | 0.00371488 | CDKN1A/SERPINE1 | 2 |
| BP | GO:0071897 | DNA biosynthetic process | 4/41 | 180/18723 | 0.00063376 | 0.00664616 | 0.00375819 | CDKN1A/PCNA/TFDP1/MYC | 4 |
| BP | GO:0007422 | peripheral nervous system development | 3/41 | 80/18723 | 0.00071233 | 0.00743119 | 0.0042021 | CDK1/NGF/RELA | 3 |
| BP | GO:0008625 | extrinsic apoptotic signaling pathway via death domain receptors | 3/41 | 82/18723 | 0.00076549 | 0.00794443 | 0.00449232 | HMGB2/NGF/SERPINE1 | 3 |
| BP | GO:0007265 | Ras protein signal transduction | 5/41 | 337/18723 | 0.00080741 | 0.00833628 | 0.0047139 | JUN/CDKN1A/CCNA2/NGF/NGFR | 5 |
| BP | GO:0007565 | female pregnancy | 4/41 | 193/18723 | 0.00082254 | 0.00844893 | 0.0047776 | VEGFA/FOS/UCP2/PTGS2 | 4 |
| BP | GO:0030073 | insulin secretion | 4/41 | 195/18723 | 0.00085475 | 0.00873497 | 0.00493935 | IRS2/ARNTL/UCP2/IL6 | 4 |
| BP | GO:0001933 | negative regulation of protein phosphorylation | 5/41 | 342/18723 | 0.00086255 | 0.00877002 | 0.00495916 | CDKN1A/IGFBP3/CDKN2B/CEBPA/UCHL1 | 5 |
| BP | GO:0090276 | regulation of peptide hormone secretion | 4/41 | 196/18723 | 0.00087118 | 0.00881299 | 0.00498346 | IRS2/ARNTL/UCP2/IL6 | 4 |
| BP | GO:1900182 | positive regulation of protein localization to nucleus | 3/41 | 87/18723 | 0.00090927 | 0.00915214 | 0.00517524 | CDK1/PTGS2/NGFR | 3 |
| BP | GO:0002791 | regulation of peptide secretion | 4/41 | 200/18723 | 0.00093915 | 0.00936666 | 0.00529655 | IRS2/ARNTL/UCP2/IL6 | 4 |
| BP | GO:0050829 | defense response to Gram-negative bacterium | 3/41 | 88/18723 | 0.00093994 | 0.00936666 | 0.00529655 | HMGB2/IL6/SERPINE1 | 3 |
| BP | GO:0033762 | response to glucagon | 2/41 | 21/18723 | 0.00095693 | 0.00939577 | 0.005313 | CCNA2/PCK1 | 2 |
| BP | GO:0045655 | regulation of monocyte differentiation | 2/41 | 21/18723 | 0.00095693 | 0.00939577 | 0.005313 | JUN/MYC | 2 |
| BP | GO:0072111 | cell proliferation involved in kidney development | 2/41 | 21/18723 | 0.00095693 | 0.00939577 | 0.005313 | EGR1/MYC | 2 |
| BP | GO:0014910 | regulation of smooth muscle cell migration | 3/41 | 89/18723 | 0.00097126 | 0.00947542 | 0.00535804 | IGFBP3/PLAU/SERPINE1 | 3 |
| BP | GO:0090087 | regulation of peptide transport | 4/41 | 202/18723 | 0.00097451 | 0.00947542 | 0.00535804 | IRS2/ARNTL/UCP2/IL6 | 4 |
| BP | GO:0032869 | cellular response to insulin stimulus | 4/41 | 203/18723 | 0.00099253 | 0.00960405 | 0.00543078 | IRS2/PCK1/RELA/UCP2 | 4 |
| BP | GO:0017038 | protein import | 4/41 | 206/18723 | 0.00104801 | 0.00974764 | 0.00551197 | CDKN1A/CDK1/HSPA8/PTGS2 | 4 |
| BP | GO:0002053 | positive regulation of mesenchymal cell proliferation | 2/41 | 22/18723 | 0.00105117 | 0.00974764 | 0.00551197 | IRS2/MYC | 2 |
| BP | GO:0032069 | regulation of nuclease activity | 2/41 | 22/18723 | 0.00105117 | 0.00974764 | 0.00551197 | HMGB2/PCNA | 2 |
| BP | GO:0051412 | response to corticosterone | 2/41 | 22/18723 | 0.00105117 | 0.00974764 | 0.00551197 | CDKN1A/FOS | 2 |
| BP | GO:0060563 | neuroepithelial cell differentiation | 2/41 | 22/18723 | 0.00105117 | 0.00974764 | 0.00551197 | CEBPB/SERPINE1 | 2 |
| BP | GO:0061042 | vascular wound healing | 2/41 | 22/18723 | 0.00105117 | 0.00974764 | 0.00551197 | VEGFA/SERPINE1 | 2 |
| BP | GO:0072574 | hepatocyte proliferation | 2/41 | 22/18723 | 0.00105117 | 0.00974764 | 0.00551197 | CEBPB/IL6 | 2 |
| BP | GO:0072575 | epithelial cell proliferation involved in liver morphogenesis | 2/41 | 22/18723 | 0.00105117 | 0.00974764 | 0.00551197 | CEBPB/IL6 | 2 |
| BP | GO:1901522 | positive regulation of transcription from RNA polymerase II promoter involved in cellular response to chemical stimulus | 2/41 | 22/18723 | 0.00105117 | 0.00974764 | 0.00551197 | VEGFA/RELA | 2 |
| BP | GO:0051052 | regulation of DNA metabolic process | 5/41 | 359/18723 | 0.00107129 | 0.00988847 | 0.00559161 | CDKN1A/PCNA/TFDP1/MYC/IL6 | 5 |
| BP | GO:0090398 | cellular senescence | 3/41 | 93/18723 | 0.00110312 | 0.01011233 | 0.00571819 | CDKN1A/ARNTL/CDKN2B | 3 |
| BP | GO:0043281 | regulation of cysteine-type endopeptidase activity involved in apoptotic process | 4/41 | 209/18723 | 0.00110564 | 0.01011233 | 0.00571819 | VEGFA/MYC/PTGS2/NGFR | 4 |
| BP | GO:0051098 | regulation of binding | 5/41 | 363/18723 | 0.00112544 | 0.01024658 | 0.00579411 | DDIT3/JUN/CDKN1A/HMGB2/NGF | 5 |
| BP | GO:0002690 | positive regulation of leukocyte chemotaxis | 3/41 | 94/18723 | 0.00113776 | 0.01031189 | 0.00583104 | VEGFA/IL6/SERPINE1 | 3 |
| BP | GO:0035994 | response to muscle stretch | 2/41 | 23/18723 | 0.00114969 | 0.01032654 | 0.00583932 | FOS/RELA | 2 |
| BP | GO:0072576 | liver morphogenesis | 2/41 | 23/18723 | 0.00114969 | 0.01032654 | 0.00583932 | CEBPB/IL6 | 2 |
| BP | GO:0006469 | negative regulation of protein kinase activity | 4/41 | 212/18723 | 0.00116547 | 0.01032934 | 0.0058409 | CDKN1A/CDKN2B/CEBPA/UCHL1 | 4 |
| BP | GO:0009749 | response to glucose | 4/41 | 212/18723 | 0.00116547 | 0.01032934 | 0.0058409 | IRS2/PCK1/EGR1/UCP2 | 4 |
| BP | GO:0043523 | regulation of neuron apoptotic process | 4/41 | 212/18723 | 0.00116547 | 0.01032934 | 0.0058409 | DDIT3/JUN/CEBPB/NGF | 4 |
| BP | GO:0014909 | smooth muscle cell migration | 3/41 | 97/18723 | 0.00124579 | 0.01086011 | 0.00614104 | IGFBP3/PLAU/SERPINE1 | 3 |
| BP | GO:0120162 | positive regulation of cold-induced thermogenesis | 3/41 | 97/18723 | 0.00124579 | 0.01086011 | 0.00614104 | VEGFA/CEBPB/UCP2 | 3 |
| BP | GO:2001237 | negative regulation of extrinsic apoptotic signaling pathway | 3/41 | 97/18723 | 0.00124579 | 0.01086011 | 0.00614104 | HMGB2/RELA/SERPINE1 | 3 |
| BP | GO:0010226 | response to lithium ion | 2/41 | 24/18723 | 0.00125246 | 0.01086011 | 0.00614104 | CEBPA/PTGS2 | 2 |
| BP | GO:0031639 | plasminogen activation | 2/41 | 24/18723 | 0.00125246 | 0.01086011 | 0.00614104 | PLAU/SERPINE1 | 2 |
| BP | GO:0097191 | extrinsic apoptotic signaling pathway | 4/41 | 219/18723 | 0.00131385 | 0.01134325 | 0.00641424 | HMGB2/NGF/RELA/SERPINE1 | 4 |
| BP | GO:0044706 | multi-multicellular organism process | 4/41 | 220/18723 | 0.00133607 | 0.01144142 | 0.00646975 | VEGFA/FOS/UCP2/PTGS2 | 4 |
| BP | GO:0033233 | regulation of protein sumoylation | 2/41 | 25/18723 | 0.00135949 | 0.01144142 | 0.00646975 | EGR1/RELA | 2 |
| BP | GO:0042730 | fibrinolysis | 2/41 | 25/18723 | 0.00135949 | 0.01144142 | 0.00646975 | PLAU/SERPINE1 | 2 |
| BP | GO:0048143 | astrocyte activation | 2/41 | 25/18723 | 0.00135949 | 0.01144142 | 0.00646975 | C1QA/IL6 | 2 |
| BP | GO:0071459 | protein localization to chromosome, centromeric region | 2/41 | 25/18723 | 0.00135949 | 0.01144142 | 0.00646975 | BUB1B/CDK1 | 2 |
| BP | GO:1904996 | positive regulation of leukocyte adhesion to vascular endothelial cell | 2/41 | 25/18723 | 0.00135949 | 0.01144142 | 0.00646975 | RELA/IL6 | 2 |
| BP | GO:0050678 | regulation of epithelial cell proliferation | 5/41 | 381/18723 | 0.00139464 | 0.01168816 | 0.00660928 | VEGFA/HMGB2/CDKN2B/MYC/NGFR | 5 |
| BP | GO:0002274 | myeloid leukocyte activation | 4/41 | 223/18723 | 0.00140433 | 0.0117203 | 0.00662745 | CEBPA/C1QA/JUND/IL6 | 4 |
| BP | GO:2001234 | negative regulation of apoptotic signaling pathway | 4/41 | 224/18723 | 0.00142762 | 0.0118652 | 0.00670938 | HMGB2/RELA/PTGS2/SERPINE1 | 4 |
| BP | GO:0010001 | glial cell differentiation | 4/41 | 225/18723 | 0.00145117 | 0.01191267 | 0.00673623 | CDK1/RELA/C1QA/IL6 | 4 |
| BP | GO:0050670 | regulation of lymphocyte proliferation | 4/41 | 225/18723 | 0.00145117 | 0.01191267 | 0.00673623 | IRS2/CDKN1A/CEBPB/IL6 | 4 |
| BP | GO:0050769 | positive regulation of neurogenesis | 4/41 | 225/18723 | 0.00145117 | 0.01191267 | 0.00673623 | VEGFA/NGF/RELA/IL6 | 4 |
| BP | GO:0042634 | regulation of hair cycle | 2/41 | 26/18723 | 0.00147074 | 0.01197518 | 0.00677158 | ARNTL/NGFR | 2 |
| BP | GO:0070102 | interleukin-6-mediated signaling pathway | 2/41 | 26/18723 | 0.00147074 | 0.01197518 | 0.00677158 | CEBPA/IL6 | 2 |
| BP | GO:0045639 | positive regulation of myeloid cell differentiation | 3/41 | 103/18723 | 0.00148078 | 0.01200807 | 0.00679017 | JUN/HMGB2/FOS | 3 |
| BP | GO:0032944 | regulation of mononuclear cell proliferation | 4/41 | 227/18723 | 0.00149909 | 0.01210756 | 0.00684643 | IRS2/CDKN1A/CEBPB/IL6 | 4 |
| BP | GO:0048661 | positive regulation of smooth muscle cell proliferation | 3/41 | 104/18723 | 0.00152245 | 0.01224687 | 0.00692521 | JUN/PTGS2/IL6 | 3 |
| BP | GO:1901987 | regulation of cell cycle phase transition | 5/41 | 390/18723 | 0.00154585 | 0.012354 | 0.00698579 | CDKN1A/BUB1B/CDK1/TFDP1/CDKN2B | 5 |
| BP | GO:0001649 | osteoblast differentiation | 4/41 | 229/18723 | 0.00154811 | 0.012354 | 0.00698579 | CEBPB/CEBPA/JUND/IL6 | 4 |
| BP | GO:0045930 | negative regulation of mitotic cell cycle | 4/41 | 235/18723 | 0.00170186 | 0.0132435 | 0.00748877 | CDKN1A/BUB1B/CDK1/CDKN2B | 4 |
| BP | GO:2000116 | regulation of cysteine-type endopeptidase activity | 4/41 | 235/18723 | 0.00170186 | 0.0132435 | 0.00748877 | VEGFA/MYC/PTGS2/NGFR | 4 |
| BP | GO:0002675 | positive regulation of acute inflammatory response | 2/41 | 28/18723 | 0.00170585 | 0.0132435 | 0.00748877 | PTGS2/IL6 | 2 |
| BP | GO:0010464 | regulation of mesenchymal cell proliferation | 2/41 | 28/18723 | 0.00170585 | 0.0132435 | 0.00748877 | IRS2/MYC | 2 |
| BP | GO:0010575 | positive regulation of vascular endothelial growth factor production | 2/41 | 28/18723 | 0.00170585 | 0.0132435 | 0.00748877 | PTGS2/IL6 | 2 |
| BP | GO:0030262 | apoptotic nuclear changes | 2/41 | 28/18723 | 0.00170585 | 0.0132435 | 0.00748877 | TOP2A/IL6 | 2 |
| BP | GO:1990776 | response to angiotensin | 2/41 | 28/18723 | 0.00170585 | 0.0132435 | 0.00748877 | RELA/PTGS2 | 2 |
| BP | GO:0030072 | peptide hormone secretion | 4/41 | 236/18723 | 0.00172848 | 0.01336736 | 0.00755881 | IRS2/ARNTL/UCP2/IL6 | 4 |
| BP | GO:0014812 | muscle cell migration | 3/41 | 110/18723 | 0.00178801 | 0.01377459 | 0.00778908 | IGFBP3/PLAU/SERPINE1 | 3 |
| BP | GO:0044843 | cell cycle G1/S phase transition | 4/41 | 241/18723 | 0.00186595 | 0.01431991 | 0.00809745 | CDKN1A/CCNA2/TFDP1/CDKN2B | 4 |
| BP | GO:0002526 | acute inflammatory response | 3/41 | 112/18723 | 0.00188255 | 0.01434991 | 0.00811441 | CEBPB/PTGS2/IL6 | 3 |
| BP | GO:0001503 | ossification | 5/41 | 408/18723 | 0.00188419 | 0.01434991 | 0.00811441 | CEBPB/CEBPA/PTGS2/JUND/IL6 | 5 |
| BP | GO:0002790 | peptide secretion | 4/41 | 242/18723 | 0.00189433 | 0.0143725 | 0.00812718 | IRS2/ARNTL/UCP2/IL6 | 4 |
| BP | GO:0006921 | cellular component disassembly involved in execution phase of apoptosis | 2/41 | 30/18723 | 0.00195766 | 0.01474129 | 0.00833572 | TOP2A/IL6 | 2 |
| BP | GO:0043032 | positive regulation of macrophage activation | 2/41 | 30/18723 | 0.00195766 | 0.01474129 | 0.00833572 | CEBPA/JUND | 2 |
| BP | GO:0070663 | regulation of leukocyte proliferation | 4/41 | 245/18723 | 0.00198126 | 0.01486318 | 0.00840465 | IRS2/CDKN1A/CEBPB/IL6 | 4 |
| BP | GO:0060055 | angiogenesis involved in wound healing | 2/41 | 31/18723 | 0.00208977 | 0.01541821 | 0.0087185 | VEGFA/SERPINE1 | 2 |
| BP | GO:0071480 | cellular response to gamma radiation | 2/41 | 31/18723 | 0.00208977 | 0.01541821 | 0.0087185 | CDKN1A/EGR1 | 2 |
| BP | GO:0090183 | regulation of kidney development | 2/41 | 31/18723 | 0.00208977 | 0.01541821 | 0.0087185 | VEGFA/MYC | 2 |
| BP | GO:1902042 | negative regulation of extrinsic apoptotic signaling pathway via death domain receptors | 2/41 | 31/18723 | 0.00208977 | 0.01541821 | 0.0087185 | HMGB2/SERPINE1 | 2 |
| BP | GO:0046883 | regulation of hormone secretion | 4/41 | 249/18723 | 0.00210143 | 0.01541821 | 0.0087185 | IRS2/ARNTL/UCP2/IL6 | 4 |
| BP | GO:1901988 | negative regulation of cell cycle phase transition | 4/41 | 249/18723 | 0.00210143 | 0.01541821 | 0.0087185 | CDKN1A/BUB1B/CDK1/CDKN2B | 4 |
| BP | GO:0042060 | wound healing | 5/41 | 422/18723 | 0.00218277 | 0.01585508 | 0.00896553 | CDKN1A/VEGFA/PLAU/IL6/SERPINE1 | 5 |
| BP | GO:0072594 | establishment of protein localization to organelle | 5/41 | 422/18723 | 0.00218277 | 0.01585508 | 0.00896553 | DDIT3/CDKN1A/CDK1/HSPA8/PTGS2 | 5 |
| BP | GO:0007613 | memory | 3/41 | 118/18723 | 0.00218472 | 0.01585508 | 0.00896553 | CEBPB/NGF/PTGS2 | 3 |
| BP | GO:0010951 | negative regulation of endopeptidase activity | 4/41 | 252/18723 | 0.00219481 | 0.01587079 | 0.00897442 | VEGFA/NGF/PTGS2/SERPINE1 | 4 |
| BP | GO:0034612 | response to tumor necrosis factor | 4/41 | 253/18723 | 0.00222657 | 0.01599595 | 0.00904519 | PCK1/RELA/CEBPA/PTGS2 | 4 |
| BP | GO:0048608 | reproductive structure development | 5/41 | 424/18723 | 0.00222809 | 0.01599595 | 0.00904519 | VEGFA/HMGB2/CEBPB/CEBPA/PTGS2 | 5 |
| BP | GO:0061458 | reproductive system development | 5/41 | 427/18723 | 0.00229737 | 0.01643438 | 0.00929311 | VEGFA/HMGB2/CEBPB/CEBPA/PTGS2 | 5 |
| BP | GO:0042752 | regulation of circadian rhythm | 3/41 | 121/18723 | 0.00234646 | 0.01672585 | 0.00945792 | TOP2A/ARNTL/CDK1 | 3 |
| BP | GO:0032633 | interleukin-4 production | 2/41 | 33/18723 | 0.00236634 | 0.01674833 | 0.00947064 | DDIT3/CEBPB | 2 |
| BP | GO:0032673 | regulation of interleukin-4 production | 2/41 | 33/18723 | 0.00236634 | 0.01674833 | 0.00947064 | DDIT3/CEBPB | 2 |
| BP | GO:0002688 | regulation of leukocyte chemotaxis | 3/41 | 122/18723 | 0.00240198 | 0.01694073 | 0.00957943 | VEGFA/IL6/SERPINE1 | 3 |
| BP | GO:0006260 | DNA replication | 4/41 | 260/18723 | 0.0024578 | 0.01727361 | 0.00976767 | JUN/CCNA2/PCNA/CDK1 | 4 |
| BP | GO:0007095 | mitotic G2 DNA damage checkpoint signaling | 2/41 | 34/18723 | 0.00251076 | 0.01752282 | 0.00990859 | CDKN1A/CDK1 | 2 |
| BP | GO:0048536 | spleen development | 2/41 | 34/18723 | 0.00251076 | 0.01752282 | 0.00990859 | NFKB2/CDKN2B | 2 |
| BP | GO:0010466 | negative regulation of peptidase activity | 4/41 | 262/18723 | 0.0025268 | 0.01757354 | 0.00993726 | VEGFA/NGF/PTGS2/SERPINE1 | 4 |
| BP | GO:0015833 | peptide transport | 4/41 | 264/18723 | 0.00259712 | 0.0180001 | 0.01017848 | IRS2/ARNTL/UCP2/IL6 | 4 |
| BP | GO:0051090 | regulation of DNA-binding transcription factor activity | 5/41 | 440/18723 | 0.0026159 | 0.01806772 | 0.01021671 | DDIT3/VEGFA/TFDP1/RELA/IL6 | 5 |
| BP | GO:0045454 | cell redox homeostasis | 2/41 | 35/18723 | 0.00265924 | 0.01824132 | 0.01031487 | DDIT3/PRDX1 | 2 |
| BP | GO:0090050 | positive regulation of cell migration involved in sprouting angiogenesis | 2/41 | 35/18723 | 0.00265924 | 0.01824132 | 0.01031487 | VEGFA/PTGS2 | 2 |
| BP | GO:0001894 | tissue homeostasis | 4/41 | 268/18723 | 0.00274179 | 0.01867958 | 0.0105627 | VEGFA/PRDX1/PTGS2/IL6 | 4 |
| BP | GO:0050708 | regulation of protein secretion | 4/41 | 268/18723 | 0.00274179 | 0.01867958 | 0.0105627 | IRS2/ARNTL/UCP2/IL6 | 4 |
| BP | GO:0001952 | regulation of cell-matrix adhesion | 3/41 | 128/18723 | 0.00275225 | 0.01868728 | 0.01056705 | VEGFA/PLAU/SERPINE1 | 3 |
| BP | GO:0051385 | response to mineralocorticoid | 2/41 | 36/18723 | 0.00281178 | 0.018911 | 0.01069356 | CDKN1A/FOS | 2 |
| BP | GO:0007093 | mitotic cell cycle checkpoint signaling | 3/41 | 129/18723 | 0.00281352 | 0.018911 | 0.01069356 | CDKN1A/BUB1B/CDK1 | 3 |
| BP | GO:0034101 | erythrocyte homeostasis | 3/41 | 129/18723 | 0.00281352 | 0.018911 | 0.01069356 | VEGFA/HMGB2/PRDX1 | 3 |
| BP | GO:0022407 | regulation of cell-cell adhesion | 5/41 | 448/18723 | 0.00282725 | 0.01893975 | 0.01070981 | VEGFA/CEBPB/PCK1/RELA/IL6 | 5 |
| BP | GO:0048872 | homeostasis of number of cells | 4/41 | 272/18723 | 0.00289191 | 0.01924415 | 0.01088194 | VEGFA/HMGB2/PRDX1/IL6 | 4 |
| BP | GO:0051962 | positive regulation of nervous system development | 4/41 | 272/18723 | 0.00289191 | 0.01924415 | 0.01088194 | VEGFA/NGF/RELA/IL6 | 4 |
| BP | GO:0032570 | response to progesterone | 2/41 | 37/18723 | 0.00296834 | 0.01968737 | 0.01113257 | FOS/RELA | 2 |
| BP | GO:0010565 | regulation of cellular ketone metabolic process | 3/41 | 133/18723 | 0.00306703 | 0.02027477 | 0.01146472 | IRS2/EGR1/PTGS2 | 3 |
| BP | GO:0007346 | regulation of mitotic cell cycle | 5/41 | 457/18723 | 0.00307959 | 0.02029085 | 0.01147382 | CDKN1A/BUB1B/CDK1/TFDP1/CDKN2B | 5 |
| BP | GO:0002687 | positive regulation of leukocyte migration | 3/41 | 135/18723 | 0.00319888 | 0.02100774 | 0.0118792 | VEGFA/IL6/SERPINE1 | 3 |
| BP | GO:1900180 | regulation of protein localization to nucleus | 3/41 | 136/18723 | 0.0032661 | 0.0212802 | 0.01203326 | CDK1/PTGS2/NGFR | 3 |
| BP | GO:0010463 | mesenchymal cell proliferation | 2/41 | 39/18723 | 0.00329349 | 0.0212802 | 0.01203326 | IRS2/MYC | 2 |
| BP | GO:0016572 | histone phosphorylation | 2/41 | 39/18723 | 0.00329349 | 0.0212802 | 0.01203326 | CCNA2/CDK1 | 2 |
| BP | GO:0045923 | positive regulation of fatty acid metabolic process | 2/41 | 39/18723 | 0.00329349 | 0.0212802 | 0.01203326 | IRS2/PTGS2 | 2 |
| BP | GO:0071548 | response to dexamethasone | 2/41 | 39/18723 | 0.00329349 | 0.0212802 | 0.01203326 | PCK1/PCNA | 2 |
| BP | GO:0030879 | mammary gland development | 3/41 | 137/18723 | 0.00333418 | 0.021405 | 0.01210383 | IRS2/VEGFA/CEBPB | 3 |
| BP | GO:0050671 | positive regulation of lymphocyte proliferation | 3/41 | 137/18723 | 0.00333418 | 0.021405 | 0.01210383 | IRS2/CDKN1A/IL6 | 3 |
| BP | GO:0032946 | positive regulation of mononuclear cell proliferation | 3/41 | 138/18723 | 0.00340313 | 0.02177783 | 0.01231466 | IRS2/CDKN1A/IL6 | 3 |
| BP | GO:0014037 | Schwann cell differentiation | 2/41 | 40/18723 | 0.00346204 | 0.02187529 | 0.01236977 | CDK1/RELA | 2 |
| BP | GO:0071276 | cellular response to cadmium ion | 2/41 | 40/18723 | 0.00346204 | 0.02187529 | 0.01236977 | JUN/FOS | 2 |
| BP | GO:0150077 | regulation of neuroinflammatory response | 2/41 | 40/18723 | 0.00346204 | 0.02187529 | 0.01236977 | PTGS2/IL6 | 2 |
| BP | GO:1904994 | regulation of leukocyte adhesion to vascular endothelial cell | 2/41 | 40/18723 | 0.00346204 | 0.02187529 | 0.01236977 | RELA/IL6 | 2 |
| BP | GO:0046651 | lymphocyte proliferation | 4/41 | 288/18723 | 0.00354894 | 0.02228377 | 0.01260075 | IRS2/CDKN1A/CEBPB/IL6 | 4 |
| BP | GO:0097193 | intrinsic apoptotic signaling pathway | 4/41 | 288/18723 | 0.00354894 | 0.02228377 | 0.01260075 | DDIT3/CDKN1A/CEBPB/PTGS2 | 4 |
| BP | GO:0032355 | response to estradiol | 3/41 | 141/18723 | 0.00361521 | 0.02255784 | 0.01275573 | CCNA2/PCNA/PTGS2 | 3 |
| BP | GO:0050921 | positive regulation of chemotaxis | 3/41 | 141/18723 | 0.00361521 | 0.02255784 | 0.01275573 | VEGFA/IL6/SERPINE1 | 3 |
| BP | GO:0042307 | positive regulation of protein import into nucleus | 2/41 | 41/18723 | 0.00363455 | 0.02255784 | 0.01275573 | CDK1/PTGS2 | 2 |
| BP | GO:0034504 | protein localization to nucleus | 4/41 | 290/18723 | 0.00363764 | 0.02255784 | 0.01275573 | CDKN1A/CDK1/PTGS2/NGFR | 4 |
| BP | GO:0032943 | mononuclear cell proliferation | 4/41 | 291/18723 | 0.00368255 | 0.02265761 | 0.01281215 | IRS2/CDKN1A/CEBPB/IL6 | 4 |
| BP | GO:0072006 | nephron development | 3/41 | 142/18723 | 0.00368766 | 0.02265761 | 0.01281215 | VEGFA/EGR1/MYC | 3 |
| BP | GO:2000045 | regulation of G1/S transition of mitotic cell cycle | 3/41 | 142/18723 | 0.00368766 | 0.02265761 | 0.01281215 | CDKN1A/TFDP1/CDKN2B | 3 |
| BP | GO:0038061 | NIK/NF-kappaB signaling | 3/41 | 143/18723 | 0.003761 | 0.02303754 | 0.01302698 | PRDX1/NFKB2/RELA | 3 |
| BP | GO:0014912 | negative regulation of smooth muscle cell migration | 2/41 | 42/18723 | 0.00381101 | 0.0231384 | 0.01308401 | IGFBP3/SERPINE1 | 2 |
| BP | GO:0030890 | positive regulation of B cell proliferation | 2/41 | 42/18723 | 0.00381101 | 0.0231384 | 0.01308401 | IRS2/CDKN1A | 2 |
| BP | GO:0010948 | negative regulation of cell cycle process | 4/41 | 294/18723 | 0.00381954 | 0.0231384 | 0.01308401 | CDKN1A/BUB1B/CDK1/CDKN2B | 4 |
| BP | GO:0001890 | placenta development | 3/41 | 144/18723 | 0.00383522 | 0.0231384 | 0.01308401 | CEBPB/CEBPA/PTGS2 | 3 |
| BP | GO:0007612 | learning | 3/41 | 144/18723 | 0.00383522 | 0.0231384 | 0.01308401 | JUN/FOS/PTGS2 | 3 |
| BP | GO:0046879 | hormone secretion | 4/41 | 295/18723 | 0.00386597 | 0.02325387 | 0.01314931 | IRS2/ARNTL/UCP2/IL6 | 4 |
| BP | GO:0014002 | astrocyte development | 2/41 | 43/18723 | 0.00399139 | 0.02386494 | 0.01349485 | C1QA/IL6 | 2 |
| BP | GO:1904591 | positive regulation of protein import | 2/41 | 43/18723 | 0.00399139 | 0.02386494 | 0.01349485 | CDK1/PTGS2 | 2 |
| BP | GO:0010720 | positive regulation of cell development | 4/41 | 298/18723 | 0.00400755 | 0.02389026 | 0.01350917 | VEGFA/NGF/RELA/IL6 | 4 |
| BP | GO:0010212 | response to ionizing radiation | 3/41 | 148/18723 | 0.00414107 | 0.02438452 | 0.01378865 | CDKN1A/EGR1/MYC | 3 |
| BP | GO:0042063 | gliogenesis | 4/41 | 301/18723 | 0.00415262 | 0.02438452 | 0.01378865 | CDK1/RELA/C1QA/IL6 | 4 |
| BP | GO:0042886 | amide transport | 4/41 | 301/18723 | 0.00415262 | 0.02438452 | 0.01378865 | IRS2/ARNTL/UCP2/IL6 | 4 |
| BP | GO:0002066 | columnar/cuboidal epithelial cell development | 2/41 | 44/18723 | 0.00417568 | 0.02438452 | 0.01378865 | CDKN1A/ARNTL | 2 |
| BP | GO:0032620 | interleukin-17 production | 2/41 | 44/18723 | 0.00417568 | 0.02438452 | 0.01378865 | DDIT3/IL6 | 2 |
| BP | GO:0032660 | regulation of interleukin-17 production | 2/41 | 44/18723 | 0.00417568 | 0.02438452 | 0.01378865 | DDIT3/IL6 | 2 |
| BP | GO:0150076 | neuroinflammatory response | 2/41 | 44/18723 | 0.00417568 | 0.02438452 | 0.01378865 | PTGS2/IL6 | 2 |
| BP | GO:0070665 | positive regulation of leukocyte proliferation | 3/41 | 150/18723 | 0.00429941 | 0.02503404 | 0.01415594 | IRS2/CDKN1A/IL6 | 3 |
| BP | GO:1904646 | cellular response to amyloid-beta | 2/41 | 45/18723 | 0.00436386 | 0.02528247 | 0.01429642 | CACNA1A/NGFR | 2 |
| BP | GO:0071333 | cellular response to glucose stimulus | 3/41 | 151/18723 | 0.00437994 | 0.02528247 | 0.01429642 | IRS2/PCK1/UCP2 | 3 |
| BP | GO:2001236 | regulation of extrinsic apoptotic signaling pathway | 3/41 | 151/18723 | 0.00437994 | 0.02528247 | 0.01429642 | HMGB2/RELA/SERPINE1 | 3 |
| BP | GO:0009914 | hormone transport | 4/41 | 306/18723 | 0.00440226 | 0.0253383 | 0.01432799 | IRS2/ARNTL/UCP2/IL6 | 4 |
| BP | GO:0050871 | positive regulation of B cell activation | 3/41 | 152/18723 | 0.00446139 | 0.02560503 | 0.01447882 | IRS2/CDKN1A/IL6 | 3 |
| BP | GO:0071331 | cellular response to hexose stimulus | 3/41 | 153/18723 | 0.00454375 | 0.02585131 | 0.01461808 | IRS2/PCK1/UCP2 | 3 |
| BP | GO:0031018 | endocrine pancreas development | 2/41 | 46/18723 | 0.00455592 | 0.02585131 | 0.01461808 | ARNTL/IL6 | 2 |
| BP | GO:0034198 | cellular response to amino acid starvation | 2/41 | 46/18723 | 0.00455592 | 0.02585131 | 0.01461808 | CDKN1A/UCP2 | 2 |
| BP | GO:0044818 | mitotic G2/M transition checkpoint | 2/41 | 46/18723 | 0.00455592 | 0.02585131 | 0.01461808 | CDKN1A/CDK1 | 2 |
| BP | GO:0060326 | cell chemotaxis | 4/41 | 310/18723 | 0.00460915 | 0.02603357 | 0.01472114 | VEGFA/HMGB2/IL6/SERPINE1 | 4 |
| BP | GO:0030856 | regulation of epithelial cell differentiation | 3/41 | 154/18723 | 0.00462704 | 0.02603357 | 0.01472114 | VEGFA/CEBPB/SERPINE1 | 3 |
| BP | GO:0071326 | cellular response to monosaccharide stimulus | 3/41 | 154/18723 | 0.00462704 | 0.02603357 | 0.01472114 | IRS2/PCK1/UCP2 | 3 |
| BP | GO:0006606 | protein import into nucleus | 3/41 | 155/18723 | 0.00471124 | 0.02643311 | 0.01494707 | CDKN1A/CDK1/PTGS2 | 3 |
| BP | GO:0001774 | microglial cell activation | 2/41 | 47/18723 | 0.00475184 | 0.02643871 | 0.01495023 | C1QA/IL6 | 2 |
| BP | GO:0120163 | negative regulation of cold-induced thermogenesis | 2/41 | 47/18723 | 0.00475184 | 0.02643871 | 0.01495023 | DDIT3/ARNTL | 2 |
| BP | GO:1903587 | regulation of blood vessel endothelial cell proliferation involved in sprouting angiogenesis | 2/41 | 47/18723 | 0.00475184 | 0.02643871 | 0.01495023 | VEGFA/NGFR | 2 |
| BP | GO:0060249 | anatomical structure homeostasis | 4/41 | 314/18723 | 0.00482252 | 0.02675764 | 0.01513058 | VEGFA/PRDX1/PTGS2/IL6 | 4 |
| BP | GO:1902107 | positive regulation of leukocyte differentiation | 3/41 | 157/18723 | 0.00488244 | 0.02694085 | 0.01523418 | JUN/FOS/PCK1 | 3 |
| BP | GO:1903708 | positive regulation of hemopoiesis | 3/41 | 157/18723 | 0.00488244 | 0.02694085 | 0.01523418 | JUN/FOS/PCK1 | 3 |
| BP | GO:0002673 | regulation of acute inflammatory response | 2/41 | 48/18723 | 0.0049516 | 0.02709849 | 0.01532332 | PTGS2/IL6 | 2 |
| BP | GO:0030261 | chromosome condensation | 2/41 | 48/18723 | 0.0049516 | 0.02709849 | 0.01532332 | TOP2A/CDK1 | 2 |
| BP | GO:0033628 | regulation of cell adhesion mediated by integrin | 2/41 | 48/18723 | 0.0049516 | 0.02709849 | 0.01532332 | PLAU/SERPINE1 | 2 |
| BP | GO:0070661 | leukocyte proliferation | 4/41 | 318/18723 | 0.00504247 | 0.0275206 | 0.01556201 | IRS2/CDKN1A/CEBPB/IL6 | 4 |
| BP | GO:0051170 | import into nucleus | 3/41 | 159/18723 | 0.00505737 | 0.02752692 | 0.01556558 | CDKN1A/CDK1/PTGS2 | 3 |
| BP | GO:0030195 | negative regulation of blood coagulation | 2/41 | 49/18723 | 0.00515518 | 0.02775758 | 0.01569601 | PLAU/SERPINE1 | 2 |
| BP | GO:0030225 | macrophage differentiation | 2/41 | 49/18723 | 0.00515518 | 0.02775758 | 0.01569601 | VEGFA/CEBPA | 2 |
| BP | GO:1902041 | regulation of extrinsic apoptotic signaling pathway via death domain receptors | 2/41 | 49/18723 | 0.00515518 | 0.02775758 | 0.01569601 | HMGB2/SERPINE1 | 2 |
| BP | GO:1990928 | response to amino acid starvation | 2/41 | 49/18723 | 0.00515518 | 0.02775758 | 0.01569601 | CDKN1A/UCP2 | 2 |
| BP | GO:0051100 | negative regulation of binding | 3/41 | 162/18723 | 0.0053268 | 0.02860475 | 0.01617506 | DDIT3/JUN/CDKN1A | 3 |
| BP | GO:1900047 | negative regulation of hemostasis | 2/41 | 50/18723 | 0.00536257 | 0.02871984 | 0.01624014 | PLAU/SERPINE1 | 2 |
| BP | GO:0071322 | cellular response to carbohydrate stimulus | 3/41 | 163/18723 | 0.00541849 | 0.02894198 | 0.01636575 | IRS2/PCK1/UCP2 | 3 |
| BP | GO:0007517 | muscle organ development | 4/41 | 327/18723 | 0.00556187 | 0.0296133 | 0.01674536 | ARNTL/FOS/ELN/EGR1 | 4 |
| BP | GO:0045661 | regulation of myoblast differentiation | 2/41 | 51/18723 | 0.00557375 | 0.0296133 | 0.01674536 | DDIT3/IGFBP3 | 2 |
| BP | GO:0048638 | regulation of developmental growth | 4/41 | 330/18723 | 0.00574268 | 0.03043012 | 0.01720724 | CDKN1A/VEGFA/CDK1/NGF | 4 |
| BP | GO:0043392 | negative regulation of DNA binding | 2/41 | 52/18723 | 0.0057887 | 0.03051192 | 0.0172535 | DDIT3/JUN | 2 |
| BP | GO:0048260 | positive regulation of receptor-mediated endocytosis | 2/41 | 52/18723 | 0.0057887 | 0.03051192 | 0.0172535 | VEGFA/SERPINE1 | 2 |
| BP | GO:0019216 | regulation of lipid metabolic process | 4/41 | 331/18723 | 0.00580381 | 0.03051192 | 0.0172535 | IRS2/PCK1/EGR1/PTGS2 | 4 |
| BP | GO:1902806 | regulation of cell cycle G1/S phase transition | 3/41 | 168/18723 | 0.00589128 | 0.03089065 | 0.01746766 | CDKN1A/TFDP1/CDKN2B | 3 |
| BP | GO:0000075 | cell cycle checkpoint signaling | 3/41 | 169/18723 | 0.00598871 | 0.03109261 | 0.01758186 | CDKN1A/BUB1B/CDK1 | 3 |
| BP | GO:0001541 | ovarian follicle development | 2/41 | 53/18723 | 0.00600741 | 0.03109261 | 0.01758186 | VEGFA/CEBPB | 2 |
| BP | GO:0007566 | embryo implantation | 2/41 | 53/18723 | 0.00600741 | 0.03109261 | 0.01758186 | VEGFA/PTGS2 | 2 |
| BP | GO:0016925 | protein sumoylation | 2/41 | 53/18723 | 0.00600741 | 0.03109261 | 0.01758186 | EGR1/RELA | 2 |
| BP | GO:0050819 | negative regulation of coagulation | 2/41 | 53/18723 | 0.00600741 | 0.03109261 | 0.01758186 | PLAU/SERPINE1 | 2 |
| BP | GO:1903037 | regulation of leukocyte cell-cell adhesion | 4/41 | 336/18723 | 0.00611605 | 0.03157332 | 0.01785369 | CEBPB/PCK1/RELA/IL6 | 4 |
| BP | GO:0046890 | regulation of lipid biosynthetic process | 3/41 | 171/18723 | 0.00618647 | 0.03185477 | 0.01801284 | PCK1/EGR1/PTGS2 | 3 |
| BP | GO:0002043 | blood vessel endothelial cell proliferation involved in sprouting angiogenesis | 2/41 | 54/18723 | 0.00622986 | 0.03199591 | 0.01809265 | VEGFA/NGFR | 2 |
| BP | GO:0048639 | positive regulation of developmental growth | 3/41 | 174/18723 | 0.0064904 | 0.03324877 | 0.0188011 | VEGFA/CDK1/NGF | 3 |
| BP | GO:0001658 | branching involved in ureteric bud morphogenesis | 2/41 | 56/18723 | 0.00668591 | 0.03398955 | 0.01921999 | VEGFA/MYC | 2 |
| BP | GO:0043388 | positive regulation of DNA binding | 2/41 | 56/18723 | 0.00668591 | 0.03398955 | 0.01921999 | HMGB2/NGF | 2 |
| BP | GO:1904645 | response to amyloid-beta | 2/41 | 56/18723 | 0.00668591 | 0.03398955 | 0.01921999 | CACNA1A/NGFR | 2 |
| BP | GO:0071466 | cellular response to xenobiotic stimulus | 3/41 | 177/18723 | 0.00680312 | 0.03449785 | 0.01950742 | PCNA/MYC/UCHL1 | 3 |
| BP | GO:0030219 | megakaryocyte differentiation | 2/41 | 57/18723 | 0.00691949 | 0.03491115 | 0.01974113 | HMGB2/CDKN2B | 2 |
| BP | GO:0061756 | leukocyte adhesion to vascular endothelial cell | 2/41 | 57/18723 | 0.00691949 | 0.03491115 | 0.01974113 | RELA/IL6 | 2 |
| BP | GO:0001936 | regulation of endothelial cell proliferation | 3/41 | 179/18723 | 0.00701652 | 0.03531179 | 0.01996767 | VEGFA/HMGB2/NGFR | 3 |
| BP | GO:0042742 | defense response to bacterium | 4/41 | 350/18723 | 0.00704962 | 0.03538946 | 0.02001159 | HMGB2/CEBPB/IL6/SERPINE1 | 4 |
| BP | GO:0045861 | negative regulation of proteolysis | 4/41 | 351/18723 | 0.00711972 | 0.03539489 | 0.02001466 | VEGFA/NGF/PTGS2/SERPINE1 | 4 |
| BP | GO:0010721 | negative regulation of cell development | 3/41 | 180/18723 | 0.0071247 | 0.03539489 | 0.02001466 | VEGFA/IL6/DLL3 | 3 |
| BP | GO:0002763 | positive regulation of myeloid leukocyte differentiation | 2/41 | 58/18723 | 0.00715673 | 0.03539489 | 0.02001466 | JUN/FOS | 2 |
| BP | GO:0002931 | response to ischemia | 2/41 | 58/18723 | 0.00715673 | 0.03539489 | 0.02001466 | EGR1/UCHL1 | 2 |
| BP | GO:0010574 | regulation of vascular endothelial growth factor production | 2/41 | 58/18723 | 0.00715673 | 0.03539489 | 0.02001466 | PTGS2/IL6 | 2 |
| BP | GO:0043525 | positive regulation of neuron apoptotic process | 2/41 | 58/18723 | 0.00715673 | 0.03539489 | 0.02001466 | DDIT3/JUN | 2 |
| BP | GO:0007178 | transmembrane receptor protein serine/threonine kinase signaling pathway | 4/41 | 355/18723 | 0.00740473 | 0.03653122 | 0.02065722 | JUN/FOS/EGR1/CDKN2B | 4 |
| BP | GO:0055001 | muscle cell development | 3/41 | 184/18723 | 0.00756737 | 0.03724187 | 0.02105907 | VEGFA/CDK1/UCHL1 | 3 |
| BP | GO:0010972 | negative regulation of G2/M transition of mitotic cell cycle | 2/41 | 60/18723 | 0.00764218 | 0.037244 | 0.02106028 | CDKN1A/CDK1 | 2 |
| BP | GO:0031638 | zymogen activation | 2/41 | 60/18723 | 0.00764218 | 0.037244 | 0.02106028 | PLAU/SERPINE1 | 2 |
| BP | GO:0042306 | regulation of protein import into nucleus | 2/41 | 60/18723 | 0.00764218 | 0.037244 | 0.02106028 | CDK1/PTGS2 | 2 |
| BP | GO:0051851 | modulation by host of symbiont process | 2/41 | 60/18723 | 0.00764218 | 0.037244 | 0.02106028 | JUN/HSPA8 | 2 |
| BP | GO:0002040 | sprouting angiogenesis | 3/41 | 185/18723 | 0.00768053 | 0.03733057 | 0.02110923 | VEGFA/PTGS2/NGFR | 3 |
| BP | GO:0009306 | protein secretion | 4/41 | 359/18723 | 0.00769722 | 0.03733057 | 0.02110923 | IRS2/ARNTL/UCP2/IL6 | 4 |
| BP | GO:0035592 | establishment of protein localization to extracellular region | 4/41 | 360/18723 | 0.00777152 | 0.03759987 | 0.02126151 | IRS2/ARNTL/UCP2/IL6 | 4 |
| BP | GO:0033044 | regulation of chromosome organization | 3/41 | 187/18723 | 0.00790986 | 0.03814143 | 0.02156774 | TOP2A/BUB1B/MYC | 3 |
| BP | GO:0051251 | positive regulation of lymphocyte activation | 4/41 | 362/18723 | 0.00792153 | 0.03814143 | 0.02156774 | IRS2/CDKN1A/PCK1/IL6 | 4 |
| BP | GO:0010573 | vascular endothelial growth factor production | 2/41 | 62/18723 | 0.00814213 | 0.03873796 | 0.02190506 | PTGS2/IL6 | 2 |
| BP | GO:0032731 | positive regulation of interleukin-1 beta production | 2/41 | 62/18723 | 0.00814213 | 0.03873796 | 0.02190506 | EGR1/IL6 | 2 |
| BP | GO:0046824 | positive regulation of nucleocytoplasmic transport | 2/41 | 62/18723 | 0.00814213 | 0.03873796 | 0.02190506 | CDK1/PTGS2 | 2 |
| BP | GO:0060675 | ureteric bud morphogenesis | 2/41 | 62/18723 | 0.00814213 | 0.03873796 | 0.02190506 | VEGFA/MYC | 2 |
| BP | GO:1902750 | negative regulation of cell cycle G2/M phase transition | 2/41 | 62/18723 | 0.00814213 | 0.03873796 | 0.02190506 | CDKN1A/CDK1 | 2 |
| BP | GO:0071692 | protein localization to extracellular region | 4/41 | 368/18723 | 0.00838305 | 0.03967027 | 0.02243225 | IRS2/ARNTL/UCP2/IL6 | 4 |
| BP | GO:0070059 | intrinsic apoptotic signaling pathway in response to endoplasmic reticulum stress | 2/41 | 63/18723 | 0.0083975 | 0.03967027 | 0.02243225 | DDIT3/CEBPB | 2 |
| BP | GO:0072171 | mesonephric tubule morphogenesis | 2/41 | 63/18723 | 0.0083975 | 0.03967027 | 0.02243225 | VEGFA/MYC | 2 |
| BP | GO:0032872 | regulation of stress-activated MAPK cascade | 3/41 | 192/18723 | 0.00850084 | 0.04006395 | 0.02265486 | VEGFA/PRDX1/MYC | 3 |
| BP | GO:0007159 | leukocyte cell-cell adhesion | 4/41 | 371/18723 | 0.00862032 | 0.04032294 | 0.02280132 | CEBPB/PCK1/RELA/IL6 | 4 |
| BP | GO:0001935 | endothelial cell proliferation | 3/41 | 193/18723 | 0.00862207 | 0.04032294 | 0.02280132 | VEGFA/HMGB2/NGFR | 3 |
| BP | GO:0030888 | regulation of B cell proliferation | 2/41 | 64/18723 | 0.00865645 | 0.04032294 | 0.02280132 | IRS2/CDKN1A | 2 |
| BP | GO:0045670 | regulation of osteoclast differentiation | 2/41 | 64/18723 | 0.00865645 | 0.04032294 | 0.02280132 | CEBPB/FOS | 2 |
| BP | GO:0070542 | response to fatty acid | 2/41 | 64/18723 | 0.00865645 | 0.04032294 | 0.02280132 | UCP2/PTGS2 | 2 |
| BP | GO:0070302 | regulation of stress-activated protein kinase signaling cascade | 3/41 | 195/18723 | 0.0088676 | 0.04121069 | 0.02330331 | VEGFA/PRDX1/MYC | 3 |
| BP | GO:0014015 | positive regulation of gliogenesis | 2/41 | 66/18723 | 0.00918501 | 0.04219627 | 0.02386063 | RELA/IL6 | 2 |
| BP | GO:0030193 | regulation of blood coagulation | 2/41 | 66/18723 | 0.00918501 | 0.04219627 | 0.02386063 | PLAU/SERPINE1 | 2 |
| BP | GO:0050918 | positive chemotaxis | 2/41 | 66/18723 | 0.00918501 | 0.04219627 | 0.02386063 | VEGFA/HMGB2 | 2 |
| BP | GO:1905953 | negative regulation of lipid localization | 2/41 | 66/18723 | 0.00918501 | 0.04219627 | 0.02386063 | IRS2/IL6 | 2 |
| BP | GO:2000573 | positive regulation of DNA biosynthetic process | 2/41 | 66/18723 | 0.00918501 | 0.04219627 | 0.02386063 | PCNA/MYC | 2 |
| BP | GO:0007179 | transforming growth factor beta receptor signaling pathway | 3/41 | 198/18723 | 0.00924357 | 0.04222025 | 0.02387419 | JUN/FOS/CDKN2B | 3 |
| BP | GO:0050864 | regulation of B cell activation | 3/41 | 198/18723 | 0.00924357 | 0.04222025 | 0.02387419 | IRS2/CDKN1A/IL6 | 3 |
| BP | GO:0050878 | regulation of body fluid levels | 4/41 | 379/18723 | 0.00927454 | 0.04222025 | 0.02387419 | VEGFA/PLAU/IL6/SERPINE1 | 4 |
| BP | GO:0051346 | negative regulation of hydrolase activity | 4/41 | 379/18723 | 0.00927454 | 0.04222025 | 0.02387419 | VEGFA/NGF/PTGS2/SERPINE1 | 4 |
| BP | GO:0006323 | DNA packaging | 3/41 | 199/18723 | 0.00937094 | 0.04256234 | 0.02406762 | HMGB2/TOP2A/CDK1 | 3 |
| BP | GO:0051054 | positive regulation of DNA metabolic process | 3/41 | 201/18723 | 0.00962878 | 0.04363448 | 0.02467388 | PCNA/MYC/IL6 | 3 |
| BP | GO:0007585 | respiratory gaseous exchange by respiratory system | 2/41 | 68/18723 | 0.00972769 | 0.04378552 | 0.02475929 | ELN/UCP3 | 2 |
| BP | GO:0042698 | ovulation cycle | 2/41 | 68/18723 | 0.00972769 | 0.04378552 | 0.02475929 | EGR1/PCNA | 2 |
| BP | GO:1900046 | regulation of hemostasis | 2/41 | 68/18723 | 0.00972769 | 0.04378552 | 0.02475929 | PLAU/SERPINE1 | 2 |
| BP | GO:0034605 | cellular response to heat | 2/41 | 69/18723 | 0.01000428 | 0.04492952 | 0.02540619 | CDKN1A/PTGS2 | 2 |
| BP | GO:0006631 | fatty acid metabolic process | 4/41 | 390/18723 | 0.01022611 | 0.04582302 | 0.02591143 | IRS2/PCK1/PTGS2/UCP3 | 4 |
| BP | GO:0002548 | monocyte chemotaxis | 2/41 | 70/18723 | 0.01028435 | 0.04598114 | 0.02600084 | IL6/SERPINE1 | 2 |
| BP | GO:0050679 | positive regulation of epithelial cell proliferation | 3/41 | 207/18723 | 0.01042712 | 0.04651565 | 0.02630309 | VEGFA/HMGB2/MYC | 3 |
| BP | GO:0032722 | positive regulation of chemokine production | 2/41 | 71/18723 | 0.01056788 | 0.04683069 | 0.02648124 | EGR1/IL6 | 2 |
| BP | GO:0050818 | regulation of coagulation | 2/41 | 71/18723 | 0.01056788 | 0.04683069 | 0.02648124 | PLAU/SERPINE1 | 2 |
| BP | GO:0072078 | nephron tubule morphogenesis | 2/41 | 71/18723 | 0.01056788 | 0.04683069 | 0.02648124 | VEGFA/MYC | 2 |
| BP | GO:0002685 | regulation of leukocyte migration | 3/41 | 210/18723 | 0.01084035 | 0.04778528 | 0.02702103 | VEGFA/IL6/SERPINE1 | 3 |
| BP | GO:0033627 | cell adhesion mediated by integrin | 2/41 | 72/18723 | 0.01085487 | 0.04778528 | 0.02702103 | PLAU/SERPINE1 | 2 |
| BP | GO:0071479 | cellular response to ionizing radiation | 2/41 | 72/18723 | 0.01085487 | 0.04778528 | 0.02702103 | CDKN1A/EGR1 | 2 |
| BP | GO:0042180 | cellular ketone metabolic process | 3/41 | 211/18723 | 0.01098018 | 0.04823091 | 0.02727302 | IRS2/EGR1/PTGS2 | 3 |
| BP | GO:0032732 | positive regulation of interleukin-1 production | 2/41 | 73/18723 | 0.01114529 | 0.0486362 | 0.02750219 | EGR1/IL6 | 2 |
| BP | GO:0035924 | cellular response to vascular endothelial growth factor stimulus | 2/41 | 73/18723 | 0.01114529 | 0.0486362 | 0.02750219 | VEGFA/RELA | 2 |
| BP | GO:0072088 | nephron epithelium morphogenesis | 2/41 | 73/18723 | 0.01114529 | 0.0486362 | 0.02750219 | VEGFA/MYC | 2 |
| BP | GO:0000082 | G1/S transition of mitotic cell cycle | 3/41 | 214/18723 | 0.01140597 | 0.04966557 | 0.02808427 | CDKN1A/TFDP1/CDKN2B | 3 |
| BP | GO:0003151 | outflow tract morphogenesis | 2/41 | 74/18723 | 0.01143913 | 0.04970188 | 0.02810481 | VEGFA/ELN | 2 |
| BP | GO:0002292 | T cell differentiation involved in immune response | 2/41 | 75/18723 | 0.01173637 | 0.05055471 | 0.02858705 | PCK1/IL6 | 2 |
| BP | GO:0008088 | axo-dendritic transport | 2/41 | 75/18723 | 0.01173637 | 0.05055471 | 0.02858705 | HSPA8/UCHL1 | 2 |
| BP | GO:0048662 | negative regulation of smooth muscle cell proliferation | 2/41 | 75/18723 | 0.01173637 | 0.05055471 | 0.02858705 | CDKN1A/IGFBP3 | 2 |
| BP | GO:0061333 | renal tubule morphogenesis | 2/41 | 75/18723 | 0.01173637 | 0.05055471 | 0.02858705 | VEGFA/MYC | 2 |
| BP | GO:0007596 | blood coagulation | 3/41 | 217/18723 | 0.01184124 | 0.050897 | 0.0287806 | PLAU/IL6/SERPINE1 | 3 |
| BP | GO:0006305 | DNA alkylation | 2/41 | 76/18723 | 0.01203699 | 0.05129807 | 0.0290074 | FOS/MYC | 2 |
| BP | GO:0006306 | DNA methylation | 2/41 | 76/18723 | 0.01203699 | 0.05129807 | 0.0290074 | FOS/MYC | 2 |
| BP | GO:0043536 | positive regulation of blood vessel endothelial cell migration | 2/41 | 76/18723 | 0.01203699 | 0.05129807 | 0.0290074 | VEGFA/PTGS2 | 2 |
| BP | GO:0072028 | nephron morphogenesis | 2/41 | 76/18723 | 0.01203699 | 0.05129807 | 0.0290074 | VEGFA/MYC | 2 |
| BP | GO:0002064 | epithelial cell development | 3/41 | 220/18723 | 0.01228601 | 0.05204 | 0.02942693 | CDKN1A/VEGFA/ARNTL | 3 |
| BP | GO:0097529 | myeloid leukocyte migration | 3/41 | 220/18723 | 0.01228601 | 0.05204 | 0.02942693 | VEGFA/IL6/SERPINE1 | 3 |
| BP | GO:0043161 | proteasome-mediated ubiquitin-dependent protein catabolic process | 4/41 | 412/18723 | 0.01231545 | 0.05204 | 0.02942693 | DDIT3/ARNTL/CEBPA/UCHL1 | 4 |
| BP | GO:0021675 | nerve development | 2/41 | 77/18723 | 0.01234099 | 0.05204 | 0.02942693 | NGF/NGFR | 2 |
| BP | GO:0031016 | pancreas development | 2/41 | 77/18723 | 0.01234099 | 0.05204 | 0.02942693 | ARNTL/IL6 | 2 |
| BP | GO:0008406 | gonad development | 3/41 | 221/18723 | 0.01243639 | 0.05222242 | 0.02953008 | VEGFA/HMGB2/CEBPB | 3 |
| BP | GO:0010810 | regulation of cell-substrate adhesion | 3/41 | 221/18723 | 0.01243639 | 0.05222242 | 0.02953008 | VEGFA/PLAU/SERPINE1 | 3 |
| BP | GO:0007599 | hemostasis | 3/41 | 222/18723 | 0.01258783 | 0.05234427 | 0.02959899 | PLAU/IL6/SERPINE1 | 3 |
| BP | GO:0050817 | coagulation | 3/41 | 222/18723 | 0.01258783 | 0.05234427 | 0.02959899 | PLAU/IL6/SERPINE1 | 3 |
| BP | GO:0016032 | viral process | 4/41 | 415/18723 | 0.01262009 | 0.05234427 | 0.02959899 | JUN/TOP2A/CDK1/HSPA8 | 4 |
| BP | GO:0034637 | cellular carbohydrate biosynthetic process | 2/41 | 78/18723 | 0.01264834 | 0.05234427 | 0.02959899 | IRS2/PCK1 | 2 |
| BP | GO:0043154 | negative regulation of cysteine-type endopeptidase activity involved in apoptotic process | 2/41 | 78/18723 | 0.01264834 | 0.05234427 | 0.02959899 | VEGFA/PTGS2 | 2 |
| BP | GO:0061045 | negative regulation of wound healing | 2/41 | 78/18723 | 0.01264834 | 0.05234427 | 0.02959899 | PLAU/SERPINE1 | 2 |
| BP | GO:0090049 | regulation of cell migration involved in sprouting angiogenesis | 2/41 | 78/18723 | 0.01264834 | 0.05234427 | 0.02959899 | VEGFA/PTGS2 | 2 |
| BP | GO:0050920 | regulation of chemotaxis | 3/41 | 223/18723 | 0.01274033 | 0.05261626 | 0.02975279 | VEGFA/IL6/SERPINE1 | 3 |
| BP | GO:0007409 | axonogenesis | 4/41 | 418/18723 | 0.01292954 | 0.05328779 | 0.03013252 | VEGFA/NGF/UCHL1/NGFR | 4 |
| BP | GO:0050772 | positive regulation of axonogenesis | 2/41 | 79/18723 | 0.01295903 | 0.05329968 | 0.03013924 | VEGFA/NGF | 2 |
| BP | GO:0009791 | post-embryonic development | 2/41 | 80/18723 | 0.01327305 | 0.05436794 | 0.03074331 | VEGFA/EFEMP1 | 2 |
| BP | GO:0071229 | cellular response to acid chemical | 2/41 | 80/18723 | 0.01327305 | 0.05436794 | 0.03074331 | VEGFA/CEBPB | 2 |
| BP | GO:0045137 | development of primary sexual characteristics | 3/41 | 227/18723 | 0.013361 | 0.05461647 | 0.03088385 | VEGFA/HMGB2/CEBPB | 3 |
| BP | GO:0001942 | hair follicle development | 2/41 | 81/18723 | 0.01359038 | 0.05510431 | 0.0311597 | RELA/NGFR | 2 |
| BP | GO:0044773 | mitotic DNA damage checkpoint signaling | 2/41 | 81/18723 | 0.01359038 | 0.05510431 | 0.0311597 | CDKN1A/CDK1 | 2 |
| BP | GO:0048708 | astrocyte differentiation | 2/41 | 81/18723 | 0.01359038 | 0.05510431 | 0.0311597 | C1QA/IL6 | 2 |
| BP | GO:0051817 | modulation of process of other organism involved in symbiotic interaction | 2/41 | 81/18723 | 0.01359038 | 0.05510431 | 0.0311597 | JUN/HSPA8 | 2 |
| BP | GO:0071356 | cellular response to tumor necrosis factor | 3/41 | 229/18723 | 0.01367773 | 0.05534646 | 0.03129663 | PCK1/RELA/CEBPA | 3 |
| BP | GO:0030595 | leukocyte chemotaxis | 3/41 | 230/18723 | 0.01383771 | 0.05583915 | 0.03157523 | VEGFA/IL6/SERPINE1 | 3 |
| BP | GO:0048568 | embryonic organ development | 4/41 | 427/18723 | 0.01388708 | 0.05583915 | 0.03157523 | VEGFA/EFEMP1/CEBPB/CEBPA | 4 |
| BP | GO:0001892 | embryonic placenta development | 2/41 | 82/18723 | 0.013911 | 0.05583915 | 0.03157523 | CEBPB/CEBPA | 2 |
| BP | GO:0060395 | SMAD protein signal transduction | 2/41 | 82/18723 | 0.013911 | 0.05583915 | 0.03157523 | JUN/FOS | 2 |
| BP | GO:0007160 | cell-matrix adhesion | 3/41 | 233/18723 | 0.01432406 | 0.05738219 | 0.03244777 | VEGFA/PLAU/SERPINE1 | 3 |
| BP | GO:0006112 | energy reserve metabolic process | 2/41 | 84/18723 | 0.01456207 | 0.05775807 | 0.03266032 | IRS2/MYC | 2 |
| BP | GO:0022404 | molting cycle process | 2/41 | 84/18723 | 0.01456207 | 0.05775807 | 0.03266032 | RELA/NGFR | 2 |
| BP | GO:0022405 | hair cycle process | 2/41 | 84/18723 | 0.01456207 | 0.05775807 | 0.03266032 | RELA/NGFR | 2 |
| BP | GO:0045445 | myoblast differentiation | 2/41 | 84/18723 | 0.01456207 | 0.05775807 | 0.03266032 | DDIT3/IGFBP3 | 2 |
| BP | GO:2000134 | negative regulation of G1/S transition of mitotic cell cycle | 2/41 | 84/18723 | 0.01456207 | 0.05775807 | 0.03266032 | CDKN1A/CDKN2B | 2 |
| BP | GO:0001656 | metanephros development | 2/41 | 85/18723 | 0.01489248 | 0.05837504 | 0.03300919 | EGR1/MYC | 2 |
| BP | GO:0044774 | mitotic DNA integrity checkpoint signaling | 2/41 | 85/18723 | 0.01489248 | 0.05837504 | 0.03300919 | CDKN1A/CDK1 | 2 |
| BP | GO:0055013 | cardiac muscle cell development | 2/41 | 85/18723 | 0.01489248 | 0.05837504 | 0.03300919 | VEGFA/CDK1 | 2 |
| BP | GO:0071277 | cellular response to calcium ion | 2/41 | 85/18723 | 0.01489248 | 0.05837504 | 0.03300919 | FOS/JUND | 2 |
| BP | GO:0097194 | execution phase of apoptosis | 2/41 | 85/18723 | 0.01489248 | 0.05837504 | 0.03300919 | TOP2A/IL6 | 2 |
| BP | GO:0098773 | skin epidermis development | 2/41 | 85/18723 | 0.01489248 | 0.05837504 | 0.03300919 | RELA/NGFR | 2 |
| BP | GO:0045785 | positive regulation of cell adhesion | 4/41 | 437/18723 | 0.015003 | 0.05869339 | 0.03318921 | VEGFA/PCK1/RELA/IL6 | 4 |
| BP | GO:2000117 | negative regulation of cysteine-type endopeptidase activity | 2/41 | 86/18723 | 0.01522613 | 0.05933452 | 0.03355175 | VEGFA/PTGS2 | 2 |
| BP | GO:2001251 | negative regulation of chromosome organization | 2/41 | 86/18723 | 1.52E-02 | 5.93E-02 | 3.36E-02 | TOP2A/BUB1B | 2 |
| BP | GO:0051403 | stress-activated MAPK cascade | 3/41 | 239/18723 | 1.53E-02 | 5.95E-02 | 3.36E-02 | VEGFA/PRDX1/MYC | 3 |
| BP | GO:1903039 | positive regulation of leukocyte cell-cell adhesion | 3/41 | 239/18723 | 1.53E-02 | 0.05949148 | 3.36E-02 | PCK1/RELA/IL6 | 3 |
| BP | GO:0032392 | DNA geometric change | 2/41 | 90/18723 | 1.66E-02 | 0.0640372 | 3.62E-02 | HMGB2/TOP2A | 2 |
| BP | GO:0042475 | odontogenesis of dentin-containing tooth | 2/41 | 90/18723 | 1.66E-02 | 0.0640372 | 3.62E-02 | NGFR/SERPINE1 | 2 |
| BP | GO:1904705 | regulation of vascular associated smooth muscle cell proliferation | 2/41 | 90/18723 | 1.66E-02 | 0.0640372 | 3.62E-02 | JUN/CDKN1A | 2 |
| BP | GO:0031098 | stress-activated protein kinase signaling cascade | 3/41 | 247/18723 | 1.67E-02 | 0.06441181 | 0.03642279 | VEGFA/PRDX1/MYC | 3 |
| BP | GO:0001657 | ureteric bud development | 2/41 | 91/18723 | 1.69E-02 | 0.06476249 | 0.03662109 | VEGFA/MYC | 2 |
| BP | GO:0055006 | cardiac cell development | 2/41 | 91/18723 | 1.69E-02 | 0.06476249 | 0.03662109 | VEGFA/CDK1 | 2 |
| BP | GO:0072080 | nephron tubule development | 2/41 | 91/18723 | 1.69E-02 | 0.06476249 | 0.03662109 | VEGFA/MYC | 2 |
| BP | GO:1990874 | vascular associated smooth muscle cell proliferation | 2/41 | 91/18723 | 1.69E-02 | 0.06476249 | 0.03662109 | JUN/CDKN1A | 2 |
| BP | GO:0071560 | cellular response to transforming growth factor beta stimulus | 3/41 | 250/18723 | 1.73E-02 | 0.06560999 | 0.03710033 | JUN/FOS/CDKN2B | 3 |
| BP | GO:0034502 | protein localization to chromosome | 2/41 | 92/18723 | 1.73E-02 | 0.06560999 | 0.03710033 | BUB1B/CDK1 | 2 |
| BP | GO:0072163 | mesonephric epithelium development | 2/41 | 92/18723 | 1.73E-02 | 0.06560999 | 0.03710033 | VEGFA/MYC | 2 |
| BP | GO:0072164 | mesonephric tubule development | 2/41 | 92/18723 | 1.73E-02 | 0.06560999 | 0.03710033 | VEGFA/MYC | 2 |
| BP | GO:0019217 | regulation of fatty acid metabolic process | 2/41 | 93/18723 | 0.01765096 | 0.06620764 | 0.03743828 | IRS2/PTGS2 | 2 |
| BP | GO:0033273 | response to vitamin | 2/41 | 93/18723 | 0.01765096 | 0.06620764 | 0.03743828 | RELA/PTGS2 | 2 |
| BP | GO:0045132 | meiotic chromosome segregation | 2/41 | 93/18723 | 0.01765096 | 0.06620764 | 0.03743828 | TOP2A/BUB1B | 2 |
| BP | GO:0060993 | kidney morphogenesis | 2/41 | 93/18723 | 0.01765096 | 0.06620764 | 0.03743828 | VEGFA/MYC | 2 |
| BP | GO:1901992 | positive regulation of mitotic cell cycle phase transition | 2/41 | 93/18723 | 0.01765096 | 0.06620764 | 0.03743828 | CDK1/TFDP1 | 2 |
| BP | GO:1902807 | negative regulation of cell cycle G1/S phase transition | 2/41 | 93/18723 | 0.01765096 | 0.06620764 | 0.03743828 | CDKN1A/CDKN2B | 2 |
| BP | GO:0060828 | regulation of canonical Wnt signaling pathway | 3/41 | 253/18723 | 0.0178147 | 0.06655705 | 0.03763586 | DDIT3/ARNTL/EGR1 | 3 |
| BP | GO:0097305 | response to alcohol | 3/41 | 253/18723 | 0.0178147 | 0.06655705 | 0.03763586 | CDKN1A/FOS/CDK1 | 3 |
| BP | GO:0002042 | cell migration involved in sprouting angiogenesis | 2/41 | 94/18723 | 0.01800995 | 0.06655705 | 0.03763586 | VEGFA/PTGS2 | 2 |
| BP | GO:0010389 | regulation of G2/M transition of mitotic cell cycle | 2/41 | 94/18723 | 0.01800995 | 0.06655705 | 0.03763586 | CDKN1A/CDK1 | 2 |
| BP | GO:0030316 | osteoclast differentiation | 2/41 | 94/18723 | 0.01800995 | 0.06655705 | 0.03763586 | CEBPB/FOS | 2 |
| BP | GO:0051702 | biological process involved in interaction with symbiont | 2/41 | 94/18723 | 0.01800995 | 0.06655705 | 0.03763586 | JUN/HSPA8 | 2 |
| BP | GO:0061326 | renal tubule development | 2/41 | 94/18723 | 0.01800995 | 0.06655705 | 0.03763586 | VEGFA/MYC | 2 |
| BP | GO:1903035 | negative regulation of response to wounding | 2/41 | 94/18723 | 0.01800995 | 0.06655705 | 0.03763586 | PLAU/SERPINE1 | 2 |
| BP | GO:0023061 | signal release | 4/41 | 463/18723 | 0.0181668 | 0.06701306 | 0.03789371 | IRS2/ARNTL/UCP2/IL6 | 4 |
| BP | GO:0002532 | production of molecular mediator involved in inflammatory response | 2/41 | 95/18723 | 0.01837203 | 0.0672882 | 0.0380493 | IL6/SERPINE1 | 2 |
| BP | GO:0008585 | female gonad development | 2/41 | 95/18723 | 0.01837203 | 0.0672882 | 0.0380493 | VEGFA/CEBPB | 2 |
| BP | GO:0034976 | response to endoplasmic reticulum stress | 3/41 | 256/18723 | 0.01837576 | 0.0672882 | 0.0380493 | DDIT3/JUN/CEBPB | 3 |
| BP | GO:0071559 | response to transforming growth factor beta | 3/41 | 256/18723 | 0.01837576 | 0.0672882 | 0.0380493 | JUN/FOS/CDKN2B | 3 |
| BP | GO:0030217 | T cell differentiation | 3/41 | 257/18723 | 0.01856496 | 0.06785696 | 0.03837091 | PCK1/EGR1/IL6 | 3 |
| BP | GO:0061564 | axon development | 4/41 | 467/18723 | 0.01868783 | 0.06786729 | 0.03837675 | VEGFA/NGF/UCHL1/NGFR | 4 |
| BP | GO:0001823 | mesonephros development | 2/41 | 96/18723 | 0.0187372 | 0.06786729 | 0.03837675 | VEGFA/MYC | 2 |
| BP | GO:0034620 | cellular response to unfolded protein | 2/41 | 96/18723 | 0.0187372 | 0.06786729 | 0.03837675 | DDIT3/HSPA8 | 2 |
| BP | GO:0051304 | chromosome separation | 2/41 | 96/18723 | 0.0187372 | 0.06786729 | 0.03837675 | TOP2A/BUB1B | 2 |
| BP | GO:1904035 | regulation of epithelial cell apoptotic process | 2/41 | 96/18723 | 0.0187372 | 0.06786729 | 0.03837675 | IL6/SERPINE1 | 2 |
| BP | GO:0045927 | positive regulation of growth | 3/41 | 259/18723 | 0.01894664 | 0.06850201 | 0.03873567 | VEGFA/CDK1/NGF | 3 |
| BP | GO:0019221 | cytokine-mediated signaling pathway | 4/41 | 472/18723 | 0.01935219 | 0.06984221 | 0.03949351 | EGR1/RELA/CEBPA/IL6 | 4 |
| BP | GO:0032642 | regulation of chemokine production | 2/41 | 98/18723 | 0.01947674 | 0.07016529 | 0.0396762 | EGR1/IL6 | 2 |
| BP | GO:0007631 | feeding behavior | 2/41 | 99/18723 | 0.01985108 | 0.07113006 | 0.04022174 | FOS/UCHL1 | 2 |
| BP | GO:0032602 | chemokine production | 2/41 | 99/18723 | 0.01985108 | 0.07113006 | 0.04022174 | EGR1/IL6 | 2 |
| BP | GO:0042100 | B cell proliferation | 2/41 | 99/18723 | 0.01985108 | 0.07113006 | 0.04022174 | IRS2/CDKN1A | 2 |
| BP | GO:0009062 | fatty acid catabolic process | 2/41 | 100/18723 | 0.02022844 | 0.07183966 | 0.040623 | IRS2/PCK1 | 2 |
| BP | GO:0044728 | DNA methylation or demethylation | 2/41 | 100/18723 | 0.02022844 | 0.07183966 | 0.040623 | FOS/MYC | 2 |
| BP | GO:0045807 | positive regulation of endocytosis | 2/41 | 100/18723 | 0.02022844 | 0.07183966 | 0.040623 | VEGFA/SERPINE1 | 2 |
| BP | GO:0046545 | development of primary female sexual characteristics | 2/41 | 100/18723 | 0.02022844 | 0.07183966 | 0.040623 | VEGFA/CEBPB | 2 |
| BP | GO:0060840 | artery development | 2/41 | 100/18723 | 0.02022844 | 0.07183966 | 0.040623 | VEGFA/NGFR | 2 |
| BP | GO:0050830 | defense response to Gram-positive bacterium | 2/41 | 101/18723 | 0.02060882 | 0.07293193 | 0.04124064 | HMGB2/IL6 | 2 |
| BP | GO:0098869 | cellular oxidant detoxification | 2/41 | 101/18723 | 0.02060882 | 0.07293193 | 0.04124064 | PRDX1/PTGS2 | 2 |
| BP | GO:1902749 | regulation of cell cycle G2/M phase transition | 2/41 | 102/18723 | 0.0209922 | 0.07301298 | 0.04128647 | CDKN1A/CDK1 | 2 |
| BP | GO:1990542 | mitochondrial transmembrane transport | 2/41 | 102/18723 | 0.0209922 | 0.07301298 | 0.04128647 | UCP2/UCP3 | 2 |
| BP | GO:0014013 | regulation of gliogenesis | 2/41 | 103/18723 | 0.02137857 | 0.07301298 | 0.04128647 | RELA/IL6 | 2 |
| BP | GO:0019233 | sensory perception of pain | 2/41 | 103/18723 | 0.02137857 | 0.07301298 | 0.04128647 | PTGS2/UCHL1 | 2 |
| BP | GO:0042110 | T cell activation | 4/41 | 487/18723 | 0.02143318 | 0.07301298 | 0.04128647 | CEBPB/PCK1/EGR1/IL6 | 4 |
| BP | GO:0000050 | urea cycle | 1/41 | 10/18723 | 0.02168883 | 0.07301298 | 0.04128647 | CEBPA | 1 |
| BP | GO:0015911 | long-chain fatty acid import across plasma membrane | 1/41 | 10/18723 | 0.02168883 | 0.07301298 | 0.04128647 | IRS2 | 1 |
| BP | GO:0031915 | positive regulation of synaptic plasticity | 1/41 | 10/18723 | 0.02168883 | 0.07301298 | 0.04128647 | PTGS2 | 1 |
| BP | GO:0032070 | regulation of deoxyribonuclease activity | 1/41 | 10/18723 | 0.02168883 | 0.07301298 | 0.04128647 | PCNA | 1 |
| BP | GO:0035907 | dorsal aorta development | 1/41 | 10/18723 | 0.02168883 | 0.07301298 | 0.04128647 | NGFR | 1 |
| BP | GO:0042487 | regulation of odontogenesis of dentin-containing tooth | 1/41 | 10/18723 | 0.02168883 | 0.07301298 | 0.04128647 | NGFR | 1 |
| BP | GO:0048262 | determination of dorsal/ventral asymmetry | 1/41 | 10/18723 | 0.02168883 | 0.07301298 | 0.04128647 | DDIT3 | 1 |
| BP | GO:0048672 | positive regulation of collateral sprouting | 1/41 | 10/18723 | 0.02168883 | 0.07301298 | 0.04128647 | NGF | 1 |
| BP | GO:0051409 | response to nitrosative stress | 1/41 | 10/18723 | 0.02168883 | 0.07301298 | 0.04128647 | DDIT3 | 1 |
| BP | GO:0051918 | negative regulation of fibrinolysis | 1/41 | 10/18723 | 0.02168883 | 0.07301298 | 0.04128647 | SERPINE1 | 1 |
| BP | GO:0060947 | cardiac vascular smooth muscle cell differentiation | 1/41 | 10/18723 | 0.02168883 | 0.07301298 | 0.04128647 | VEGFA | 1 |
| BP | GO:0061179 | negative regulation of insulin secretion involved in cellular response to glucose stimulus | 1/41 | 10/18723 | 0.02168883 | 0.07301298 | 0.04128647 | UCP2 | 1 |
| BP | GO:0070091 | glucagon secretion | 1/41 | 10/18723 | 2.17E-02 | 0.07301298 | 0.04128647 | IL6 | 1 |
| BP | GO:0070092 | regulation of glucagon secretion | 1/41 | 10/18723 | 2.17E-02 | 0.07301298 | 0.04128647 | IL6 | 1 |
| BP | GO:0070391 | response to lipoteichoic acid | 1/41 | 10/18723 | 2.17E-02 | 0.07301298 | 0.04128647 | RELA | 1 |
| BP | GO:0071223 | cellular response to lipoteichoic acid | 1/41 | 10/18723 | 2.17E-02 | 0.07301298 | 0.04128647 | RELA | 1 |
| BP | GO:0072124 | regulation of glomerular mesangial cell proliferation | 1/41 | 10/18723 | 2.17E-02 | 0.07301298 | 0.04128647 | EGR1 | 1 |
| BP | GO:0090037 | positive regulation of protein kinase C signaling | 1/41 | 10/18723 | 2.17E-02 | 0.07301298 | 0.04128647 | VEGFA | 1 |
| BP | GO:0097084 | vascular associated smooth muscle cell development | 1/41 | 10/18723 | 0.02168883 | 0.07301298 | 0.04128647 | VEGFA | 1 |
| BP | GO:1901725 | regulation of histone deacetylase activity | 1/41 | 10/18723 | 0.02168883 | 0.07301298 | 0.04128647 | VEGFA | 1 |
| BP | GO:1902237 | positive regulation of endoplasmic reticulum stress-induced intrinsic apoptotic signaling pathway | 1/41 | 10/18723 | 0.02168883 | 0.07301298 | 0.04128647 | DDIT3 | 1 |
| BP | GO:2000048 | negative regulation of cell-cell adhesion mediated by cadherin | 1/41 | 10/18723 | 0.02168883 | 0.07301298 | 0.04128647 | VEGFA | 1 |
| BP | GO:2000659 | regulation of interleukin-1-mediated signaling pathway | 1/41 | 10/18723 | 0.02168883 | 0.07301298 | 0.04128647 | IL6 | 1 |
| BP | GO:2000674 | regulation of type B pancreatic cell apoptotic process | 1/41 | 10/18723 | 0.02168883 | 0.07301298 | 0.04128647 | IL6 | 1 |
| BP | GO:0006091 | generation of precursor metabolites and energy | 4/41 | 490/18723 | 0.02186534 | 0.0733606 | 0.04148304 | IRS2/CDK1/MYC/CEBPA | 4 |
| BP | GO:0010498 | proteasomal protein catabolic process | 4/41 | 490/18723 | 0.02186534 | 0.0733606 | 0.04148304 | DDIT3/ARNTL/CEBPA/UCHL1 | 4 |
| BP | GO:2001022 | positive regulation of response to DNA damage stimulus | 2/41 | 105/18723 | 0.0221602 | 0.07422554 | 0.04197214 | PCNA/MYC | 2 |
| BP | GO:0007548 | sex differentiation | 3/41 | 276/18723 | 0.0223676 | 0.07467049 | 0.04222374 | VEGFA/HMGB2/CEBPB | 3 |
| BP | GO:1903829 | positive regulation of cellular protein localization | 3/41 | 276/18723 | 0.0223676 | 0.07467049 | 0.04222374 | CDK1/PTGS2/NGFR | 3 |
| BP | GO:0035821 | modulation of process of other organism | 2/41 | 106/18723 | 0.02255543 | 0.07492293 | 0.04236649 | JUN/HSPA8 | 2 |
| BP | GO:0046822 | regulation of nucleocytoplasmic transport | 2/41 | 106/18723 | 0.02255543 | 0.07492293 | 0.04236649 | CDK1/PTGS2 | 2 |
| BP | GO:0071887 | leukocyte apoptotic process | 2/41 | 106/18723 | 0.02255543 | 0.07492293 | 0.04236649 | IRS2/IL6 | 2 |
| BP | GO:0072009 | nephron epithelium development | 2/41 | 109/18723 | 0.02375865 | 0.07637775 | 0.04318914 | VEGFA/MYC | 2 |
| BP | GO:0001660 | fever generation | 1/41 | 11/18723 | 0.0238323 | 0.07637775 | 0.04318914 | PTGS2 | 1 |
| BP | GO:0031652 | positive regulation of heat generation | 1/41 | 11/18723 | 0.0238323 | 0.07637775 | 0.04318914 | PTGS2 | 1 |
| BP | GO:0032000 | positive regulation of fatty acid beta-oxidation | 1/41 | 11/18723 | 0.0238323 | 0.07637775 | 0.04318914 | IRS2 | 1 |
| BP | GO:0033234 | negative regulation of protein sumoylation | 1/41 | 11/18723 | 0.0238323 | 0.07637775 | 0.04318914 | RELA | 1 |
| BP | GO:0045654 | positive regulation of megakaryocyte differentiation | 1/41 | 11/18723 | 0.0238323 | 0.07637775 | 0.04318914 | HMGB2 | 1 |
| BP | GO:0045657 | positive regulation of monocyte differentiation | 1/41 | 11/18723 | 0.0238323 | 0.07637775 | 0.04318914 | JUN | 1 |
| BP | GO:0045945 | positive regulation of transcription by RNA polymerase III | 1/41 | 11/18723 | 0.0238323 | 0.07637775 | 0.04318914 | CEBPA | 1 |
| BP | GO:0055015 | ventricular cardiac muscle cell development | 1/41 | 11/18723 | 0.0238323 | 0.07637775 | 0.04318914 | CDK1 | 1 |
| BP | GO:0061635 | regulation of protein complex stability | 1/41 | 11/18723 | 0.0238323 | 0.07637775 | 0.04318914 | HSPA8 | 1 |
| BP | GO:0070341 | fat cell proliferation | 1/41 | 11/18723 | 0.0238323 | 0.07637775 | 0.04318914 | TFDP1 | 1 |
| BP | GO:0070344 | regulation of fat cell proliferation | 1/41 | 11/18723 | 0.0238323 | 0.07637775 | 0.04318914 | TFDP1 | 1 |
| BP | GO:0070601 | centromeric sister chromatid cohesion | 1/41 | 11/18723 | 0.0238323 | 0.07637775 | 0.04318914 | BUB1B | 1 |
| BP | GO:0071377 | cellular response to glucagon stimulus | 1/41 | 11/18723 | 0.0238323 | 0.07637775 | 0.04318914 | PCK1 | 1 |
| BP | GO:0071679 | commissural neuron axon guidance | 1/41 | 11/18723 | 0.0238323 | 0.07637775 | 0.04318914 | VEGFA | 1 |
| BP | GO:0072110 | glomerular mesangial cell proliferation | 1/41 | 11/18723 | 0.0238323 | 0.07637775 | 0.04318914 | EGR1 | 1 |
| BP | GO:0097050 | type B pancreatic cell apoptotic process | 1/41 | 11/18723 | 0.0238323 | 0.07637775 | 0.04318914 | IL6 | 1 |
| BP | GO:0098883 | synapse pruning | 1/41 | 11/18723 | 0.0238323 | 0.07637775 | 0.04318914 | C1QA | 1 |
| BP | GO:0106049 | regulation of cellular response to osmotic stress | 1/41 | 11/18723 | 0.0238323 | 0.07637775 | 0.04318914 | PTGS2 | 1 |
| BP | GO:1902065 | response to L-glutamate | 1/41 | 11/18723 | 0.0238323 | 0.07637775 | 0.04318914 | PCNA | 1 |
| BP | GO:1902510 | regulation of apoptotic DNA fragmentation | 1/41 | 11/18723 | 0.0238323 | 0.07637775 | 0.04318914 | IL6 | 1 |
| BP | GO:1902667 | regulation of axon guidance | 1/41 | 11/18723 | 0.0238323 | 0.07637775 | 0.04318914 | VEGFA | 1 |
| BP | GO:0022409 | positive regulation of cell-cell adhesion | 3/41 | 284/18723 | 0.0240871 | 0.07670922 | 0.04337658 | PCK1/RELA/IL6 | 3 |
| BP | GO:0009408 | response to heat | 2/41 | 110/18723 | 0.02416551 | 0.07670922 | 0.04337658 | CDKN1A/PTGS2 | 2 |
| BP | GO:0032611 | interleukin-1 beta production | 2/41 | 110/18723 | 0.02416551 | 0.07670922 | 0.04337658 | EGR1/IL6 | 2 |
| BP | GO:0032651 | regulation of interleukin-1 beta production | 2/41 | 110/18723 | 0.02416551 | 0.07670922 | 0.04337658 | EGR1/IL6 | 2 |
| BP | GO:0048259 | regulation of receptor-mediated endocytosis | 2/41 | 110/18723 | 0.02416551 | 0.07670922 | 0.04337658 | VEGFA/SERPINE1 | 2 |
| BP | GO:0061387 | regulation of extent of cell growth | 2/41 | 110/18723 | 0.02416551 | 0.07670922 | 0.04337658 | VEGFA/NGF | 2 |
| BP | GO:0001938 | positive regulation of endothelial cell proliferation | 2/41 | 111/18723 | 0.02457525 | 0.07788642 | 0.04404225 | VEGFA/HMGB2 | 2 |
| BP | GO:1901222 | regulation of NIK/NF-kappaB signaling | 2/41 | 112/18723 | 0.02498784 | 0.07906895 | 0.04471093 | PRDX1/RELA | 2 |
| BP | GO:0044403 | biological process involved in symbiotic interaction | 3/41 | 290/18723 | 0.02542282 | 0.07966353 | 0.04504715 | JUN/CDK1/HSPA8 | 3 |
| BP | GO:0071103 | DNA conformation change | 3/41 | 290/18723 | 0.02542282 | 0.07966353 | 0.04504715 | HMGB2/TOP2A/CDK1 | 3 |
| BP | GO:0060485 | mesenchyme development | 3/41 | 291/18723 | 0.02564928 | 0.07966353 | 0.04504715 | MYC/IL6/DLL3 | 3 |
| BP | GO:0002286 | T cell activation involved in immune response | 2/41 | 114/18723 | 0.02582155 | 0.07966353 | 0.04504715 | PCK1/IL6 | 2 |
| BP | GO:0046660 | female sex differentiation | 2/41 | 114/18723 | 0.02582155 | 0.07966353 | 0.04504715 | VEGFA/CEBPB | 2 |
| BP | GO:0002903 | negative regulation of B cell apoptotic process | 1/41 | 12/18723 | 0.02597119 | 0.07966353 | 0.04504715 | IRS2 | 1 |
| BP | GO:0009886 | post-embryonic animal morphogenesis | 1/41 | 12/18723 | 0.02597119 | 0.07966353 | 0.04504715 | EFEMP1 | 1 |
| BP | GO:0019627 | urea metabolic process | 1/41 | 12/18723 | 0.02597119 | 0.07966353 | 0.04504715 | CEBPA | 1 |
| BP | GO:0033629 | negative regulation of cell adhesion mediated by integrin | 1/41 | 12/18723 | 0.02597119 | 0.07966353 | 0.04504715 | SERPINE1 | 1 |
| BP | GO:0038180 | nerve growth factor signaling pathway | 1/41 | 12/18723 | 0.02597119 | 0.07966353 | 0.04504715 | NGF | 1 |
| BP | GO:0043129 | surfactant homeostasis | 1/41 | 12/18723 | 0.02597119 | 0.07966353 | 0.04504715 | VEGFA | 1 |
| BP | GO:0043380 | regulation of memory T cell differentiation | 1/41 | 12/18723 | 0.02597119 | 0.07966353 | 0.04504715 | PCK1 | 1 |
| BP | GO:0045602 | negative regulation of endothelial cell differentiation | 1/41 | 12/18723 | 0.02597119 | 0.07966353 | 0.04504715 | VEGFA | 1 |
| BP | GO:0071468 | cellular response to acidic pH | 1/41 | 12/18723 | 0.02597119 | 0.07966353 | 0.04504715 | PCK1 | 1 |
| BP | GO:0071941 | nitrogen cycle metabolic process | 1/41 | 12/18723 | 0.02597119 | 0.07966353 | 0.04504715 | CEBPA | 1 |
| BP | GO:0097201 | negative regulation of transcription from RNA polymerase II promoter in response to stress | 1/41 | 12/18723 | 0.02597119 | 0.07966353 | 0.04504715 | JUN | 1 |
| BP | GO:0150065 | regulation of deacetylase activity | 1/41 | 12/18723 | 0.02597119 | 0.07966353 | 0.04504715 | VEGFA | 1 |
| BP | GO:1903624 | regulation of DNA catabolic process | 1/41 | 12/18723 | 0.02597119 | 0.07966353 | 0.04504715 | IL6 | 1 |
| BP | GO:1903800 | positive regulation of production of miRNAs involved in gene silencing by miRNA | 1/41 | 12/18723 | 0.02597119 | 0.07966353 | 0.04504715 | IL6 | 1 |
| BP | GO:2000628 | regulation of miRNA metabolic process | 1/41 | 12/18723 | 0.02597119 | 0.07966353 | 0.04504715 | RELA | 1 |
| BP | GO:0001822 | kidney development | 3/41 | 293/18723 | 0.0261055 | 0.07995308 | 0.04521087 | VEGFA/EGR1/MYC | 3 |
| BP | GO:0000077 | DNA damage checkpoint signaling | 2/41 | 115/18723 | 0.02624264 | 0.08000609 | 0.04524085 | CDKN1A/CDK1 | 2 |
| BP | GO:0043279 | response to alkaloid | 2/41 | 115/18723 | 0.02624264 | 0.08000609 | 0.04524085 | CCNA2/RELA | 2 |
| BP | GO:1901989 | positive regulation of cell cycle phase transition | 2/41 | 115/18723 | 0.02624264 | 0.08000609 | 0.04524085 | CDK1/TFDP1 | 2 |
| BP | GO:0008286 | insulin receptor signaling pathway | 2/41 | 116/18723 | 0.02666653 | 0.08080644 | 0.04569342 | IRS2/RELA | 2 |
| BP | GO:0021782 | glial cell development | 2/41 | 116/18723 | 0.02666653 | 0.08080644 | 0.04569342 | C1QA/IL6 | 2 |
| BP | GO:0035967 | cellular response to topologically incorrect protein | 2/41 | 116/18723 | 0.02666653 | 0.08080644 | 0.04569342 | DDIT3/HSPA8 | 2 |
| BP | GO:1990748 | cellular detoxification | 2/41 | 116/18723 | 0.02666653 | 0.08080644 | 0.04569342 | PRDX1/PTGS2 | 2 |
| BP | GO:0022612 | gland morphogenesis | 2/41 | 118/18723 | 0.02752267 | 0.08266567 | 0.04674476 | CEBPB/IL6 | 2 |
| BP | GO:0006913 | nucleocytoplasmic transport | 3/41 | 301/18723 | 0.02797421 | 0.08266567 | 0.04674476 | CDKN1A/CDK1/PTGS2 | 3 |
| BP | GO:0030198 | extracellular matrix organization | 3/41 | 301/18723 | 0.02797421 | 0.08266567 | 0.04674476 | ELN/NFKB2/IL6 | 3 |
| BP | GO:0051169 | nuclear transport | 3/41 | 301/18723 | 0.02797421 | 0.08266567 | 0.04674476 | CDKN1A/CDK1/PTGS2 | 3 |
| BP | GO:0002551 | mast cell chemotaxis | 1/41 | 13/18723 | 0.0281055 | 0.08266567 | 0.04674476 | VEGFA | 1 |
| BP | GO:0006983 | ER overload response | 1/41 | 13/18723 | 0.0281055 | 0.08266567 | 0.04674476 | DDIT3 | 1 |
| BP | GO:0010755 | regulation of plasminogen activation | 1/41 | 13/18723 | 0.0281055 | 0.08266567 | 0.04674476 | SERPINE1 | 1 |
| BP | GO:0016322 | neuron remodeling | 1/41 | 13/18723 | 0.0281055 | 0.08266567 | 0.04674476 | C1QA | 1 |
| BP | GO:0031053 | primary miRNA processing | 1/41 | 13/18723 | 0.0281055 | 0.08266567 | 0.04674476 | IL6 | 1 |
| BP | GO:0031392 | regulation of prostaglandin biosynthetic process | 1/41 | 13/18723 | 0.0281055 | 0.08266567 | 0.04674476 | PTGS2 | 1 |
| BP | GO:0032352 | positive regulation of hormone metabolic process | 1/41 | 13/18723 | 0.0281055 | 0.08266567 | 0.04674476 | EGR1 | 1 |
| BP | GO:0034112 | positive regulation of homotypic cell-cell adhesion | 1/41 | 13/18723 | 0.0281055 | 0.08266567 | 0.04674476 | IL6 | 1 |
| BP | GO:0035745 | T-helper 2 cell cytokine production | 1/41 | 13/18723 | 0.0281055 | 0.08266567 | 0.04674476 | IL6 | 1 |
| BP | GO:0043379 | memory T cell differentiation | 1/41 | 13/18723 | 0.0281055 | 0.08266567 | 0.04674476 | PCK1 | 1 |
| BP | GO:0044794 | positive regulation by host of viral process | 1/41 | 13/18723 | 0.0281055 | 0.08266567 | 0.04674476 | HSPA8 | 1 |
| BP | GO:0070316 | regulation of G0 to G1 transition | 1/41 | 13/18723 | 0.0281055 | 0.08266567 | 0.04674476 | CDKN2B | 1 |
| BP | GO:1902947 | regulation of tau-protein kinase activity | 1/41 | 13/18723 | 0.0281055 | 0.08266567 | 0.04674476 | EGR1 | 1 |
| BP | GO:1903025 | regulation of RNA polymerase II regulatory region sequence-specific DNA binding | 1/41 | 13/18723 | 0.0281055 | 0.08266567 | 0.04674476 | DDIT3 | 1 |
| BP | GO:2000551 | regulation of T-helper 2 cell cytokine production | 1/41 | 13/18723 | 0.0281055 | 0.08266567 | 0.04674476 | IL6 | 1 |
| BP | GO:2001198 | regulation of dendritic cell differentiation | 1/41 | 13/18723 | 0.0281055 | 0.08266567 | 0.04674476 | CEBPB | 1 |
| BP | GO:0043062 | extracellular structure organization | 3/41 | 302/18723 | 0.02821273 | 0.08273807 | 0.04678569 | ELN/NFKB2/IL6 | 3 |
| BP | GO:0072001 | renal system development | 3/41 | 302/18723 | 0.02821273 | 0.08273807 | 0.04678569 | VEGFA/EGR1/MYC | 3 |
| BP | GO:0006304 | DNA modification | 2/41 | 120/18723 | 0.02838985 | 0.08283437 | 0.04684015 | FOS/MYC | 2 |
| BP | GO:0030218 | erythrocyte differentiation | 2/41 | 120/18723 | 0.02838985 | 0.08283437 | 0.04684015 | VEGFA/HMGB2 | 2 |
| BP | GO:0007162 | negative regulation of cell adhesion | 3/41 | 303/18723 | 0.02845234 | 0.08283437 | 0.04684015 | VEGFA/CEBPB/SERPINE1 | 3 |
| BP | GO:0051222 | positive regulation of protein transport | 3/41 | 303/18723 | 0.02845234 | 0.08283437 | 0.04684015 | IRS2/CDK1/PTGS2 | 3 |
| BP | GO:0060070 | canonical Wnt signaling pathway | 3/41 | 303/18723 | 0.02845234 | 0.08283437 | 0.04684015 | DDIT3/ARNTL/EGR1 | 3 |
| BP | GO:0045229 | external encapsulating structure organization | 3/41 | 304/18723 | 0.02869305 | 0.08341391 | 0.04716786 | ELN/NFKB2/IL6 | 3 |
| BP | GO:0045931 | positive regulation of mitotic cell cycle | 2/41 | 121/18723 | 0.02882756 | 0.08356237 | 0.04725181 | CDK1/TFDP1 | 2 |
| BP | GO:1904019 | epithelial cell apoptotic process | 2/41 | 121/18723 | 0.02882756 | 0.08356237 | 0.04725181 | IL6/SERPINE1 | 2 |
| BP | GO:0072329 | monocarboxylic acid catabolic process | 2/41 | 122/18723 | 0.02926798 | 0.08471643 | 0.0479044 | IRS2/PCK1 | 2 |
| BP | GO:0031570 | DNA integrity checkpoint signaling | 2/41 | 123/18723 | 0.02971112 | 0.08493859 | 0.04803002 | CDKN1A/CDK1 | 2 |
| BP | GO:0097237 | cellular response to toxic substance | 2/41 | 124/18723 | 0.03015695 | 0.08493859 | 0.04803002 | PRDX1/PTGS2 | 2 |
| BP | GO:0002467 | germinal center formation | 1/41 | 14/18723 | 0.03023526 | 0.08493859 | 0.04803002 | NFKB2 | 1 |
| BP | GO:0006287 | base-excision repair, gap-filling | 1/41 | 14/18723 | 0.03023526 | 0.08493859 | 0.04803002 | PCNA | 1 |
| BP | GO:0031650 | regulation of heat generation | 1/41 | 14/18723 | 0.03023526 | 0.08493859 | 0.04803002 | PTGS2 | 1 |
| BP | GO:0032966 | negative regulation of collagen biosynthetic process | 1/41 | 14/18723 | 0.03023526 | 0.08493859 | 0.04803002 | IL6 | 1 |
| BP | GO:0042921 | glucocorticoid receptor signaling pathway | 1/41 | 14/18723 | 0.03023526 | 0.08493859 | 0.04803002 | ARNTL | 1 |
| BP | GO:0043922 | negative regulation by host of viral transcription | 1/41 | 14/18723 | 0.03023526 | 0.08493859 | 0.04803002 | JUN | 1 |
| BP | GO:0045023 | G0 to G1 transition | 1/41 | 14/18723 | 0.03023526 | 0.08493859 | 0.04803002 | CDKN2B | 1 |
| BP | GO:0047484 | regulation of response to osmotic stress | 1/41 | 14/18723 | 0.03023526 | 0.08493859 | 0.04803002 | PTGS2 | 1 |
| BP | GO:0048875 | chemical homeostasis within a tissue | 1/41 | 14/18723 | 0.03023526 | 0.08493859 | 0.04803002 | VEGFA | 1 |
| BP | GO:0050872 | white fat cell differentiation | 1/41 | 14/18723 | 0.03023526 | 0.08493859 | 0.04803002 | CEBPA | 1 |
| BP | GO:0050930 | induction of positive chemotaxis | 1/41 | 14/18723 | 0.03023526 | 0.08493859 | 0.04803002 | VEGFA | 1 |
| BP | GO:0051917 | regulation of fibrinolysis | 1/41 | 14/18723 | 0.03023526 | 0.08493859 | 0.04803002 | SERPINE1 | 1 |
| BP | GO:0060576 | intestinal epithelial cell development | 1/41 | 14/18723 | 0.03023526 | 0.08493859 | 0.04803002 | CDKN1A | 1 |
| BP | GO:0060841 | venous blood vessel development | 1/41 | 14/18723 | 0.03023526 | 0.08493859 | 0.04803002 | VEGFA | 1 |
| BP | GO:0061043 | regulation of vascular wound healing | 1/41 | 14/18723 | 0.03023526 | 0.08493859 | 0.04803002 | SERPINE1 | 1 |
| BP | GO:0071285 | cellular response to lithium ion | 1/41 | 14/18723 | 0.03023526 | 0.08493859 | 0.04803002 | CEBPA | 1 |
| BP | GO:0072540 | T-helper 17 cell lineage commitment | 1/41 | 14/18723 | 0.03023526 | 0.08493859 | 0.04803002 | IL6 | 1 |
| BP | GO:0090715 | immunological memory formation process | 1/41 | 14/18723 | 0.03023526 | 0.08493859 | 0.04803002 | PCK1 | 1 |
| BP | GO:0097531 | mast cell migration | 1/41 | 14/18723 | 0.03023526 | 0.08493859 | 0.04803002 | VEGFA | 1 |
| BP | GO:0055007 | cardiac muscle cell differentiation | 2/41 | 125/18723 | 0.03060546 | 0.0857381 | 0.04848212 | VEGFA/CDK1 | 2 |
| BP | GO:2000027 | regulation of animal organ morphogenesis | 2/41 | 125/18723 | 0.03060546 | 0.0857381 | 0.04848212 | VEGFA/NGFR | 2 |
| BP | GO:2001235 | positive regulation of apoptotic signaling pathway | 2/41 | 126/18723 | 0.03105664 | 0.08688053 | 0.04912812 | DDIT3/NGFR | 2 |
| BP | GO:0035270 | endocrine system development | 2/41 | 127/18723 | 0.03151048 | 0.08743859 | 0.04944369 | ARNTL/IL6 | 2 |
| BP | GO:0019058 | viral life cycle | 3/41 | 317/18723 | 0.03192171 | 0.08743859 | 0.04944369 | TOP2A/CDK1/HSPA8 | 3 |
| BP | GO:0032612 | interleukin-1 production | 2/41 | 128/18723 | 0.03196696 | 0.08743859 | 0.04944369 | EGR1/IL6 | 2 |
| BP | GO:0032652 | regulation of interleukin-1 production | 2/41 | 128/18723 | 0.03196696 | 0.08743859 | 0.04944369 | EGR1/IL6 | 2 |
| BP | GO:0015980 | energy derivation by oxidation of organic compounds | 3/41 | 318/18723 | 0.0321777 | 0.08743859 | 0.04944369 | IRS2/CDK1/MYC | 3 |
| BP | GO:0002070 | epithelial cell maturation | 1/41 | 15/18723 | 0.03236046 | 0.08743859 | 0.04944369 | CDKN1A | 1 |
| BP | GO:0006271 | DNA strand elongation involved in DNA replication | 1/41 | 15/18723 | 0.03236046 | 0.08743859 | 0.04944369 | PCNA | 1 |
| BP | GO:0010713 | negative regulation of collagen metabolic process | 1/41 | 15/18723 | 0.03236046 | 0.08743859 | 0.04944369 | IL6 | 1 |
| BP | GO:0015671 | oxygen transport | 1/41 | 15/18723 | 0.03236046 | 0.08743859 | 0.04944369 | MYC | 1 |
| BP | GO:0031958 | corticosteroid receptor signaling pathway | 1/41 | 15/18723 | 0.03236046 | 0.08743859 | 0.04944369 | ARNTL | 1 |
| BP | GO:0045475 | locomotor rhythm | 1/41 | 15/18723 | 0.03236046 | 0.08743859 | 0.04944369 | EGR1 | 1 |
| BP | GO:0045725 | positive regulation of glycogen biosynthetic process | 1/41 | 15/18723 | 0.03236046 | 0.08743859 | 0.04944369 | IRS2 | 1 |
| BP | GO:0045986 | negative regulation of smooth muscle contraction | 1/41 | 15/18723 | 0.03236046 | 0.08743859 | 0.04944369 | PTGS2 | 1 |
| BP | GO:0046321 | positive regulation of fatty acid oxidation | 1/41 | 15/18723 | 0.03236046 | 0.08743859 | 0.04944369 | IRS2 | 1 |
| BP | GO:0046459 | short-chain fatty acid metabolic process | 1/41 | 15/18723 | 0.03236046 | 0.08743859 | 0.04944369 | PCK1 | 1 |
| BP | GO:0048308 | organelle inheritance | 1/41 | 15/18723 | 0.03236046 | 0.08743859 | 0.04944369 | CDK1 | 1 |
| BP | GO:0048313 | Golgi inheritance | 1/41 | 15/18723 | 0.03236046 | 0.08743859 | 0.04944369 | CDK1 | 1 |
| BP | GO:0051775 | response to redox state | 1/41 | 15/18723 | 0.03236046 | 0.08743859 | 0.04944369 | ARNTL | 1 |
| BP | GO:0055012 | ventricular cardiac muscle cell differentiation | 1/41 | 15/18723 | 0.03236046 | 0.08743859 | 0.04944369 | CDK1 | 1 |
| BP | GO:0072075 | metanephric mesenchyme development | 1/41 | 15/18723 | 0.03236046 | 0.08743859 | 0.04944369 | MYC | 1 |
| BP | GO:0072224 | metanephric glomerulus development | 1/41 | 15/18723 | 0.03236046 | 0.08743859 | 0.04944369 | EGR1 | 1 |
| BP | GO:1903799 | negative regulation of production of miRNAs involved in gene silencing by miRNA | 1/41 | 15/18723 | 0.03236046 | 0.08743859 | 0.04944369 | IL6 | 1 |
| BP | GO:2001028 | positive regulation of endothelial cell chemotaxis | 1/41 | 15/18723 | 0.03236046 | 0.08743859 | 0.04944369 | VEGFA | 1 |
| BP | GO:2001279 | regulation of unsaturated fatty acid biosynthetic process | 1/41 | 15/18723 | 0.03236046 | 0.08743859 | 0.04944369 | PTGS2 | 1 |
| BP | GO:0007498 | mesoderm development | 2/41 | 129/18723 | 0.03242607 | 0.08743859 | 0.04944369 | VEGFA/DLL3 | 2 |
| BP | GO:0043280 | positive regulation of cysteine-type endopeptidase activity involved in apoptotic process | 2/41 | 129/18723 | 0.03242607 | 0.08743859 | 0.04944369 | MYC/NGFR | 2 |
| BP | GO:1904951 | positive regulation of establishment of protein localization | 3/41 | 319/18723 | 0.03243478 | 0.08743859 | 0.04944369 | IRS2/CDK1/PTGS2 | 3 |
| BP | GO:0042476 | odontogenesis | 2/41 | 130/18723 | 0.0328878 | 0.08842182 | 0.04999967 | NGFR/SERPINE1 | 2 |
| BP | GO:0043467 | regulation of generation of precursor metabolites and energy | 2/41 | 130/18723 | 0.0328878 | 0.08842182 | 0.04999967 | IRS2/CDK1 | 2 |
| BP | GO:0000723 | telomere maintenance | 2/41 | 131/18723 | 0.03335213 | 0.08943013 | 0.05056984 | PCNA/MYC | 2 |
| BP | GO:0019079 | viral genome replication | 2/41 | 131/18723 | 0.03335213 | 0.08943013 | 0.05056984 | TOP2A/HSPA8 | 2 |
| BP | GO:0008544 | epidermis development | 3/41 | 324/18723 | 0.03373649 | 0.09033984 | 0.05108425 | TFDP1/RELA/NGFR | 3 |
| BP | GO:0006997 | nucleus organization | 2/41 | 133/18723 | 0.03428854 | 0.09040008 | 0.05111832 | HMGB2/CDK1 | 2 |
| BP | GO:0010595 | positive regulation of endothelial cell migration | 2/41 | 133/18723 | 0.03428854 | 0.09040008 | 0.05111832 | VEGFA/PTGS2 | 2 |
| BP | GO:0002830 | positive regulation of type 2 immune response | 1/41 | 16/18723 | 0.03448111 | 0.09040008 | 0.05111832 | IL6 | 1 |
| BP | GO:0006266 | DNA ligation | 1/41 | 16/18723 | 0.03448111 | 0.09040008 | 0.05111832 | TOP2A | 1 |
| BP | GO:0006978 | DNA damage response, signal transduction by p53 class mediator resulting in transcription of p21 class mediator | 1/41 | 16/18723 | 0.03448111 | 0.09040008 | 0.05111832 | CDKN1A | 1 |
| BP | GO:0042481 | regulation of odontogenesis | 1/41 | 16/18723 | 0.03448111 | 0.09040008 | 0.05111832 | NGFR | 1 |
| BP | GO:0043923 | positive regulation by host of viral transcription | 1/41 | 16/18723 | 0.03448111 | 0.09040008 | 0.05111832 | JUN | 1 |
| BP | GO:0045779 | negative regulation of bone resorption | 1/41 | 16/18723 | 0.03448111 | 0.09040008 | 0.05111832 | IL6 | 1 |
| BP | GO:0050862 | positive regulation of T cell receptor signaling pathway | 1/41 | 16/18723 | 0.03448111 | 0.09040008 | 0.05111832 | RELA | 1 |
| BP | GO:0061684 | chaperone-mediated autophagy | 1/41 | 16/18723 | 0.03448111 | 0.09040008 | 0.05111832 | HSPA8 | 1 |
| BP | GO:0070365 | hepatocyte differentiation | 1/41 | 16/18723 | 0.03448111 | 0.09040008 | 0.05111832 | PCK1 | 1 |
| BP | GO:0070431 | nucleotide-binding oligomerization domain containing 2 signaling pathway | 1/41 | 16/18723 | 0.03448111 | 0.09040008 | 0.05111832 | RELA | 1 |
| BP | GO:0071732 | cellular response to nitric oxide | 1/41 | 16/18723 | 0.03448111 | 0.09040008 | 0.05111832 | CCNA2 | 1 |
| BP | GO:0072109 | glomerular mesangium development | 1/41 | 16/18723 | 0.03448111 | 0.09040008 | 0.05111832 | EGR1 | 1 |
| BP | GO:0090336 | positive regulation of brown fat cell differentiation | 1/41 | 16/18723 | 0.03448111 | 0.09040008 | 0.05111832 | PTGS2 | 1 |
| BP | GO:1901163 | regulation of trophoblast cell migration | 1/41 | 16/18723 | 0.03448111 | 0.09040008 | 0.05111832 | VEGFA | 1 |
| BP | GO:0061041 | regulation of wound healing | 2/41 | 134/18723 | 0.0347606 | 0.0909911 | 0.05145252 | PLAU/SERPINE1 | 2 |
| BP | GO:0030111 | regulation of Wnt signaling pathway | 3/41 | 328/18723 | 0.0347974 | 0.0909911 | 0.05145252 | DDIT3/ARNTL/EGR1 | 3 |
| BP | GO:0050863 | regulation of T cell activation | 3/41 | 329/18723 | 0.03506533 | 0.09157217 | 0.05178109 | CEBPB/PCK1/IL6 | 3 |
| BP | GO:0003002 | regionalization | 3/41 | 331/18723 | 0.03560444 | 0.09209077 | 0.05207435 | DDIT3/C1QA/DLL3 | 3 |
| BP | GO:0072073 | kidney epithelium development | 2/41 | 136/18723 | 0.03571236 | 0.09209077 | 0.05207435 | VEGFA/MYC | 2 |
| BP | GO:0006986 | response to unfolded protein | 2/41 | 137/18723 | 0.03619204 | 0.09209077 | 0.05207435 | DDIT3/HSPA8 | 2 |
| BP | GO:0090090 | negative regulation of canonical Wnt signaling pathway | 2/41 | 137/18723 | 0.03619204 | 0.09209077 | 0.05207435 | DDIT3/EGR1 | 2 |
| BP | GO:0042113 | B cell activation | 3/41 | 334/18723 | 0.03642121 | 0.09209077 | 0.05207435 | IRS2/CDKN1A/IL6 | 3 |
| BP | GO:0062012 | regulation of small molecule metabolic process | 3/41 | 334/18723 | 0.03642121 | 0.09209077 | 0.05207435 | IRS2/EGR1/PTGS2 | 3 |
| BP | GO:0001780 | neutrophil homeostasis | 1/41 | 17/18723 | 0.03659723 | 0.09209077 | 0.05207435 | IL6 | 1 |
| BP | GO:0010566 | regulation of ketone biosynthetic process | 1/41 | 17/18723 | 0.03659723 | 0.09209077 | 0.05207435 | EGR1 | 1 |
| BP | GO:0010715 | regulation of extracellular matrix disassembly | 1/41 | 17/18723 | 0.03659723 | 0.09209077 | 0.05207435 | IL6 | 1 |
| BP | GO:0033151 | V(D)J recombination | 1/41 | 17/18723 | 0.03659723 | 0.09209077 | 0.05207435 | HMGB2 | 1 |
| BP | GO:0035729 | cellular response to hepatocyte growth factor stimulus | 1/41 | 17/18723 | 0.03659723 | 0.09209077 | 0.05207435 | RELA | 1 |
| BP | GO:0042448 | progesterone metabolic process | 1/41 | 17/18723 | 0.03659723 | 0.09209077 | 0.05207435 | EGR1 | 1 |
| BP | GO:0042753 | positive regulation of circadian rhythm | 1/41 | 17/18723 | 0.03659723 | 0.09209077 | 0.05207435 | ARNTL | 1 |
| BP | GO:0042772 | DNA damage response, signal transduction resulting in transcription | 1/41 | 17/18723 | 0.03659723 | 0.09209077 | 0.05207435 | CDKN1A | 1 |
| BP | GO:0043117 | positive regulation of vascular permeability | 1/41 | 17/18723 | 0.03659723 | 0.09209077 | 0.05207435 | VEGFA | 1 |
| BP | GO:0044539 | long-chain fatty acid import into cell | 1/41 | 17/18723 | 0.03659723 | 0.09209077 | 0.05207435 | IRS2 | 1 |
| BP | GO:0048339 | paraxial mesoderm development | 1/41 | 17/18723 | 0.03659723 | 0.09209077 | 0.05207435 | DLL3 | 1 |
| BP | GO:0051797 | regulation of hair follicle development | 1/41 | 17/18723 | 0.03659723 | 0.09209077 | 0.05207435 | NGFR | 1 |
| BP | GO:0060644 | mammary gland epithelial cell differentiation | 1/41 | 17/18723 | 0.03659723 | 0.09209077 | 0.05207435 | CEBPB | 1 |
| BP | GO:0060977 | coronary vasculature morphogenesis | 1/41 | 17/18723 | 0.03659723 | 0.09209077 | 0.05207435 | VEGFA | 1 |
| BP | GO:0061450 | trophoblast cell migration | 1/41 | 17/18723 | 0.03659723 | 0.09209077 | 0.05207435 | VEGFA | 1 |
| BP | GO:0070875 | positive regulation of glycogen metabolic process | 1/41 | 17/18723 | 0.03659723 | 0.09209077 | 0.05207435 | IRS2 | 1 |
| BP | GO:0071371 | cellular response to gonadotropin stimulus | 1/41 | 17/18723 | 0.03659723 | 0.09209077 | 0.05207435 | CCNA2 | 1 |
| BP | GO:0090036 | regulation of protein kinase C signaling | 1/41 | 17/18723 | 0.03659723 | 0.09209077 | 0.05207435 | VEGFA | 1 |
| BP | GO:0090713 | immunological memory process | 1/41 | 17/18723 | 0.03659723 | 0.09209077 | 0.05207435 | PCK1 | 1 |
| BP | GO:0140354 | lipid import into cell | 1/41 | 17/18723 | 0.03659723 | 0.09209077 | 0.05207435 | IRS2 | 1 |
| BP | GO:1901550 | regulation of endothelial cell development | 1/41 | 17/18723 | 0.03659723 | 0.09209077 | 0.05207435 | VEGFA | 1 |
| BP | GO:1902894 | negative regulation of pri-miRNA transcription by RNA polymerase II | 1/41 | 17/18723 | 0.03659723 | 0.09209077 | 0.05207435 | RELA | 1 |
| BP | GO:1903140 | regulation of establishment of endothelial barrier | 1/41 | 17/18723 | 0.03659723 | 0.09209077 | 0.05207435 | VEGFA | 1 |
| BP | GO:0016579 | protein deubiquitination | 2/41 | 139/18723 | 0.03715892 | 0.09338685 | 0.05280724 | CDK1/UCHL1 | 2 |
| BP | GO:0001655 | urogenital system development | 3/41 | 338/18723 | 0.03752533 | 0.09418952 | 0.05326112 | VEGFA/EGR1/MYC | 3 |
| BP | GO:0007292 | female gamete generation | 2/41 | 140/18723 | 0.0376461 | 0.09425642 | 0.05329895 | TOP2A/PTGS2 | 2 |
| BP | GO:0050768 | negative regulation of neurogenesis | 2/41 | 140/18723 | 0.0376461 | 0.09425642 | 0.05329895 | IL6/DLL3 | 2 |
| BP | GO:0045765 | regulation of angiogenesis | 3/41 | 342/18723 | 0.03864665 | 0.09466884 | 0.05353216 | VEGFA/IL6/SERPINE1 | 3 |
| BP | GO:0000712 | resolution of meiotic recombination intermediates | 1/41 | 18/18723 | 0.03870883 | 0.09466884 | 0.05353216 | TOP2A | 1 |
| BP | GO:0002076 | osteoblast development | 1/41 | 18/18723 | 0.03870883 | 0.09466884 | 0.05353216 | JUND | 1 |
| BP | GO:0002295 | T-helper cell lineage commitment | 1/41 | 18/18723 | 0.03870883 | 0.09466884 | 0.05353216 | IL6 | 1 |
| BP | GO:0019896 | axonal transport of mitochondrion | 1/41 | 18/18723 | 0.03870883 | 0.09466884 | 0.05353216 | UCHL1 | 1 |
| BP | GO:0030728 | ovulation | 1/41 | 18/18723 | 0.03870883 | 0.09466884 | 0.05353216 | PTGS2 | 1 |
| BP | GO:0031649 | heat generation | 1/41 | 18/18723 | 0.03870883 | 0.09466884 | 0.05353216 | PTGS2 | 1 |
| BP | GO:0035743 | CD4-positive, alpha-beta T cell cytokine production | 1/41 | 18/18723 | 0.03870883 | 0.09466884 | 0.05353216 | IL6 | 1 |
| BP | GO:0036499 | PERK-mediated unfolded protein response | 1/41 | 18/18723 | 0.03870883 | 0.09466884 | 0.05353216 | DDIT3 | 1 |
| BP | GO:0043116 | negative regulation of vascular permeability | 1/41 | 18/18723 | 0.03870883 | 0.09466884 | 0.05353216 | VEGFA | 1 |
| BP | GO:0046851 | negative regulation of bone remodeling | 1/41 | 18/18723 | 0.03870883 | 0.09466884 | 0.05353216 | IL6 | 1 |
| BP | GO:0051782 | negative regulation of cell division | 1/41 | 18/18723 | 0.03870883 | 0.09466884 | 0.05353216 | MYC | 1 |
| BP | GO:0060749 | mammary gland alveolus development | 1/41 | 18/18723 | 0.03870883 | 0.09466884 | 0.05353216 | VEGFA | 1 |
| BP | GO:0061377 | mammary gland lobule development | 1/41 | 18/18723 | 0.03870883 | 0.09466884 | 0.05353216 | VEGFA | 1 |
| BP | GO:0071318 | cellular response to ATP | 1/41 | 18/18723 | 0.03870883 | 0.09466884 | 0.05353216 | PTGS2 | 1 |
| BP | GO:0090190 | positive regulation of branching involved in ureteric bud morphogenesis | 1/41 | 18/18723 | 0.03870883 | 0.09466884 | 0.05353216 | VEGFA | 1 |
| BP | GO:0150078 | positive regulation of neuroinflammatory response | 1/41 | 18/18723 | 0.03870883 | 0.09466884 | 0.05353216 | IL6 | 1 |
| BP | GO:1902001 | fatty acid transmembrane transport | 1/41 | 18/18723 | 0.03870883 | 0.09466884 | 0.05353216 | IRS2 | 1 |
| BP | GO:1902170 | cellular response to reactive nitrogen species | 1/41 | 18/18723 | 0.03870883 | 0.09466884 | 0.05353216 | CCNA2 | 1 |
| BP | GO:0062013 | positive regulation of small molecule metabolic process | 2/41 | 143/18723 | 0.03912243 | 0.0955637 | 0.05403818 | IRS2/PTGS2 | 2 |
| BP | GO:0030336 | negative regulation of cell migration | 3/41 | 344/18723 | 0.03921375 | 0.09567009 | 0.05409834 | IGFBP3/NGFR/SERPINE1 | 3 |
| BP | GO:0043524 | negative regulation of neuron apoptotic process | 2/41 | 145/18723 | 0.04011887 | 0.09755879 | 0.05516633 | CEBPB/NGF | 2 |
| BP | GO:0051961 | negative regulation of nervous system development | 2/41 | 145/18723 | 0.04011887 | 0.09755879 | 0.05516633 | IL6/DLL3 | 2 |
| BP | GO:1901342 | regulation of vasculature development | 3/41 | 348/18723 | 0.04036079 | 0.09755879 | 0.05516633 | VEGFA/IL6/SERPINE1 | 3 |
| BP | GO:0002902 | regulation of B cell apoptotic process | 1/41 | 19/18723 | 0.04081591 | 0.09755879 | 0.05516633 | IRS2 | 1 |
| BP | GO:0006071 | glycerol metabolic process | 1/41 | 19/18723 | 0.04081591 | 0.09755879 | 0.05516633 | PCK1 | 1 |
| BP | GO:0006309 | apoptotic DNA fragmentation | 1/41 | 19/18723 | 0.04081591 | 0.09755879 | 0.05516633 | IL6 | 1 |
| BP | GO:0006977 | DNA damage response, signal transduction by p53 class mediator resulting in cell cycle arrest | 1/41 | 19/18723 | 0.04081591 | 0.09755879 | 0.05516633 | CDKN1A | 1 |
| BP | GO:0031065 | positive regulation of histone deacetylation | 1/41 | 19/18723 | 0.04081591 | 0.09755879 | 0.05516633 | VEGFA | 1 |
| BP | GO:0031290 | retinal ganglion cell axon guidance | 1/41 | 19/18723 | 0.04081591 | 0.09755879 | 0.05516633 | VEGFA | 1 |
| BP | GO:0034501 | protein localization to kinetochore | 1/41 | 19/18723 | 0.04081591 | 0.09755879 | 0.05516633 | CDK1 | 1 |
| BP | GO:0035728 | response to hepatocyte growth factor | 1/41 | 19/18723 | 0.04081591 | 0.09755879 | 0.05516633 | RELA | 1 |
| BP | GO:0044320 | cellular response to leptin stimulus | 1/41 | 19/18723 | 0.04081591 | 0.09755879 | 0.05516633 | CCNA2 | 1 |
| BP | GO:0044827 | modulation by host of viral genome replication | 1/41 | 19/18723 | 0.04081591 | 0.09755879 | 0.05516633 | HSPA8 | 1 |
| BP | GO:0045091 | regulation of single stranded viral RNA replication via double stranded DNA intermediate | 1/41 | 19/18723 | 0.04081591 | 0.09755879 | 0.05516633 | TOP2A | 1 |
| BP | GO:0072074 | kidney mesenchyme development | 1/41 | 19/18723 | 0.04081591 | 0.09755879 | 0.05516633 | MYC | 1 |
| BP | GO:1903083 | protein localization to condensed chromosome | 1/41 | 19/18723 | 0.04081591 | 0.09755879 | 0.05516633 | CDK1 | 1 |
| BP | GO:1903978 | regulation of microglial cell activation | 1/41 | 19/18723 | 0.04081591 | 0.09755879 | 0.05516633 | IL6 | 1 |
| BP | GO:0002449 | lymphocyte mediated immunity | 3/41 | 350/18723 | 0.04094071 | 0.09774046 | 0.05526906 | PRDX1/C1QA/IL6 | 3 |
| BP | GO:2001056 | positive regulation of cysteine-type endopeptidase activity | 2/41 | 148/18723 | 0.04163157 | 0.09927148 | 0.05613481 | MYC/NGFR | 2 |
| BP | GO:0045834 | positive regulation of lipid metabolic process | 2/41 | 149/18723 | 0.04214058 | 0.10024653 | 0.05668616 | IRS2/PTGS2 | 2 |
| BP | GO:0051592 | response to calcium ion | 2/41 | 149/18723 | 0.04214058 | 0.10024653 | 0.05668616 | FOS/JUND | 2 |
| BP | GO:0002460 | adaptive immune response based on somatic recombination of immune receptors built from immunoglobulin superfamily domains | 3/41 | 356/18723 | 0.04270602 | 0.10042724 | 0.05678835 | NFKB2/C1QA/IL6 | 3 |
| BP | GO:0006700 | C21-steroid hormone biosynthetic process | 1/41 | 20/18723 | 0.04291848 | 0.10042724 | 0.05678835 | EGR1 | 1 |
| BP | GO:0010042 | response to manganese ion | 1/41 | 20/18723 | 0.04291848 | 0.10042724 | 0.05678835 | PTGS2 | 1 |
| BP | GO:0015669 | gas transport | 1/41 | 20/18723 | 0.04291848 | 0.10042724 | 0.05678835 | MYC | 1 |
| BP | GO:0032495 | response to muramyl dipeptide | 1/41 | 20/18723 | 0.04291848 | 0.10042724 | 0.05678835 | RELA | 1 |
| BP | GO:0032793 | positive regulation of CREB transcription factor activity | 1/41 | 20/18723 | 0.04291848 | 0.10042724 | 0.05678835 | VEGFA | 1 |
| BP | GO:0032986 | protein-DNA complex disassembly | 1/41 | 20/18723 | 0.04291848 | 0.10042724 | 0.05678835 | MYC | 1 |
| BP | GO:0034104 | negative regulation of tissue remodeling | 1/41 | 20/18723 | 0.04291848 | 0.10042724 | 0.05678835 | IL6 | 1 |
| BP | GO:0039692 | single stranded viral RNA replication via double stranded DNA intermediate | 1/41 | 20/18723 | 0.04291848 | 0.10042724 | 0.05678835 | TOP2A | 1 |
| BP | GO:0048670 | regulation of collateral sprouting | 1/41 | 20/18723 | 0.04291848 | 0.10042724 | 0.05678835 | NGF | 1 |
| BP | GO:0060252 | positive regulation of glial cell proliferation | 1/41 | 20/18723 | 0.04291848 | 0.10042724 | 0.05678835 | IL6 | 1 |
| BP | GO:0071731 | response to nitric oxide | 1/41 | 20/18723 | 0.04291848 | 0.10042724 | 0.05678835 | CCNA2 | 1 |
| BP | GO:1902004 | positive regulation of amyloid-beta formation | 1/41 | 20/18723 | 0.04291848 | 0.10042724 | 0.05678835 | RELA | 1 |
| BP | GO:2000678 | negative regulation of transcription regulatory region DNA binding | 1/41 | 20/18723 | 0.04291848 | 0.10042724 | 0.05678835 | DDIT3 | 1 |
| BP | GO:0043535 | regulation of blood vessel endothelial cell migration | 2/41 | 151/18723 | 0.04316566 | 0.10065288 | 0.05691594 | VEGFA/PTGS2 | 2 |
| BP | GO:0048592 | eye morphogenesis | 2/41 | 151/18723 | 0.04316566 | 0.10065288 | 0.05691594 | VEGFA/EFEMP1 | 2 |
| BP | GO:0048754 | branching morphogenesis of an epithelial tube | 2/41 | 151/18723 | 0.04316566 | 0.10065288 | 0.05691594 | VEGFA/MYC | 2 |
| BP | GO:2000146 | negative regulation of cell motility | 3/41 | 359/18723 | 0.04360299 | 0.10155441 | 0.05742573 | IGFBP3/NGFR/SERPINE1 | 3 |
| BP | GO:0098754 | detoxification | 2/41 | 152/18723 | 0.04368172 | 0.10161961 | 0.0574626 | PRDX1/PTGS2 | 2 |
| BP | GO:0120254 | olefinic compound metabolic process | 2/41 | 153/18723 | 0.04420011 | 0.10270628 | 0.05807707 | EGR1/PTGS2 | 2 |
| BP | GO:0050770 | regulation of axonogenesis | 2/41 | 154/18723 | 0.0447208 | 0.10281433 | 0.05813817 | VEGFA/NGF | 2 |
| BP | GO:0031589 | cell-substrate adhesion | 3/41 | 363/18723 | 0.04481374 | 0.10281433 | 0.05813817 | VEGFA/PLAU/SERPINE1 | 3 |
| BP | GO:0006925 | inflammatory cell apoptotic process | 1/41 | 21/18723 | 0.04501656 | 0.10281433 | 0.05813817 | IL6 | 1 |
| BP | GO:0007289 | spermatid nucleus differentiation | 1/41 | 21/18723 | 0.04501656 | 0.10281433 | 0.05813817 | HMGB2 | 1 |
| BP | GO:0019985 | translesion synthesis | 1/41 | 21/18723 | 0.04501656 | 0.10281433 | 0.05813817 | PCNA | 1 |
| BP | GO:0031998 | regulation of fatty acid beta-oxidation | 1/41 | 21/18723 | 0.04501656 | 0.10281433 | 0.05813817 | IRS2 | 1 |
| BP | GO:0038083 | peptidyl-tyrosine autophosphorylation | 1/41 | 21/18723 | 0.04501656 | 0.10281433 | 0.05813817 | VEGFA | 1 |
| BP | GO:0043373 | CD4-positive, alpha-beta T cell lineage commitment | 1/41 | 21/18723 | 0.04501656 | 0.10281433 | 0.05813817 | IL6 | 1 |
| BP | GO:0046885 | regulation of hormone biosynthetic process | 1/41 | 21/18723 | 0.04501656 | 0.10281433 | 0.05813817 | EGR1 | 1 |
| BP | GO:0071498 | cellular response to fluid shear stress | 1/41 | 21/18723 | 0.04501656 | 0.10281433 | 0.05813817 | PTGS2 | 1 |
| BP | GO:0090026 | positive regulation of monocyte chemotaxis | 1/41 | 21/18723 | 0.04501656 | 0.10281433 | 0.05813817 | SERPINE1 | 1 |
| BP | GO:0090189 | regulation of branching involved in ureteric bud morphogenesis | 1/41 | 21/18723 | 0.04501656 | 0.10281433 | 0.05813817 | VEGFA | 1 |
| BP | GO:0150146 | cell junction disassembly | 1/41 | 21/18723 | 0.04501656 | 0.10281433 | 0.05813817 | C1QA | 1 |
| BP | GO:2000047 | regulation of cell-cell adhesion mediated by cadherin | 1/41 | 21/18723 | 0.04501656 | 0.10281433 | 0.05813817 | VEGFA | 1 |
| BP | GO:2001014 | regulation of skeletal muscle cell differentiation | 1/41 | 21/18723 | 0.04501656 | 0.10281433 | 0.05813817 | ARNTL | 1 |
| BP | GO:0010970 | transport along microtubule | 2/41 | 155/18723 | 0.0452438 | 0.10321565 | 0.0583651 | HSPA8/UCHL1 | 2 |
| BP | GO:0035051 | cardiocyte differentiation | 2/41 | 156/18723 | 0.0457691 | 0.10429522 | 0.05897557 | VEGFA/CDK1 | 2 |
| BP | GO:0001701 | in utero embryonic development | 3/41 | 367/18723 | 0.04604133 | 0.10467739 | 0.05919167 | VEGFA/CEBPB/CEBPA | 3 |
| BP | GO:0051271 | negative regulation of cellular component movement | 3/41 | 367/18723 | 0.04604133 | 0.10467739 | 0.05919167 | IGFBP3/NGFR/SERPINE1 | 3 |
| BP | GO:0070646 | protein modification by small protein removal | 2/41 | 157/18723 | 0.04629667 | 0.1050195 | 0.05938512 | CDK1/UCHL1 | 2 |
| BP | GO:1902600 | proton transmembrane transport | 2/41 | 157/18723 | 0.04629667 | 0.1050195 | 0.05938512 | UCP2/UCP3 | 2 |
| BP | GO:0050900 | leukocyte migration | 3/41 | 369/18723 | 0.04666141 | 0.10507976 | 0.0594192 | VEGFA/IL6/SERPINE1 | 3 |
| BP | GO:0006359 | regulation of transcription by RNA polymerase III | 1/41 | 22/18723 | 0.04711015 | 0.10507976 | 0.0594192 | CEBPA | 1 |
| BP | GO:0019400 | alditol metabolic process | 1/41 | 22/18723 | 0.04711015 | 0.10507976 | 0.0594192 | PCK1 | 1 |
| BP | GO:0022616 | DNA strand elongation | 1/41 | 22/18723 | 0.04711015 | 0.10507976 | 0.0594192 | PCNA | 1 |
| BP | GO:0032700 | negative regulation of interleukin-17 production | 1/41 | 22/18723 | 0.04711015 | 0.10507976 | 0.0594192 | DDIT3 | 1 |
| BP | GO:0032891 | negative regulation of organic acid transport | 1/41 | 22/18723 | 0.04711015 | 0.10507976 | 0.0594192 | IRS2 | 1 |
| BP | GO:0036120 | cellular response to platelet-derived growth factor stimulus | 1/41 | 22/18723 | 0.04711015 | 0.10507976 | 0.0594192 | CCNA2 | 1 |
| BP | GO:0044346 | fibroblast apoptotic process | 1/41 | 22/18723 | 0.04711015 | 0.10507976 | 0.0594192 | MYC | 1 |
| BP | GO:0045663 | positive regulation of myoblast differentiation | 1/41 | 22/18723 | 0.04711015 | 0.10507976 | 0.0594192 | IGFBP3 | 1 |
| BP | GO:0048026 | positive regulation of mRNA splicing, via spliceosome | 1/41 | 22/18723 | 0.04711015 | 0.10507976 | 0.0594192 | HSPA8 | 1 |
| BP | GO:0055093 | response to hyperoxia | 1/41 | 22/18723 | 0.04711015 | 0.10507976 | 0.0594192 | CDKN1A | 1 |
| BP | GO:0060965 | negative regulation of gene silencing by miRNA | 1/41 | 22/18723 | 0.04711015 | 0.10507976 | 0.0594192 | IL6 | 1 |
| BP | GO:0071467 | cellular response to pH | 1/41 | 22/18723 | 0.04711015 | 0.10507976 | 0.0594192 | PCK1 | 1 |
| BP | GO:0140467 | integrated stress response signaling | 1/41 | 22/18723 | 0.04711015 | 0.10507976 | 0.0594192 | DDIT3 | 1 |
| BP | GO:1903589 | positive regulation of blood vessel endothelial cell proliferation involved in sprouting angiogenesis | 1/41 | 22/18723 | 0.04711015 | 0.10507976 | 0.0594192 | VEGFA | 1 |
| BP | GO:0032200 | telomere organization | 2/41 | 159/18723 | 0.04735859 | 0.10539918 | 0.05959982 | PCNA/MYC | 2 |
| BP | GO:0035966 | response to topologically incorrect protein | 2/41 | 159/18723 | 0.04735859 | 0.10539918 | 0.05959982 | DDIT3/HSPA8 | 2 |
| BP | GO:0045862 | positive regulation of proteolysis | 3/41 | 372/18723 | 0.04759939 | 0.10581751 | 0.05983637 | MYC/CEBPA/NGFR | 3 |
| BP | GO:0090316 | positive regulation of intracellular protein transport | 2/41 | 160/18723 | 0.04789293 | 0.10635203 | 0.06013863 | CDK1/PTGS2 | 2 |
| BP | GO:0030098 | lymphocyte differentiation | 3/41 | 374/18723 | 0.04822992 | 0.1064213 | 0.06017779 | PCK1/EGR1/IL6 | 3 |
| BP | GO:0018108 | peptidyl-tyrosine phosphorylation | 3/41 | 375/18723 | 0.04854674 | 0.1064213 | 0.06017779 | VEGFA/EFEMP1/IL6 | 3 |
| BP | GO:0150063 | visual system development | 3/41 | 375/18723 | 0.04854674 | 0.1064213 | 0.06017779 | VEGFA/EFEMP1/C1QA | 3 |
| BP | GO:0034250 | positive regulation of cellular amide metabolic process | 2/41 | 162/18723 | 0.04896827 | 0.1064213 | 0.06017779 | RELA/IL6 | 2 |
| BP | GO:0002052 | positive regulation of neuroblast proliferation | 1/41 | 23/18723 | 0.04919926 | 0.1064213 | 0.06017779 | VEGFA | 1 |
| BP | GO:0002363 | alpha-beta T cell lineage commitment | 1/41 | 23/18723 | 0.04919926 | 0.1064213 | 0.06017779 | IL6 | 1 |
| BP | GO:0003323 | type B pancreatic cell development | 1/41 | 23/18723 | 0.04919926 | 0.1064213 | 0.06017779 | ARNTL | 1 |
| BP | GO:0006622 | protein targeting to lysosome | 1/41 | 23/18723 | 0.04919926 | 0.1064213 | 0.06017779 | HSPA8 | 1 |
| BP | GO:0010888 | negative regulation of lipid storage | 1/41 | 23/18723 | 0.04919926 | 0.1064213 | 0.06017779 | IL6 | 1 |
| BP | GO:0019430 | removal of superoxide radicals | 1/41 | 23/18723 | 0.04919926 | 0.1064213 | 0.06017779 | PRDX1 | 1 |
| BP | GO:0030194 | positive regulation of blood coagulation | 1/41 | 23/18723 | 0.04919926 | 0.1064213 | 0.06017779 | SERPINE1 | 1 |
| BP | GO:0032740 | positive regulation of interleukin-17 production | 1/41 | 23/18723 | 0.04919926 | 0.1064213 | 0.06017779 | IL6 | 1 |
| BP | GO:0035162 | embryonic hemopoiesis | 1/41 | 23/18723 | 0.04919926 | 0.1064213 | 0.06017779 | VEGFA | 1 |
| BP | GO:0036119 | response to platelet-derived growth factor | 1/41 | 23/18723 | 0.04919926 | 0.1064213 | 0.06017779 | CCNA2 | 1 |
| BP | GO:0036303 | lymph vessel morphogenesis | 1/41 | 23/18723 | 0.04919926 | 0.1064213 | 0.06017779 | VEGFA | 1 |
| BP | GO:0042026 | protein refolding | 1/41 | 23/18723 | 0.04919926 | 0.1064213 | 0.06017779 | HSPA8 | 1 |
| BP | GO:0043369 | CD4-positive or CD8-positive, alpha-beta T cell lineage commitment | 1/41 | 23/18723 | 0.04919926 | 0.1064213 | 0.06017779 | IL6 | 1 |
| BP | GO:0045662 | negative regulation of myoblast differentiation | 1/41 | 23/18723 | 0.04919926 | 0.1064213 | 0.06017779 | DDIT3 | 1 |
| BP | GO:0045723 | positive regulation of fatty acid biosynthetic process | 1/41 | 23/18723 | 0.04919926 | 0.1064213 | 0.06017779 | PTGS2 | 1 |
| BP | GO:0045932 | negative regulation of muscle contraction | 1/41 | 23/18723 | 0.04919926 | 0.1064213 | 0.06017779 | PTGS2 | 1 |
| BP | GO:0060575 | intestinal epithelial cell differentiation | 1/41 | 23/18723 | 0.04919926 | 0.1064213 | 0.06017779 | CDKN1A | 1 |
| BP | GO:0071636 | positive regulation of transforming growth factor beta production | 1/41 | 23/18723 | 0.04919926 | 0.1064213 | 0.06017779 | PTGS2 | 1 |
| BP | GO:0090335 | regulation of brown fat cell differentiation | 1/41 | 23/18723 | 0.04919926 | 0.1064213 | 0.06017779 | PTGS2 | 1 |
| BP | GO:1900048 | positive regulation of hemostasis | 1/41 | 23/18723 | 0.04919926 | 0.1064213 | 0.06017779 | SERPINE1 | 1 |
| BP | GO:0018212 | peptidyl-tyrosine modification | 3/41 | 378/18723 | 0.04950345 | 0.10696377 | 0.06048454 | VEGFA/EFEMP1/IL6 | 3 |
| CC | GO:0090575 | RNA polymerase II transcription regulator complex | 8/41 | 168/19550 | 1.89E-09 | 2.32E-07 | 1.75E-07 | DDIT3/JUN/MXI1/CEBPB/FOS/TFDP1/CEBPA/JUND | 8 |
| CC | GO:0005667 | transcription regulator complex | 10/41 | 413/19550 | 9.92E-09 | 6.10E-07 | 4.60E-07 | DDIT3/JUN/MXI1/CEBPB/ARNTL/FOS/TFDP1/RELA/CEBPA/JUND | 10 |
| CC | GO:0000307 | cyclin-dependent protein kinase holoenzyme complex | 4/41 | 42/19550 | 1.76E-06 | 7.21E-05 | 5.43E-05 | CDKN1A/CCNA2/PCNA/CDK1 | 4 |
| CC | GO:1902554 | serine/threonine protein kinase complex | 4/41 | 81/19550 | 2.46E-05 | 0.00075776 | 0.00057067 | CDKN1A/CCNA2/PCNA/CDK1 | 4 |
| CC | GO:1902911 | protein kinase complex | 4/41 | 96/19550 | 4.81E-05 | 0.00118324 | 0.0008911 | CDKN1A/CCNA2/PCNA/CDK1 | 4 |
| CC | GO:0098687 | chromosomal region | 5/41 | 348/19550 | 0.00076884 | 0.01380417 | 0.01039595 | TOP2A/CEBPB/BUB1B/PCNA/CDK1 | 5 |
| CC | GO:0032993 | protein-DNA complex | 4/41 | 199/19550 | 0.0007856 | 0.01380417 | 0.01039595 | DDIT3/FOS/PCNA/JUND | 4 |
| CC | GO:0061695 | transferase complex, transferring phosphorus-containing groups | 4/41 | 239/19550 | 0.0015467 | 0.02280289 | 0.01717291 | CDKN1A/CCNA2/PCNA/CDK1 | 4 |
| CC | GO:0000793 | condensed chromosome | 4/41 | 244/19550 | 0.0016685 | 0.02280289 | 0.01717291 | HMGB2/TOP2A/CEBPB/BUB1B | 4 |
| CC | GO:0000775 | chromosome, centromeric region | 3/41 | 198/19550 | 0.0082209 | 0.09733258 | 0.07330139 | TOP2A/CEBPB/BUB1B | 3 |
| CC | GO:0031093 | platelet alpha granule lumen | 2/41 | 67/19550 | 0.00870454 | 0.09733258 | 0.07330139 | VEGFA/SERPINE1 | 2 |
| CC | GO:0062023 | collagen-containing extracellular matrix | 4/41 | 425/19550 | 0.01182494 | 0.11263418 | 0.08482506 | EFEMP1/ELN/C1QA/SERPINE1 | 4 |
| CC | GO:0000228 | nuclear chromosome | 3/41 | 227/19550 | 0.01190443 | 0.11263418 | 0.08482506 | JUN/TOP2A/PCNA | 3 |
| CC | GO:0031091 | platelet alpha granule | 2/41 | 91/19550 | 0.01561987 | 0.13152985 | 0.09905543 | VEGFA/SERPINE1 | 2 |
| CC | GO:0042470 | melanosome | 2/41 | 109/19550 | 0.02192669 | 0.13152985 | 0.09905543 | PRDX1/HSPA8 | 2 |
| CC | GO:0048770 | pigment granule | 2/41 | 109/19550 | 0.02192669 | 0.13152985 | 0.09905543 | PRDX1/HSPA8 | 2 |
| CC | GO:0000974 | Prp19 complex | 1/41 | 11/19550 | 0.02283445 | 0.13152985 | 0.09905543 | HSPA8 | 1 |
| CC | GO:0005652 | nuclear lamina | 1/41 | 11/19550 | 0.02283445 | 0.13152985 | 0.09905543 | PCNA | 1 |
| CC | GO:0099523 | presynaptic cytosol | 1/41 | 11/19550 | 0.02283445 | 0.13152985 | 0.09905543 | HSPA8 | 1 |
| CC | GO:0000940 | outer kinetochore | 1/41 | 12/19550 | 0.0248849 | 0.13152985 | 0.09905543 | BUB1B | 1 |
| CC | GO:0060198 | clathrin-sculpted vesicle | 1/41 | 12/19550 | 0.0248849 | 0.13152985 | 0.09905543 | HSPA8 | 1 |
| CC | GO:0033391 | chromatoid body | 1/41 | 13/19550 | 0.02693116 | 0.13152985 | 0.09905543 | ARNTL | 1 |
| CC | GO:0045120 | pronucleus | 1/41 | 13/19550 | 0.02693116 | 0.13152985 | 0.09905543 | CCNA2 | 1 |
| CC | GO:0005788 | endoplasmic reticulum lumen | 3/41 | 313/19550 | 0.02768669 | 0.13152985 | 0.09905543 | IGFBP3/PTGS2/IL6 | 3 |
| CC | GO:0034774 | secretory granule lumen | 3/41 | 322/19550 | 0.02976697 | 0.13152985 | 0.09905543 | VEGFA/HSPA8/SERPINE1 | 3 |
| CC | GO:0034399 | nuclear periphery | 2/41 | 130/19550 | 0.03038823 | 0.13152985 | 0.09905543 | CEBPB/PCNA | 2 |
| CC | GO:0060205 | cytoplasmic vesicle lumen | 3/41 | 325/19550 | 0.03047844 | 0.13152985 | 0.09905543 | VEGFA/HSPA8/SERPINE1 | 3 |
| CC | GO:0031983 | vesicle lumen | 3/41 | 327/19550 | 0.03095776 | 0.13152985 | 0.09905543 | VEGFA/HSPA8/SERPINE1 | 3 |
| CC | GO:0030894 | replisome | 1/41 | 15/19550 | 0.0310111 | 0.13152985 | 0.09905543 | PCNA | 1 |
| CC | GO:0099524 | postsynaptic cytosol | 1/41 | 17/19550 | 0.03507436 | 0.13989977 | 0.10535884 | HSPA8 | 1 |
| CC | GO:0044306 | neuron projection terminus | 2/41 | 141/19550 | 0.03525929 | 0.13989977 | 0.10535884 | HSPA8/UCHL1 | 2 |
| CC | GO:0000779 | condensed chromosome, centromeric region | 2/41 | 144/19550 | 0.03663728 | 0.14082455 | 0.10605529 | CEBPB/BUB1B | 2 |
| CC | GO:0005680 | anaphase-promoting complex | 1/41 | 21/19550 | 0.04315107 | 0.15610534 | 0.11756328 | BUB1B | 1 |
| CC | GO:0099522 | cytosolic region | 1/41 | 21/19550 | 0.04315107 | 0.15610534 | 0.11756328 | HSPA8 | 1 |
| CC | GO:0000781 | chromosome, telomeric region | 2/41 | 162/19550 | 0.0453284 | 0.15929694 | 0.11996688 | PCNA/CDK1 | 2 |
| MF | GO:0001228 | DNA-binding transcription activator activity, RNA polymerase II-specific | 11/41 | 450/18368 | 2.75E-09 | 3.07E-07 | 1.98E-07 | DDIT3/JUN/CEBPB/FOS/EGR1/TFDP1/NFKB2/RELA/MYC/CEBPA/JUND | 11 |
| MF | GO:0001216 | DNA-binding transcription activator activity | 11/41 | 456/18368 | 3.16E-09 | 3.07E-07 | 1.98E-07 | DDIT3/JUN/CEBPB/FOS/EGR1/TFDP1/NFKB2/RELA/MYC/CEBPA/JUND | 11 |
| MF | GO:0140297 | DNA-binding transcription factor binding | 10/41 | 394/18368 | 1.14E-08 | 7.37E-07 | 4.76E-07 | DDIT3/JUN/CEBPB/ARNTL/FOS/PCNA/RELA/MYC/CEBPA/JUND | 10 |
| MF | GO:0061629 | RNA polymerase II-specific DNA-binding transcription factor binding | 8/41 | 299/18368 | 2.69E-07 | 1.30E-05 | 8.43E-06 | JUN/CEBPB/ARNTL/FOS/PCNA/RELA/CEBPA/JUND | 8 |
| MF | GO:0001046 | core promoter sequence-specific DNA binding | 4/41 | 42/18368 | 2.25E-06 | 8.72E-05 | 5.63E-05 | CEBPB/FOS/RELA/MYC | 4 |
| MF | GO:0044389 | ubiquitin-like protein ligase binding | 7/41 | 316/18368 | 5.68E-06 | 0.00018364 | 0.00011857 | JUN/CDKN1A/CEBPB/RELA/HSPA8/UCHL1/NGFR | 7 |
| MF | GO:0000979 | RNA polymerase II core promoter sequence-specific DNA binding | 3/41 | 20/18368 | 1.15E-05 | 0.00031765 | 0.0002051 | CEBPB/FOS/RELA | 3 |
| MF | GO:0035035 | histone acetyltransferase binding | 3/41 | 25/18368 | 2.29E-05 | 0.00055642 | 0.00035927 | CEBPB/EGR1/PCNA | 3 |
| MF | GO:0031625 | ubiquitin protein ligase binding | 6/41 | 297/18368 | 4.75E-05 | 0.00102297 | 0.00066052 | JUN/CDKN1A/RELA/HSPA8/UCHL1/NGFR | 6 |
| MF | GO:0016538 | cyclin-dependent protein serine/threonine kinase regulator activity | 3/41 | 50/18368 | 0.0001881 | 0.00364918 | 0.00235622 | CDKN1A/CCNA2/CDKN2B | 3 |
| MF | GO:0004861 | cyclin-dependent protein serine/threonine kinase inhibitor activity | 2/41 | 12/18368 | 0.00031633 | 0.00557889 | 0.00360222 | CDKN1A/CDKN2B | 2 |
| MF | GO:0008083 | growth factor activity | 4/41 | 162/18368 | 0.00045788 | 0.00740233 | 0.00477958 | VEGFA/EFEMP1/NGF/IL6 | 4 |
| MF | GO:0001227 | DNA-binding transcription repressor activity, RNA polymerase II-specific | 5/41 | 310/18368 | 0.00060377 | 0.00873909 | 0.00564271 | JUN/MXI1/CEBPB/RELA/MYC | 5 |
| MF | GO:0001217 | DNA-binding transcription repressor activity | 5/41 | 313/18368 | 0.00063066 | 0.00873909 | 0.00564271 | JUN/MXI1/CEBPB/RELA/MYC | 5 |
| MF | GO:0008301 | DNA binding, bending | 2/41 | 18/18368 | 0.00072711 | 0.00940398 | 0.00607202 | HMGB2/TOP2A | 2 |
| MF | GO:0001540 | amyloid-beta binding | 3/41 | 84/18368 | 0.00086759 | 0.01051953 | 0.00679232 | CACNA1A/C1QA/NGFR | 3 |
| MF | GO:0070412 | R-SMAD binding | 2/41 | 23/18368 | 0.00119388 | 0.01362431 | 0.00879703 | JUN/FOS | 2 |
| MF | GO:0001968 | fibronectin binding | 2/41 | 28/18368 | 0.0017712 | 0.01908955 | 0.01232586 | VEGFA/IGFBP3 | 2 |
| MF | GO:0030332 | cyclin binding | 2/41 | 32/18368 | 0.00231103 | 0.02359679 | 0.01523612 | CDKN1A/CDK1 | 2 |
| MF | GO:0030291 | protein serine/threonine kinase inhibitor activity | 2/41 | 34/18368 | 0.00260652 | 0.02528322 | 0.01632503 | CDKN1A/CDKN2B | 2 |
| MF | GO:0042826 | histone deacetylase binding | 3/41 | 127/18368 | 0.00284061 | 0.02624178 | 0.01694396 | TOP2A/CEBPB/RELA | 3 |
| MF | GO:0042056 | chemoattractant activity | 2/41 | 37/18368 | 0.0030813 | 0.0271715 | 0.01754427 | VEGFA/HMGB2 | 2 |
| MF | GO:0070851 | growth factor receptor binding | 3/41 | 141/18368 | 0.0038135 | 0.03216607 | 0.02076919 | VEGFA/EFEMP1/IL6 | 3 |
| MF | GO:0016922 | nuclear receptor binding | 3/41 | 144/18368 | 0.00404523 | 0.03269897 | 0.02111328 | CEBPB/PCNA/JUND | 3 |
| MF | GO:0048018 | receptor ligand activity | 5/41 | 487/18368 | 0.00437921 | 0.03398265 | 0.02194214 | VEGFA/HMGB2/EFEMP1/NGF/IL6 | 5 |
| MF | GO:0030546 | signaling receptor activator activity | 5/41 | 495/18368 | 0.00469092 | 0.03500145 | 0.02259996 | VEGFA/HMGB2/EFEMP1/NGF/IL6 | 5 |
| MF | GO:0070888 | E-box binding | 2/41 | 48/18368 | 0.00513853 | 0.03692131 | 0.02383959 | ARNTL/MYC | 2 |
| MF | GO:0042277 | peptide binding | 4/41 | 321/18368 | 0.0055727 | 0.03861087 | 0.02493051 | CACNA1A/RELA/C1QA/NGFR | 4 |
| MF | GO:0004601 | peroxidase activity | 2/41 | 51/18368 | 0.00578371 | 0.03869102 | 0.02498226 | PRDX1/PTGS2 | 2 |
| MF | GO:0016684 | oxidoreductase activity, acting on peroxide as acceptor | 2/41 | 55/18368 | 0.00669852 | 0.0433171 | 0.02796926 | PRDX1/PTGS2 | 2 |
| MF | GO:0050840 | extracellular matrix binding | 2/41 | 56/18368 | 0.00693685 | 0.04341127 | 0.02803007 | VEGFA/ELN | 2 |
| MF | GO:0003684 | damaged DNA binding | 2/41 | 68/18368 | 0.01008959 | 0.06086121 | 0.03929726 | HMGB2/PCNA | 2 |
| MF | GO:0019887 | protein kinase regulator activity | 3/41 | 204/18368 | 0.01055389 | 0.06086121 | 0.03929726 | CDKN1A/CCNA2/CDKN2B | 3 |
| MF | GO:0004860 | protein kinase inhibitor activity | 2/41 | 70/18368 | 0.0106664 | 0.06086121 | 0.03929726 | CDKN1A/CDKN2B | 2 |
| MF | GO:0019210 | kinase inhibitor activity | 2/41 | 74/18368 | 0.01186282 | 0.064064 | 0.04136526 | CDKN1A/CDKN2B | 2 |
| MF | GO:0033218 | amide binding | 4/41 | 400/18368 | 0.01188817 | 0.064064 | 0.04136526 | CACNA1A/RELA/C1QA/NGFR | 4 |
| MF | GO:0046332 | SMAD binding | 2/41 | 79/18368 | 0.01343726 | 0.07045482 | 0.04549172 | JUN/FOS | 2 |
| MF | GO:0019207 | kinase regulator activity | 3/41 | 233/18368 | 0.01506984 | 0.07693548 | 0.04967619 | CDKN1A/CCNA2/CDKN2B | 3 |
| MF | GO:0016209 | antioxidant activity | 2/41 | 86/18368 | 0.01578512 | 0.07852083 | 0.05069983 | PRDX1/PTGS2 | 2 |
| MF | GO:0043130 | ubiquitin binding | 2/41 | 92/18368 | 0.01792721 | 0.08694699 | 0.05614049 | TOP2A/UCHL1 | 2 |
| MF | GO:0008331 | high voltage-gated calcium channel activity | 1/41 | 10/18368 | 0.02210391 | 0.10102681 | 0.06523163 | CACNA1A | 1 |
| MF | GO:0050786 | RAGE receptor binding | 1/41 | 10/18368 | 0.02210391 | 0.10102681 | 0.06523163 | HMGB2 | 1 |
| MF | GO:0005126 | cytokine receptor binding | 3/41 | 271/18368 | 0.02241478 | 0.10102681 | 0.06523163 | VEGFA/NGF/IL6 | 3 |
| MF | GO:0032404 | mismatch repair complex binding | 1/41 | 11/18368 | 0.0242879 | 0.10102681 | 0.06523163 | PCNA | 1 |
| MF | GO:0043522 | leucine zipper domain binding | 1/41 | 11/18368 | 0.0242879 | 0.10102681 | 0.06523163 | DDIT3 | 1 |
| MF | GO:0097493 | structural molecule activity conferring elasticity | 1/41 | 11/18368 | 0.0242879 | 0.10102681 | 0.06523163 | ELN | 1 |
| MF | GO:0032182 | ubiquitin-like protein binding | 2/41 | 111/18368 | 0.0254608 | 0.10102681 | 0.06523163 | TOP2A/UCHL1 | 2 |
| MF | GO:0005165 | neurotrophin receptor binding | 1/41 | 12/18368 | 0.02646714 | 0.10102681 | 0.06523163 | NGF | 1 |
| MF | GO:0008353 | RNA polymerase II CTD heptapeptide repeat kinase activity | 1/41 | 12/18368 | 0.02646714 | 0.10102681 | 0.06523163 | CDK1 | 1 |
| MF | GO:0097677 | STAT family protein binding | 1/41 | 12/18368 | 0.02646714 | 0.10102681 | 0.06523163 | CEBPA | 1 |
| MF | GO:0005035 | death receptor activity | 1/41 | 13/18368 | 0.02864162 | 0.10102681 | 0.06523163 | NGFR | 1 |
| MF | GO:0019104 | DNA N-glycosylase activity | 1/41 | 13/18368 | 0.02864162 | 0.10102681 | 0.06523163 | PCNA | 1 |
| MF | GO:0031994 | insulin-like growth factor I binding | 1/41 | 13/18368 | 0.02864162 | 0.10102681 | 0.06523163 | IGFBP3 | 1 |
| MF | GO:0042301 | phosphate ion binding | 1/41 | 13/18368 | 0.02864162 | 0.10102681 | 0.06523163 | RELA | 1 |
| MF | GO:0072542 | protein phosphatase activator activity | 1/41 | 13/18368 | 0.02864162 | 0.10102681 | 0.06523163 | IGFBP3 | 1 |
| MF | GO:0005172 | vascular endothelial growth factor receptor binding | 1/41 | 14/18368 | 0.03081137 | 0.10131195 | 0.06541575 | VEGFA | 1 |
| MF | GO:0030983 | mismatched DNA binding | 1/41 | 14/18368 | 0.03081137 | 0.10131195 | 0.06541575 | PCNA | 1 |
| MF | GO:0035259 | glucocorticoid receptor binding | 1/41 | 14/18368 | 0.03081137 | 0.10131195 | 0.06541575 | CEBPB | 1 |
| MF | GO:0071837 | HMG box domain binding | 1/41 | 14/18368 | 0.03081137 | 0.10131195 | 0.06541575 | JUN | 1 |
| MF | GO:0005161 | platelet-derived growth factor receptor binding | 1/41 | 15/18368 | 0.03297638 | 0.10650807 | 0.06877081 | VEGFA | 1 |
| MF | GO:0031072 | heat shock protein binding | 2/41 | 129/18368 | 0.03357882 | 0.10650807 | 0.06877081 | ARNTL/HSPA8 | 2 |
| MF | GO:0008191 | metalloendopeptidase inhibitor activity | 1/41 | 16/18368 | 0.03513668 | 0.10650807 | 0.06877081 | NGF | 1 |
| MF | GO:0035173 | histone kinase activity | 1/41 | 16/18368 | 0.03513668 | 0.10650807 | 0.06877081 | CDK1 | 1 |
| MF | GO:0035497 | cAMP response element binding | 1/41 | 16/18368 | 0.03513668 | 0.10650807 | 0.06877081 | JUN | 1 |
| MF | GO:0046982 | protein heterodimerization activity | 3/41 | 328/18368 | 0.03651077 | 0.10798061 | 0.06972161 | DDIT3/TOP2A/CEBPB | 3 |
| MF | GO:0000400 | four-way junction DNA binding | 1/41 | 17/18368 | 0.03729227 | 0.10798061 | 0.06972161 | HMGB2 | 1 |
| MF | GO:0030275 | LRR domain binding | 1/41 | 17/18368 | 0.03729227 | 0.10798061 | 0.06972161 | DDIT3 | 1 |
| MF | GO:0140597 | protein carrier activity | 1/41 | 18/18368 | 0.03944317 | 0.11099956 | 0.07167091 | HSPA8 | 1 |
| MF | GO:0019838 | growth factor binding | 2/41 | 141/18368 | 0.03947923 | 0.11099956 | 0.07167091 | IGFBP3/NGFR | 2 |
| MF | GO:0019211 | phosphatase activator activity | 1/41 | 20/18368 | 0.04373089 | 0.11949005 | 0.0771531 | IGFBP3 | 1 |
| MF | GO:0031690 | adrenergic receptor binding | 1/41 | 20/18368 | 0.04373089 | 0.11949005 | 0.0771531 | UCHL1 | 1 |
| MF | GO:0005123 | death receptor binding | 1/41 | 21/18368 | 0.04586775 | 0.12065916 | 0.07790798 | NGF | 1 |
| MF | GO:0140416 | transcription regulator inhibitor activity | 1/41 | 21/18368 | 0.04586775 | 0.12065916 | 0.07790798 | DDIT3 | 1 |
| MF | GO:0106310 | protein serine kinase activity | 3/41 | 360/18368 | 0.04602463 | 0.12065916 | 0.07790798 | BUB1B/PCK1/CDK1 | 3 |
| MF | GO:0070182 | DNA polymerase binding | 1/41 | 22/18368 | 0.04799995 | 0.12415986 | 0.08016833 | PCNA | 1 |
