# Supplementary material for: Identification of aging-related genes in diagnosing osteoarthritis via integrating bioinformatics analysis and machine learning
Source: Aging (Albany NY). 2024 Jan 3;16(1):153–68. doi: 10.18632/aging.205357 (PMC10817387; doi:10.18632/aging.205357)
Supplement: Supplementary Table 2 [file aging-16-205357-s002.docx]

| **Supplementary Table 2. Results of KEGG function enrichment analysis.** | | | | | | | | |
| --- | --- | --- | --- | --- | --- | --- | --- | --- |
| ID | Description | GeneRatio | BgRatio | pvalue | p.adjust | qvalue | geneID | Count |
| hsa05202 | Transcriptional misregulation in cancer | 11/36 | 193/8163 | 3.48E-10 | 6.20E-08 | 3.04E-08 | DDIT3/CDKN1A/IGFBP3/CEBPB/CCNA2/RELA/MYC/CEBPA/PLAU/IL6/NGFR | 11 |
| hsa05166 | Human T-cell leukemia virus 1 infection | 11/36 | 222/8163 | 1.55E-09 | 1.30E-07 | 6.40E-08 | JUN/CDKN1A/CCNA2/FOS/BUB1B/EGR1/NFKB2/RELA/CDKN2B/MYC/IL6 | 11 |
| hsa04010 | MAPK signaling pathway | 12/36 | 294/8163 | 2.20E-09 | 1.30E-07 | 6.40E-08 | DDIT3/JUN/VEGFA/FOS/CACNA1A/NGF/NFKB2/RELA/HSPA8/MYC/JUND/NGFR | 12 |
| hsa04218 | Cellular senescence | 9/36 | 156/8163 | 1.63E-08 | 7.27E-07 | 3.57E-07 | CDKN1A/IGFBP3/CCNA2/CDK1/RELA/CDKN2B/MYC/IL6/SERPINE1 | 9 |
| hsa04110 | Cell cycle | 8/36 | 126/8163 | 5.43E-08 | 1.93E-06 | 9.49E-07 | CDKN1A/CCNA2/BUB1B/PCNA/CDK1/TFDP1/CDKN2B/MYC | 8 |
| hsa04657 | IL-17 signaling pathway | 7/36 | 94/8163 | 1.36E-07 | 4.04E-06 | 1.98E-06 | JUN/CEBPB/FOS/RELA/PTGS2/JUND/IL6 | 7 |
| hsa05161 | Hepatitis B | 8/36 | 162/8163 | 3.82E-07 | 9.71E-06 | 4.77E-06 | JUN/CDKN1A/CCNA2/FOS/PCNA/RELA/MYC/IL6 | 8 |
| hsa05167 | Kaposi sarcoma-associated herpesvirus infection | 8/36 | 194/8163 | 1.51E-06 | 3.35E-05 | 1.65E-05 | JUN/CDKN1A/VEGFA/FOS/RELA/MYC/PTGS2/IL6 | 8 |
| hsa04932 | Non-alcoholic fatty liver disease | 7/36 | 155/8163 | 4.09E-06 | 7.48E-05 | 3.67E-05 | DDIT3/IRS2/JUN/FOS/RELA/CEBPA/IL6 | 7 |
| hsa04933 | AGE-RAGE signaling pathway in diabetic complications | 6/36 | 100/8163 | 4.20E-06 | 7.48E-05 | 3.67E-05 | JUN/VEGFA/EGR1/RELA/IL6/SERPINE1 | 6 |
| hsa05142 | Chagas disease | 6/36 | 102/8163 | 4.72E-06 | 7.64E-05 | 3.75E-05 | JUN/FOS/RELA/C1QA/IL6/SERPINE1 | 6 |
| hsa04668 | TNF signaling pathway | 6/36 | 112/8163 | 8.12E-06 | 0.0001205 | 5.91E-05 | JUN/CEBPB/FOS/RELA/PTGS2/IL6 | 6 |
| hsa05133 | Pertussis | 5/36 | 76/8163 | 1.84E-05 | 0.00025236 | 0.00012387 | JUN/FOS/RELA/C1QA/IL6 | 5 |
| hsa05169 | Epstein-Barr virus infection | 7/36 | 202/8163 | 2.32E-05 | 0.0002939 | 0.00014425 | JUN/CDKN1A/CCNA2/NFKB2/RELA/MYC/IL6 | 7 |
| hsa05203 | Viral carcinogenesis | 7/36 | 204/8163 | 2.48E-05 | 0.0002939 | 0.00014425 | JUN/CDKN1A/CCNA2/CDK1/NFKB2/RELA/CDKN2B | 7 |
| hsa05207 | Chemical carcinogenesis - receptor activation | 7/36 | 212/8163 | 3.17E-05 | 0.00035308 | 0.00017331 | JUN/VEGFA/FOS/CACNA1A/RELA/MYC/DLL3 | 7 |
| hsa05224 | Breast cancer | 6/36 | 147/8163 | 3.84E-05 | 0.00040213 | 0.00019738 | JUN/CDKN1A/FOS/NFKB2/MYC/DLL3 | 6 |
| hsa05222 | Small cell lung cancer | 5/36 | 92/8163 | 4.66E-05 | 0.00046112 | 0.00022633 | CDKN1A/RELA/CDKN2B/MYC/PTGS2 | 5 |
| hsa04625 | C-type lectin receptor signaling pathway | 5/36 | 104/8163 | 8.39E-05 | 0.00078638 | 0.00038598 | JUN/NFKB2/RELA/PTGS2/IL6 | 5 |
| hsa04066 | HIF-1 signaling pathway | 5/36 | 109/8163 | 0.00010496 | 0.00090327 | 0.00044336 | CDKN1A/VEGFA/RELA/IL6/SERPINE1 | 5 |
| hsa05134 | Legionellosis | 4/36 | 57/8163 | 0.00010657 | 0.00090327 | 0.00044336 | NFKB2/RELA/HSPA8/IL6 | 4 |
| hsa04151 | PI3K-Akt signaling pathway | 8/36 | 354/8163 | 0.00012027 | 0.00097309 | 0.00047762 | CDKN1A/VEGFA/PCK1/NGF/RELA/MYC/IL6/NGFR | 8 |
| hsa05221 | Acute myeloid leukemia | 4/36 | 67/8163 | 0.0002004 | 0.00155088 | 0.00076123 | CCNA2/RELA/MYC/CEBPA | 4 |
| hsa04380 | Osteoclast differentiation | 5/36 | 128/8163 | 0.00022377 | 0.00165961 | 0.00081459 | JUN/FOS/NFKB2/RELA/JUND | 5 |
| hsa04068 | FoxO signaling pathway | 5/36 | 131/8163 | 0.00024932 | 0.00177518 | 0.00087132 | IRS2/CDKN1A/PCK1/CDKN2B/IL6 | 5 |
| hsa04115 | p53 signaling pathway | 4/36 | 73/8163 | 0.00027927 | 0.00191189 | 0.00093842 | CDKN1A/IGFBP3/CDK1/SERPINE1 | 4 |
| hsa04210 | Apoptosis | 5/36 | 136/8163 | 0.00029679 | 0.00195663 | 0.00096038 | DDIT3/JUN/FOS/NGF/RELA | 5 |
| hsa05417 | Lipid and atherosclerosis | 6/36 | 215/8163 | 0.00031304 | 0.00199007 | 0.00097679 | DDIT3/JUN/FOS/RELA/HSPA8/IL6 | 6 |
| hsa05162 | Measles | 5/36 | 139/8163 | 0.0003284 | 0.00201568 | 0.00098936 | JUN/FOS/RELA/HSPA8/IL6 | 5 |
| hsa05140 | Leishmaniasis | 4/36 | 77/8163 | 0.00034289 | 0.00203448 | 0.00099859 | JUN/FOS/RELA/PTGS2 | 4 |
| hsa05163 | Human cytomegalovirus infection | 6/36 | 225/8163 | 0.0003996 | 0.00229447 | 0.0011262 | CDKN1A/VEGFA/RELA/MYC/PTGS2/IL6 | 6 |
| hsa05210 | Colorectal cancer | 4/36 | 86/8163 | 0.0005231 | 0.00290973 | 0.00142819 | JUN/CDKN1A/FOS/MYC | 4 |
| hsa04658 | Th1 and Th2 cell differentiation | 4/36 | 92/8163 | 0.00067547 | 0.00364343 | 0.00178832 | JUN/FOS/RELA/DLL3 | 4 |
| hsa05323 | Rheumatoid arthritis | 4/36 | 93/8163 | 0.00070362 | 0.00368365 | 0.00180806 | JUN/VEGFA/FOS/IL6 | 4 |
| hsa05219 | Bladder cancer | 3/36 | 41/8163 | 0.00074849 | 0.0038066 | 0.00186841 | CDKN1A/VEGFA/MYC | 3 |
| hsa01522 | Endocrine resistance | 4/36 | 98/8163 | 0.00085698 | 0.00423731 | 0.00207982 | JUN/CDKN1A/FOS/DLL3 | 4 |
| hsa04064 | NF-kappa B signaling pathway | 4/36 | 104/8163 | 0.00107056 | 0.00501474 | 0.0024614 | NFKB2/RELA/PTGS2/PLAU | 4 |
| hsa04620 | Toll-like receptor signaling pathway | 4/36 | 104/8163 | 0.00107056 | 0.00501474 | 0.0024614 | JUN/FOS/RELA/IL6 | 4 |
| hsa04928 | Parathyroid hormone synthesis, secretion and action | 4/36 | 106/8163 | 0.00114939 | 0.00524591 | 0.00257487 | CDKN1A/FOS/EGR1/JUND | 4 |
| hsa04659 | Th17 cell differentiation | 4/36 | 108/8163 | 0.00123221 | 0.00534958 | 0.00262575 | JUN/FOS/RELA/IL6 | 4 |
| hsa04931 | Insulin resistance | 4/36 | 108/8163 | 0.00123221 | 0.00534958 | 0.00262575 | IRS2/PCK1/RELA/IL6 | 4 |
| hsa04722 | Neurotrophin signaling pathway | 4/36 | 119/8163 | 0.00176401 | 0.00747604 | 0.00366949 | JUN/NGF/RELA/NGFR | 4 |
| hsa05206 | MicroRNAs in cancer | 6/36 | 310/8163 | 0.0021241 | 0.00879281 | 0.00431581 | IRS2/CDKN1A/VEGFA/MYC/PTGS2/PLAU | 6 |
| hsa04926 | Relaxin signaling pathway | 4/36 | 129/8163 | 0.00237036 | 0.0095892 | 0.0047067 | JUN/VEGFA/FOS/RELA | 4 |
| hsa05321 | Inflammatory bowel disease | 3/36 | 65/8163 | 0.00285247 | 0.01128311 | 0.00553813 | JUN/RELA/IL6 | 3 |
| hsa05135 | Yersinia infection | 4/36 | 137/8163 | 0.002949 | 0.01141133 | 0.00560107 | JUN/FOS/RELA/IL6 | 4 |
| hsa05418 | Fluid shear stress and atherosclerosis | 4/36 | 139/8163 | 0.00310752 | 0.01176891 | 0.00577658 | JUN/VEGFA/FOS/RELA | 4 |
| hsa05171 | Coronavirus disease - COVID-19 | 5/36 | 232/8163 | 0.00326043 | 0.01203458 | 0.00590698 | JUN/FOS/RELA/C1QA/IL6 | 5 |
| hsa04920 | Adipocytokine signaling pathway | 3/36 | 69/8163 | 0.0033805 | 0.01203458 | 0.00590698 | IRS2/PCK1/RELA | 3 |
| hsa05211 | Renal cell carcinoma | 3/36 | 69/8163 | 0.0033805 | 0.01203458 | 0.00590698 | JUN/CDKN1A/VEGFA | 3 |
| hsa05132 | Salmonella infection | 5/36 | 249/8163 | 0.00441257 | 0.01478978 | 0.00725932 | JUN/FOS/RELA/MYC/IL6 | 5 |
| hsa05212 | Pancreatic cancer | 3/36 | 76/8163 | 0.00444111 | 0.01478978 | 0.00725932 | CDKN1A/VEGFA/RELA | 3 |
| hsa05220 | Chronic myeloid leukemia | 3/36 | 76/8163 | 0.00444111 | 0.01478978 | 0.00725932 | CDKN1A/RELA/MYC | 3 |
| hsa04921 | Oxytocin signaling pathway | 4/36 | 154/8163 | 0.00448679 | 0.01478978 | 0.00725932 | JUN/CDKN1A/FOS/PTGS2 | 4 |
| hsa04662 | B cell receptor signaling pathway | 3/36 | 82/8163 | 0.00549447 | 0.01778211 | 0.00872806 | JUN/FOS/RELA | 3 |
| hsa04012 | ErbB signaling pathway | 3/36 | 85/8163 | 0.00607284 | 0.0189643 | 0.00930832 | JUN/CDKN1A/MYC | 3 |
| hsa04610 | Complement and coagulation cascades | 3/36 | 85/8163 | 0.00607284 | 0.0189643 | 0.00930832 | C1QA/PLAU/SERPINE1 | 3 |
| hsa05020 | Prion disease | 5/36 | 273/8163 | 0.00650104 | 0.01995147 | 0.00979286 | DDIT3/EGR1/HSPA8/C1QA/IL6 | 5 |
| hsa05235 | PD-L1 expression and PD-1 checkpoint pathway in cancer | 3/36 | 89/8163 | 0.00689891 | 0.02081367 | 0.01021605 | JUN/FOS/RELA | 3 |
| hsa01523 | Antifolate resistance | 2/36 | 30/8163 | 0.00761221 | 0.02258288 | 0.01108444 | RELA/IL6 | 2 |
| hsa04350 | TGF-beta signaling pathway | 3/36 | 94/8163 | 0.00802154 | 0.02340711 | 0.011489 | TFDP1/CDKN2B/MYC | 3 |
| hsa05215 | Prostate cancer | 3/36 | 97/8163 | 0.00874399 | 0.02510371 | 0.01232175 | CDKN1A/RELA/PLAU | 3 |
| hsa05146 | Amoebiasis | 3/36 | 102/8163 | 0.01003094 | 0.02834138 | 0.01391091 | PRDX1/RELA/IL6 | 3 |
| hsa04660 | T cell receptor signaling pathway | 3/36 | 104/8163 | 0.01057506 | 0.02902001 | 0.01424401 | JUN/FOS/RELA | 3 |
| hsa05130 | Pathogenic Escherichia coli infection | 4/36 | 197/8163 | 0.0105972 | 0.02902001 | 0.01424401 | JUN/FOS/RELA/IL6 | 4 |
| hsa05216 | Thyroid cancer | 2/36 | 37/8163 | 0.01143165 | 0.03083081 | 0.0151328 | CDKN1A/MYC | 2 |
| hsa05205 | Proteoglycans in cancer | 4/36 | 205/8163 | 0.01213496 | 0.03223915 | 0.01582407 | CDKN1A/VEGFA/MYC/PLAU | 4 |
| hsa05170 | Human immunodeficiency virus 1 infection | 4/36 | 212/8163 | 0.01359324 | 0.0355823 | 0.017465 | JUN/FOS/CDK1/RELA | 4 |
| hsa05165 | Human papillomavirus infection | 5/36 | 331/8163 | 0.01426381 | 0.03679649 | 0.01806096 | CDKN1A/VEGFA/CCNA2/RELA/PTGS2 | 5 |
| hsa04935 | Growth hormone synthesis, secretion and action | 3/36 | 120/8163 | 0.01554469 | 0.03952792 | 0.01940164 | IRS2/IGFBP3/FOS | 3 |
| hsa04152 | AMPK signaling pathway | 3/36 | 121/8163 | 0.01589221 | 0.0398134 | 0.01954176 | IRS2/CCNA2/PCK1 | 3 |
| hsa05208 | Chemical carcinogenesis - reactive oxygen species | 4/36 | 223/8163 | 0.0161043 | 0.0398134 | 0.01954176 | JUN/VEGFA/FOS/RELA | 4 |
| hsa04930 | Type II diabetes mellitus | 2/36 | 46/8163 | 0.01733067 | 0.04225835 | 0.02074183 | IRS2/CACNA1A | 2 |
| hsa04014 | Ras signaling pathway | 4/36 | 235/8163 | 0.0191596 | 0.04608661 | 0.02262087 | VEGFA/NGF/RELA/NGFR | 4 |
| hsa05030 | Cocaine addiction | 2/36 | 49/8163 | 0.01952997 | 0.04635114 | 0.02275071 | JUN/RELA | 2 |
| hsa04728 | Dopaminergic synapse | 3/36 | 132/8163 | 0.02000595 | 0.04685605 | 0.02299853 | ARNTL/FOS/CACNA1A | 3 |
| hsa04915 | Estrogen signaling pathway | 3/36 | 138/8163 | 0.02247596 | 0.05195741 | 0.02550245 | JUN/FOS/HSPA8 | 3 |
| hsa04936 | Alcoholic liver disease | 3/36 | 142/8163 | 0.02421167 | 0.05525226 | 0.02711968 | RELA/C1QA/IL6 | 3 |
| hsa05010 | Alzheimer disease | 5/36 | 384/8163 | 0.025448 | 0.05733853 | 0.02814369 | DDIT3/IRS2/RELA/PTGS2/IL6 | 5 |
| hsa04923 | Regulation of lipolysis in adipocytes | 2/36 | 57/8163 | 0.02592945 | 0.05769302 | 0.02831768 | IRS2/PTGS2 | 2 |
| hsa05213 | Endometrial cancer | 2/36 | 58/8163 | 0.026782 | 0.05885426 | 0.02888766 | CDKN1A/MYC | 2 |
| hsa05226 | Gastric cancer | 3/36 | 149/8163 | 0.02742067 | 0.05928872 | 0.02910091 | CDKN1A/CDKN2B/MYC | 3 |
| hsa04370 | VEGF signaling pathway | 2/36 | 59/8163 | 0.02764586 | 0.05928872 | 0.02910091 | VEGFA/PTGS2 | 2 |
| hsa04213 | Longevity regulating pathway - multiple species | 2/36 | 62/8163 | 0.03030428 | 0.0642162 | 0.03151948 | IRS2/HSPA8 | 2 |
| hsa04623 | Cytosolic DNA-sensing pathway | 2/36 | 63/8163 | 0.03121232 | 0.06489754 | 0.03185391 | RELA/IL6 | 2 |
| hsa05160 | Hepatitis C | 3/36 | 157/8163 | 0.03135499 | 0.06489754 | 0.03185391 | CDKN1A/RELA/MYC | 3 |
| hsa04630 | JAK-STAT signaling pathway | 3/36 | 162/8163 | 0.03395794 | 0.06947717 | 0.03410174 | CDKN1A/MYC/IL6 | 3 |
| hsa05031 | Amphetamine addiction | 2/36 | 69/8163 | 0.03688308 | 0.07460442 | 0.03661837 | JUN/FOS | 2 |
| hsa04917 | Prolactin signaling pathway | 2/36 | 70/8163 | 0.03786432 | 0.0748872 | 0.03675717 | FOS/RELA | 2 |
| hsa05120 | Epithelial cell signaling in Helicobacter pylori infection | 2/36 | 70/8163 | 0.03786432 | 0.0748872 | 0.03675717 | JUN/RELA | 2 |
| hsa04137 | Mitophagy - animal | 2/36 | 72/8163 | 0.03985679 | 0.07796164 | 0.03826621 | JUN/RELA | 2 |
| hsa01524 | Platinum drug resistance | 2/36 | 73/8163 | 0.04086783 | 0.07907037 | 0.03881041 | CDKN1A/TOP2A | 2 |
| hsa05152 | Tuberculosis | 3/36 | 180/8163 | 0.04423558 | 0.08466596 | 0.04155692 | CEBPB/RELA/IL6 | 3 |
| hsa04621 | NOD-like receptor signaling pathway | 3/36 | 184/8163 | 0.04670972 | 0.08831612 | 0.04334854 | JUN/RELA/IL6 | 3 |
| hsa01521 | EGFR tyrosine kinase inhibitor resistance | 2/36 | 79/8163 | 0.04713501 | 0.08831612 | 0.04334854 | VEGFA/IL6 | 2 |
